# Comparison of Implant Type, Number and Cost, Suture Number, Surgical Time, Clinical Outcomes of Arthroscopic Double-Pulley Suture-Bridge and Single-Row in Repair Supraspinatus Tendon Tears: A Novel Suture-Bridge Technique

**DOI:** 10.1007/s43465-025-01645-6

**Published:** 2025-12-25

**Authors:** Peiguan Huang, Xiaoxu Wang, Yong Fu, Zhengmao Li, Bin Peng, Chunrong He

**Affiliations:** Department of Joint Surgery, Hengyang Medical School, The Second Affiliated Hospital, University of South China, Hengyang, 421001 Hunan China

**Keywords:** Double-pulley suture-bridge, Implant type, Number and cost, Clinical outcomes

## Abstract

**Purpose:**

To compare implant type, number and cost, suture number, surgical time, and clinical outcomes between arthroscopic supraspinatus tendon repair using double-pulley suture-bridge (DPSB) and single-row (SR).

**Methods:**

From December 2016 to August 2022, 87 patients who underwent arthroscopic repair of supraspinatus tendon with either DPSB (*n* = 46) or SR (*n* = 41) were included. Implant type, number and cost, suture number, and surgical time were compared. Clinical outcomes were evaluated with visual analog scale (VAS), American Shoulder and Elbow Surgeons (ASES), University of California, Los Angeles (UCLA) scores, and range of motion (ROM). Magnetic resonance imaging (MRI) or ultrasound was used to assess structural integrity of the tendon.

**Results:**

Suture anchors were the sole implant used in DPSB and SR groups; the surgical costs of DPSB group ($1092) and SR group ($1080) were of no significant difference; the implant cost, the suture number, and the surgical time were of no significant difference. At 24 months of follow-up, VAS, ASES, and UCLA scores and ROM were statistically improved in both groups; however, there were statistical differences between both groups in clinical outcomes of VAS, ASES, UCLA scores and forward flexion, abduction, and internal rotation. On the follow-up of MRI or ultrasound, the overall re-tear rate was 15.6% in DPSB group and 27.2% in SR group at 6 months postoperatively.

**Conclusions:**

DPSB is a novel surgical technique of suture-bridge. Suture anchors were the sole implant used in DPSB and SR groups; DPSB and SR groups achieved comparable implant number and cost, surgical time, and suture number; however, the clinical outcomes of DPSB group were significantly better than SR group, and the re-tear rate of DPSB group was lower than SR group.

**Level of evidence:**

Level III, retrospective control study.

**Supplementary Information:**

The online version contains supplementary material available at 10.1007/s43465-025-01645-6.

## Introduction

The surgical cost-effectiveness refers to the correlation between the cost and the clinical outcomes. Minimizing surgical cost without lowering medical quality is the purpose of optimization cost-effectiveness [1]. Different ways in cost reduction of rotator cuff repair were widely explored [2, 3]. However, the controversy of efficient and inexpensive technique for rotator cuff repair continues [4].

Compared with DR technique, SR repair needs less suture anchor, resulting in the matching reduction of implant cost and surgical time [5]. However, SR repair is called into question over concerns about structural integrity of the reattached tendon [6]. The healing rate of the tendon following SR repair was recorded to be just 43% [7].

DR repair obtains good biomechanics and footprint restoration [8, 9]. Satisfying clinical results [10] and low re-tear rate [11, 12] with DR repair were widely recorded. However, more anchor used and longer surgical time will increase surgical cost [13]. Some scholars believed that DR repair was not cost-effective [4, 13]. The possibility of pullout on lateral-row anchor may happen in the aged [14].

Arthroscopic DPSB repair using double-loaded suture anchor (Twinfix Ti, Smith & Nephew, Andover, MA) as lateral-row anchor is a novel surgical technique. The implant cost was significantly reduced due to the use of inexpensive suture anchor (Twinfix Ti) as lateral-row anchor. The same number of suture crosses the tendon when using the same number of suture anchor in DPSB and SR repair; however, DPSBs can obtain better biomechanics and footprint restoration. Twinfix Ti, which is a type of metallic anchor, can also reduce the risk of anchor pullout.

The purpose of this study was to compare implant type, number and cost, suture number, surgical time, clinical outcomes of arthroscopic DPSB and SR repair of supraspinatus tendon tears. We hypothesized that although there would be no significant differences in implant type, number and cost, suture number, surgical time between both groups, the clinical outcomes of DPSB group were better than SR group, the re-tear rate of the tendon in DPSB repair was lower than that of SR repair.

## Methods

### Patient Selection

**Fig. 1:**
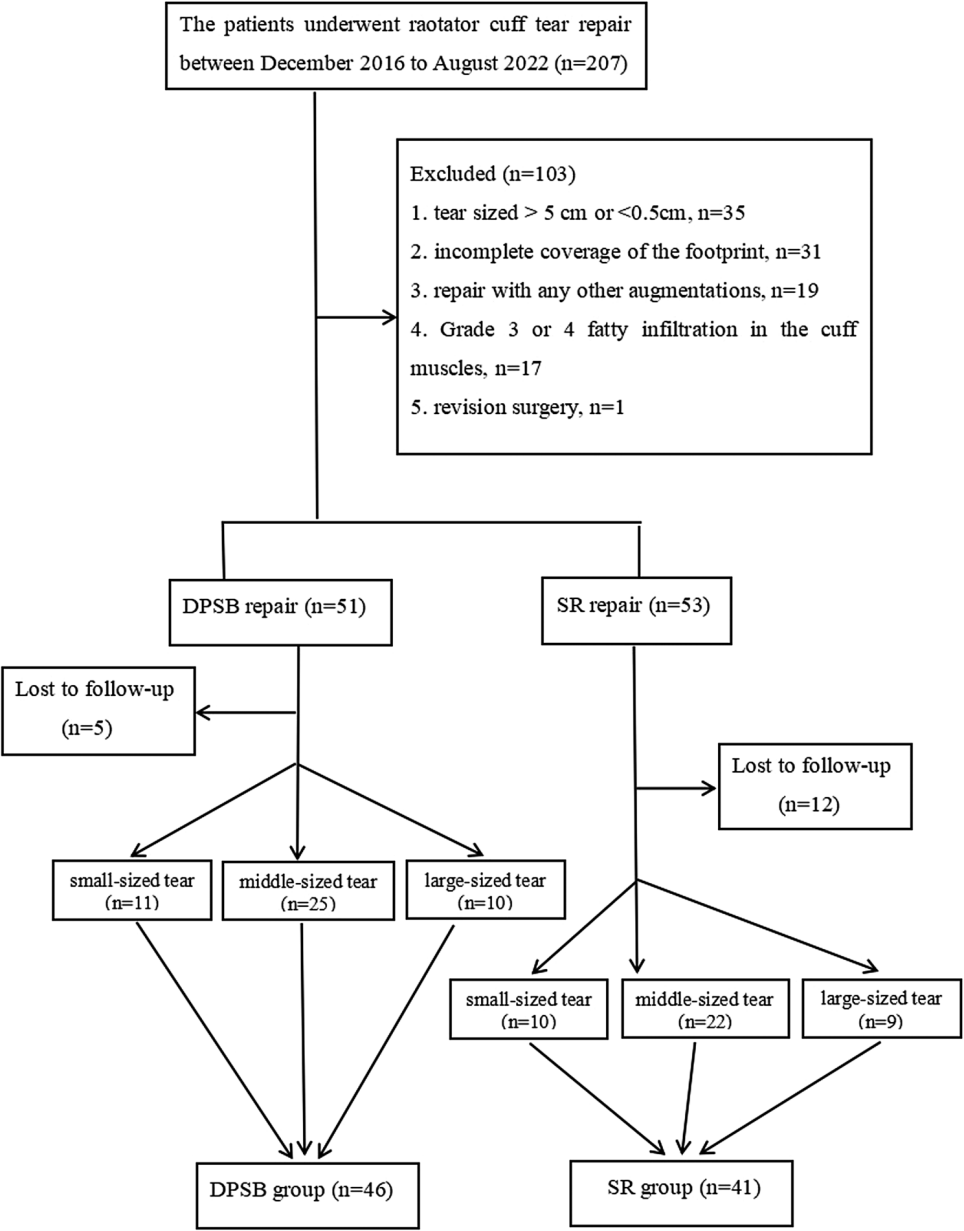
xxxxxxxx

This study was approved by the Institutional Review Board in our Hospital; all patients provided informed consent for participation. From December 2016 to August 2022, 207 patients with supraspinatus tendon tear underwent DPSB or SR repair by one senior shoulder surgeon (Huang) in our hospital. The patient allocation was not randomized, SR repair was performed before August 2021 and DPSB repair later on. The inclusion criteria were: (1) small-sized (0.5–1.0 cm), middle-sized (1–3 cm), and large-sized (3–5 cm) supraspinatus tendon tears [15] and confirmed with arthroscopic evaluation, (2) repair with DPSB or SR technique, (3) full-thickness tendon tear was confirmed in the operation, (4) original footprint could be covered by the reattached tendon, (5) the final follow-up duration was no less than 24 months postoperatively. We excluded: (1) tear sized > 5 cm or < 0.5 cm, (2) incomplete coverage of the footprint, (3) combined with subscapularis and infraspinatus tears. (4) repair with any other augmentations, (5) Grade 3 or 4 fatty infiltration in the cuff muscles, (6) revision surgery, (7) incomplete follow-up data. All data were collected retrospectively, and 87 patients (46 in DPSB group and 41 in SR group) who conformed to the inclusion and exclusion criteria were enrolled in the study (Fig. 1).

### Surgical Procedures

Suture anchors (Twinfix Ti) were the sole implants for DPSB and SR groups.

In SR group, suture anchors were inserted into lateral margin of the greater tuberosity. Tear size of the tendon determined the anchor number: 1–2 anchors were used in small-sized tendon tears; 2–3 anchors were used in middle-sized tendon tears; 3–5 anchors were used in large-sized tendon tears. One suture strand of each anchors was passed through the tendon and firmly knotted.

**Fig. 2:**
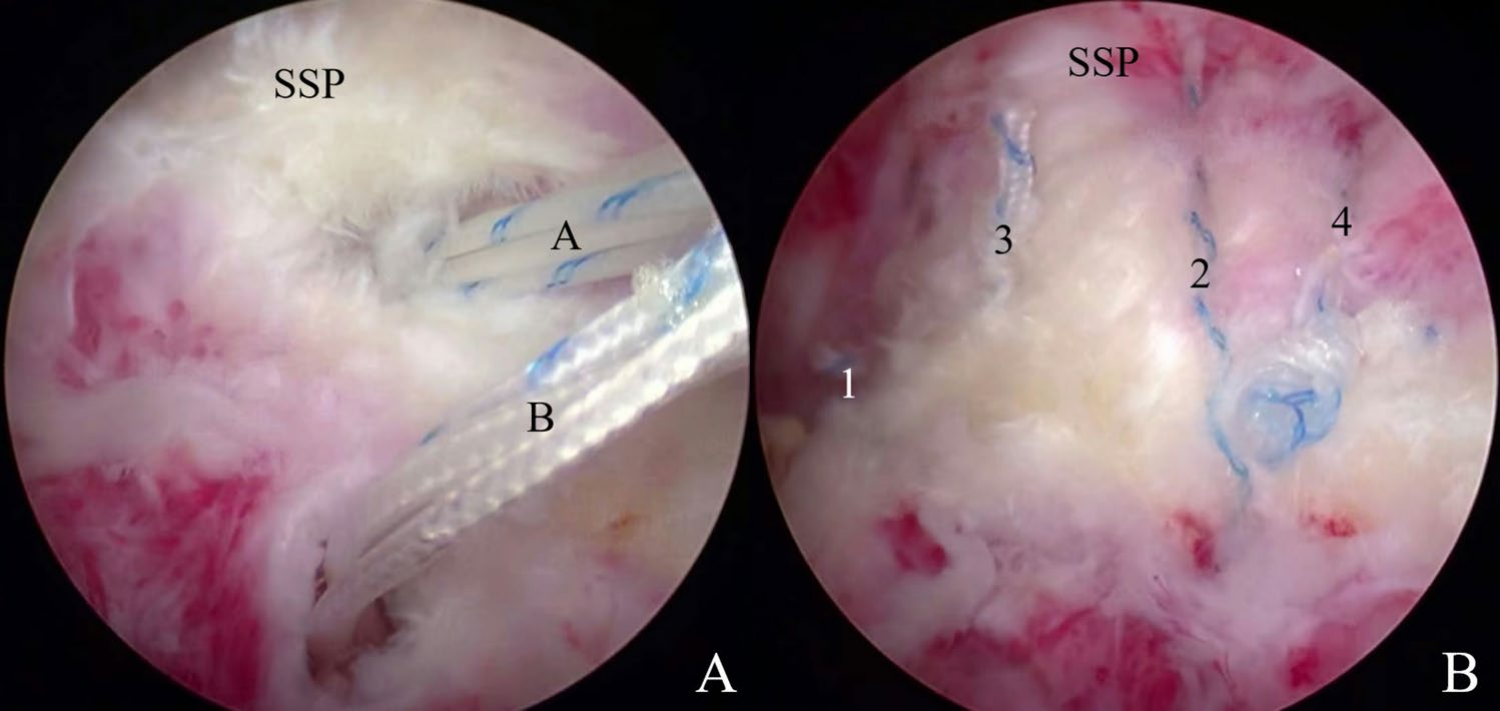
Arthroscopic image of right shoulder viewed through the sub-acromial lateral portal showing: **A** 2 suture anchors, which are named anchor A (medial-row anchor) and anchor B (lateral-row anchor), were inserted in the greater tuberosity. **B** 4 set of DPSB powerfully compressed the supraspinatus tendon (SSP) against the footprint. (A, the strands from anchor A; B, the strands from anchor B; numbers 1–4, the first to fourth set of DPSBs)

**Fig. 3:**
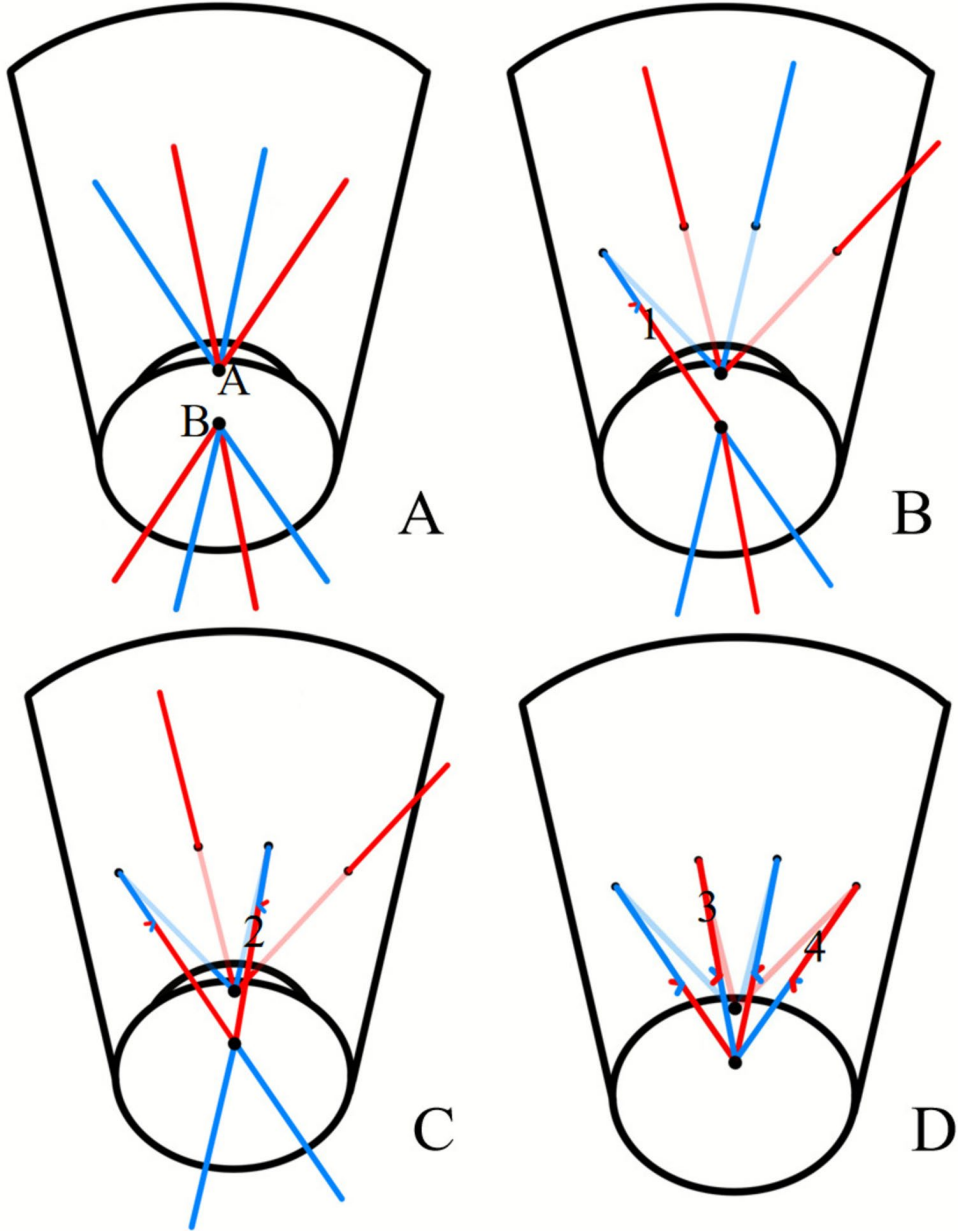
Illustration of two-anchor DPSB showing: **A** anchor A and B are inserted in the greater tuberosity; **B** the first set of DPSB (1) is created; **C** the second set of DPSB (2) is created; **D** 4 set of DPSBs powerfully compress the supraspinatus tendon against the footprint. (A, anchor A; B, anchor B; numbers 1–4, the first to fourth set of DPSB)

In DPSB group with two anchors, two suture anchors, which were named anchor A (medial-row anchor) and anchor B (lateral-row anchor), were inserted in the greater tuberosity (Fig. 2A). 4 medial strands from anchor A were passed through the tendon. One blue strand from anchor A and one white strand from anchor B were grabbed out of the body and created into the first set of DPSB. As a result of double-pulley, this DPSB was delivered into the sub-acromial space. The opposite blue and white strands were created into the second set of DPSB. The white suture from anchor A and the blue suture from anchor B were created into the third and fourth sets of DPSBs (Fig. 2B). The surgical procedures are presented in Fig. 3 and described by Huang et al. [16].

**Fig. 4:**
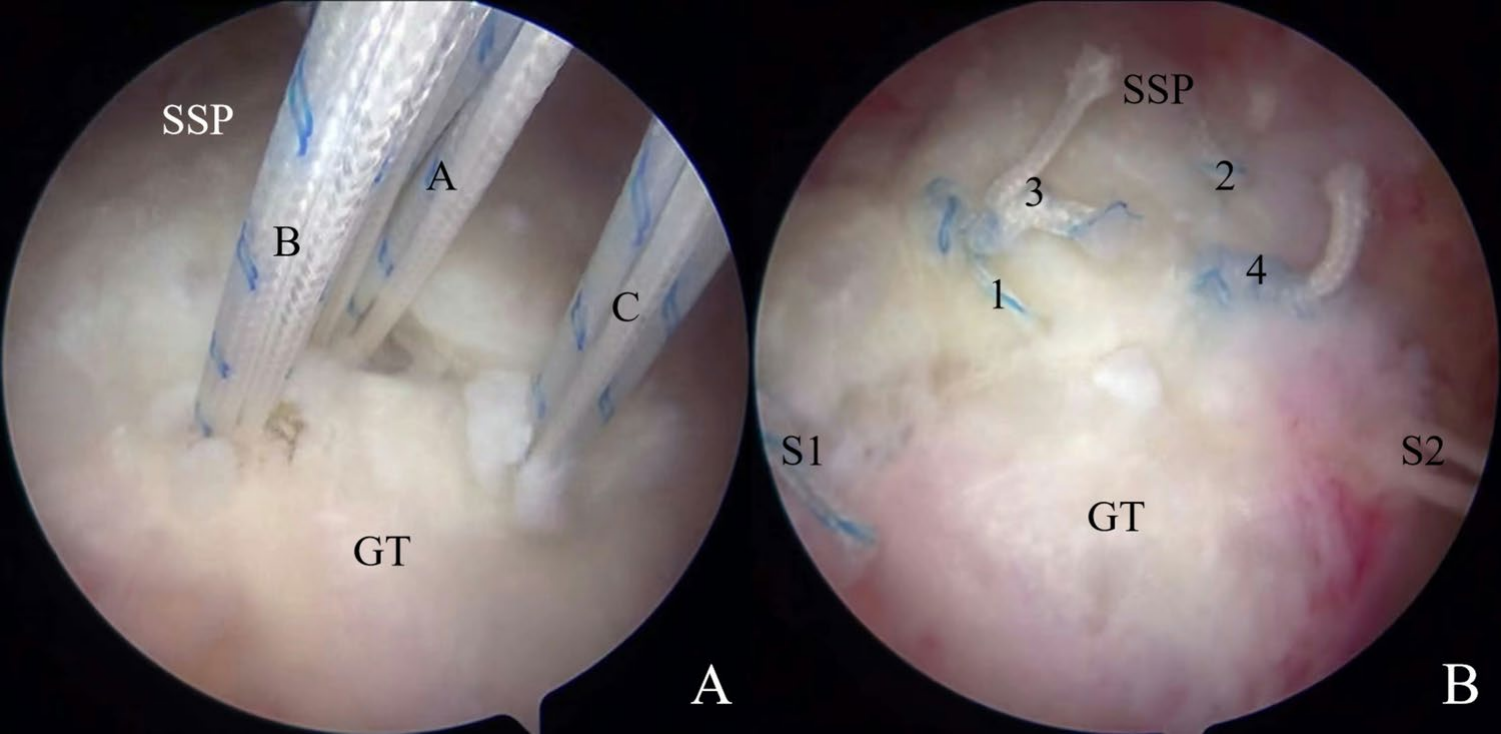
Arthroscopic image of right shoulder viewed through the sub-acromial lateral portal showing: **A** 3 suture anchors, which were named anchor A (medial-row anchor), anchor B and C (lateral-row anchors), were inserted in the greater tuberosity (GT). **B** 4 set of DPSBs and 2 set of SRs powerfully compressed the supraspinatus tendon (SSP) against the footprint. (A, the strands from anchor A; B, the strands from anchor B; C, the strands from anchor C; numbers 1–4, the first to fourth set of DPSBs; S1-2, the first and second set of single-rows)

**Fig. 5:**
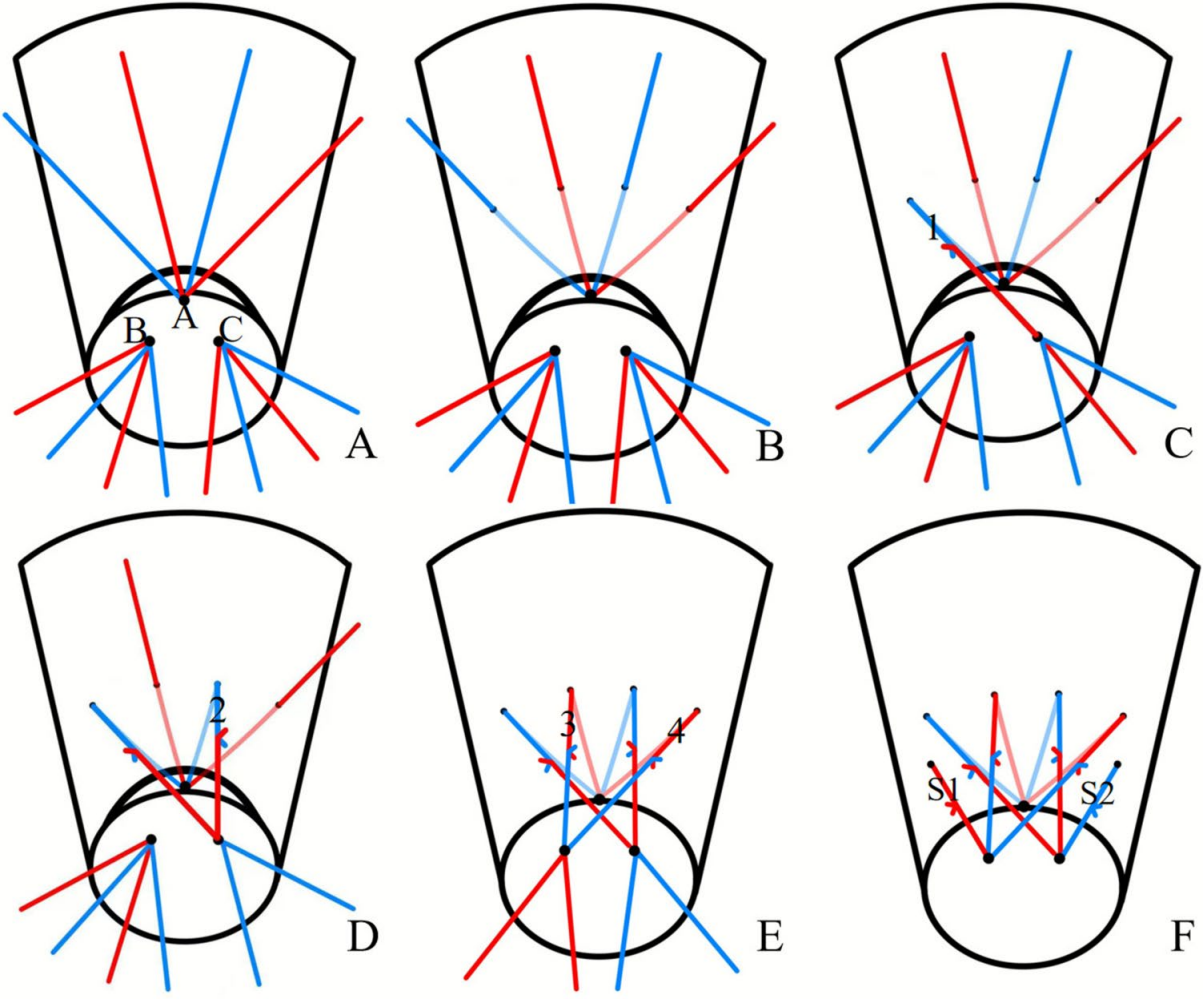
Illustration of tri-anchor DPSB showing: **A** 3 suture anchors, which were named anchor A (medial-row anchor), anchor B and C (lateral-row anchors), were inserted in the greater tuberosity; **B** 4 strands from anchor A are passed through the tendon alternating between blue and white; **C** the first set of DPSB (1) is created; **D** the second set of DPSB (2) is created; **E** the third (3) and fourth (4) set of DPSBs are created; **F** 4 set of DPSBs and 2 set of SRs (S1,S2) are created and powerfully compressed the tendon against the footprint. (A, anchor A; B, anchor B; C, anchor C)

In DPSB group with three anchors, three suture anchors, which were named anchor A (medial-row anchor), anchors B and C (lateral-row anchors), were inserted in the greater tuberosity (Fig. 4A). 4 medial strands from anchor A were passed through the tendon. One blue strand from anchor A and one white strand from anchor C were grabbed out of the body and created into the first set of DPSB. As the result of double-pulley, this DPSB was delivered into the sub-acromial space. The opposite blue and white strands were created into the second set of DPSB. The white suture from anchor A and the blue suture from anchor B were created into the third and fourth sets of DPSBs. The white suture from anchor B and the blue suture from anchor C were created into the first and second sets of single rows (Fig. 4B). The surgical procedures are presented in Fig. 5 and described by Huang et al. [17].

**Fig. 6:**
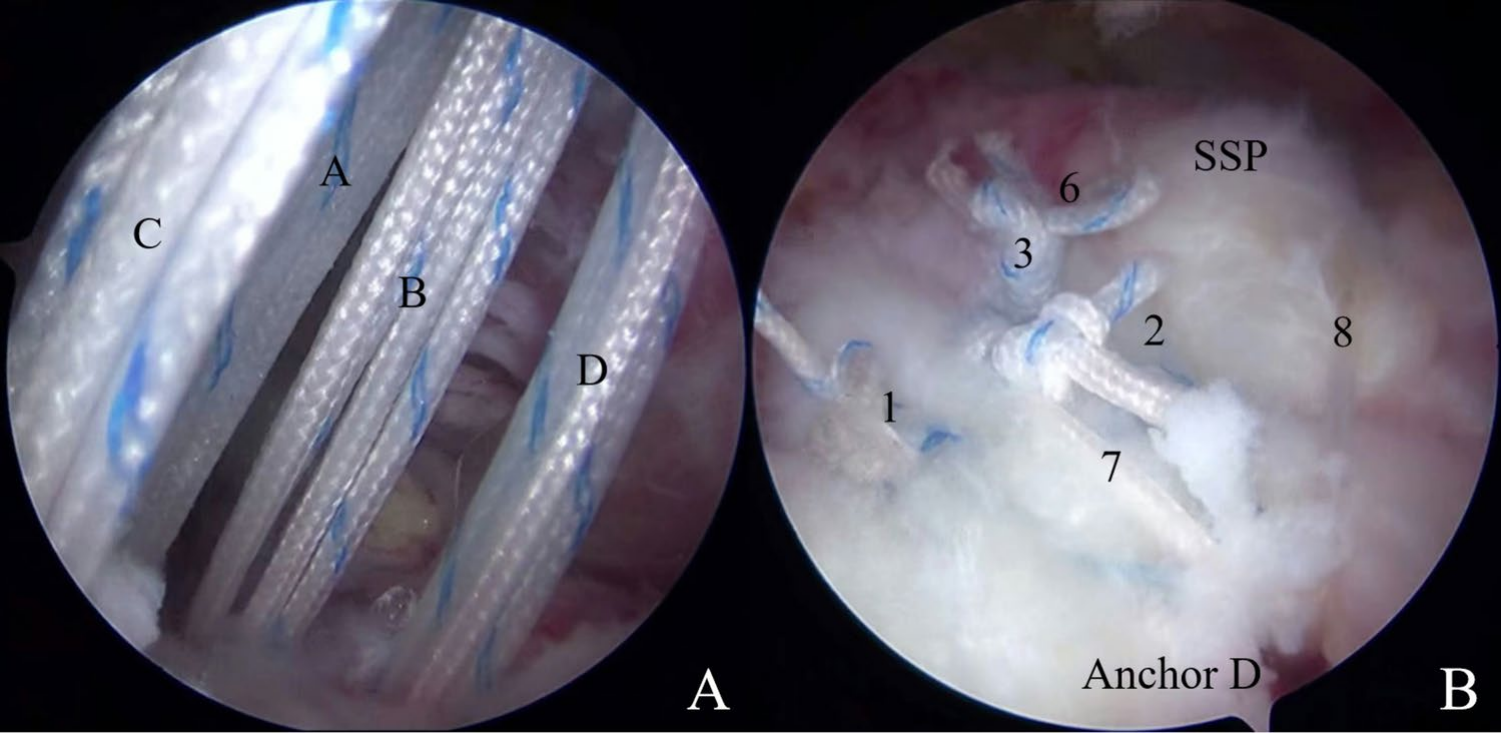
Arthroscopic image of right shoulder viewed through the subacromial lateral portal showing: **A** Four suture anchors, which were named anchor A, B (medial-row anchors) and anchor C, D (lateral-row anchors), were inserted in the greater tuberosity. **B** 8 set of DPSBs powerfully compressed the supraspinatus tendon (SSP) against the footprint. (A, the strands from anchor A; B, the strands from anchor B; C, the strands from anchor C; D, the strands from anchor D; numbers 1–8, the first to eighth set of DPSB)

**Fig. 7:**
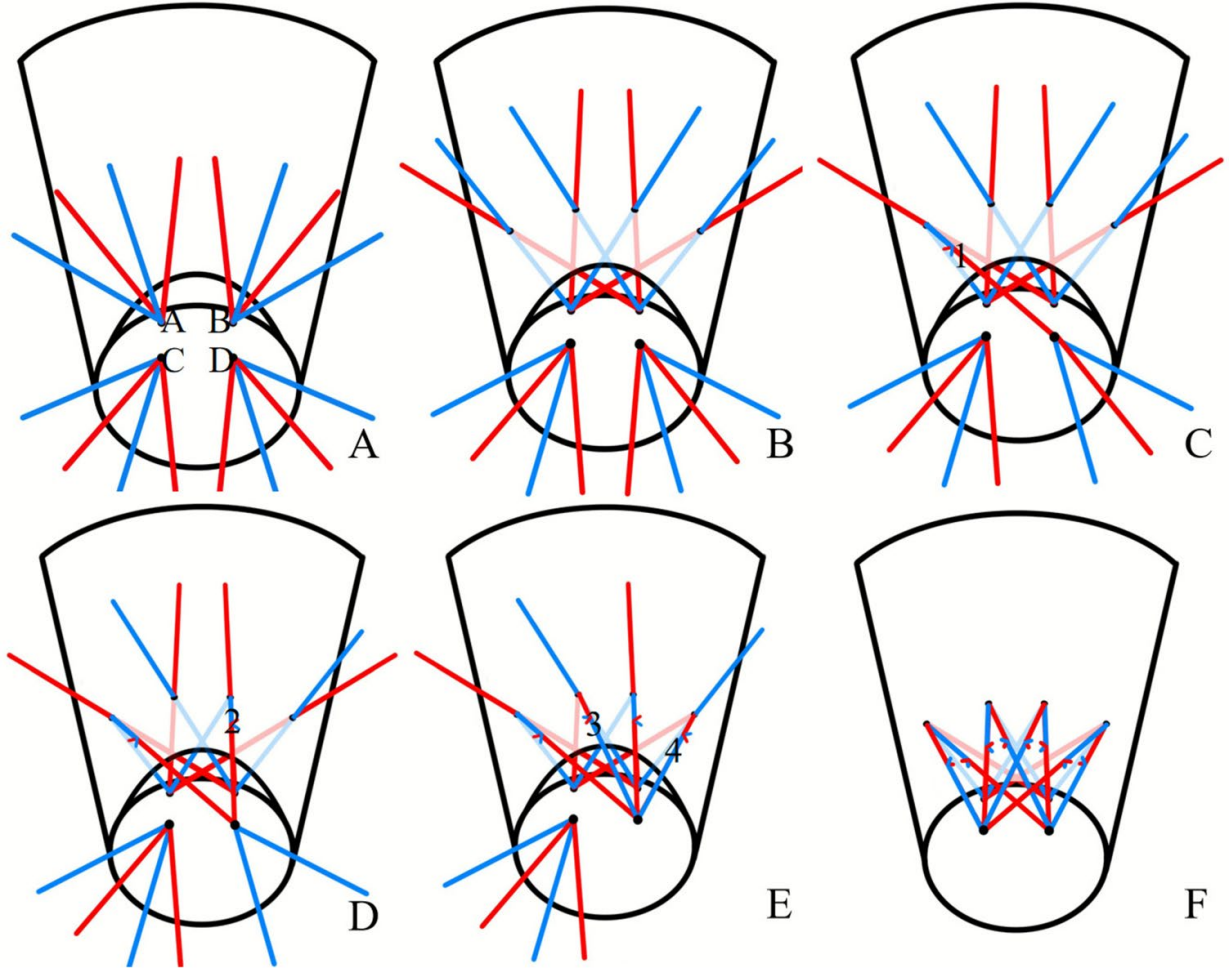
Illustration of four-anchor DPSB showing: **A** four suture anchor, which are named anchor A, B (medial-row anchors) and anchor C, D (lateral-row anchors), were inserted in the greater tuberosity.; **B** medial strands from anchor A and B are passed through the tendon alternating between blue and white; **C** the first set of DPSB (1) is created; **D** the second set of DPSB (2) is created; **E** The third (3) and fourth (4) set of DPSBs is created; **F** 8 set of DPSBs powerfully compressed the tendon against the footprint. (A, anchor A; B, anchor B; C, anchor C; D, anchor D)

In DPSB group with four anchors, four suture anchors, which were named anchors A, B (medial-row anchors) and anchors C, D (lateral-row anchors), were inserted in the greater tuberosity (Fig. 6A). 8 strands from anchors A and B were passed through the tendon alternating between blue and white. One blue suture strand of anchor A and one white strand from anchor D were retrieved out of the body and created into the first set of DPSB. As a result of double-pulley, this DPSB was delivered into the sub-acromial space. The opposite blue and white strands were created into the second set of DPSB. The white suture from anchor A and the blue suture from anchor D were created into the third and fourth sets of DPSBs. Sutures from anchors B and D were created into other 4 sets of DPSBs (Fig. 6B). The surgical details are presented in Fig. 7 and described by Huang et al. [18].

### Implant Type, Number and Cost, Suture Number, and Surgical Time

Suture anchors (Twinfix Ti, USA: $373) were the sole implants for DPSB and SR groups.

Small-sized tendon tears involved 2 or 3 suture anchors in DPSB group and 1 or 2 suture anchors in SR group; middle-sized tendon tears involved 2 or 3 suture anchors in DPSB group and 2 or 3 suture anchors in SR group; large-sized tendon tears involved 3 or 4 suture anchors in DPSB group and 3, 4 or 5 suture anchors in SR group.

Small-sized tendon tears involved 4 or 6 set of sutures containing DPSBs and SRs in DPSB group and 2 or 4 set of SRs in SR group; middle-sized tendon tears involved 4 or 6 set of sutures containing DPSBs and SRs in DPSB group and 4 or 6 set of SRs in SR group; large-sized tendon tears involved 6 or 8 set of sutures containing DPSBs and SRs in DPSB group and 6, 8 or 10 set of SRs in SR group.

According to the anesthesia records, surgical time was recorded the time from shoulder incision to wound closure.

### Clinical Evaluation

Clinical evaluations were performed preoperatively and 24 months postoperatively. A doctor who did not participate in this study collected clinical outcomes. The scores of VAS, ASES, and UCLA were recorded. ROM was assessed in forward flexion, abduction, and internal and external rotations. Internal rotation was measured as the topmost vertebral level could touch by thumb tip. The vertebral level was converted to numbers for statistical evaluation: C1 to C7 were recorded 1 to 7, T1 to T12 were recorded 8 to 19, L1 to L5 were recorded 20 to 24, sacrum was recorded 25, and buttock was recorded 26 [19, 20]. Surgical satisfaction was rated from 1 to 5: 5, very satisfied; 4, satisfied; 3, rather satisfied; 2, rather unsatisfied; and 1, unsatisfied.

### Tendon Integrity Evaluation

3.0 T MRI scanner was used to evaluate structural integrity of the tendon. Sugaya classification was used to rate healing status of the tendon, types IV and V were recorded as tendon re-tear [21]. Ultrasound was another way to assess healing status of the tendon, type II (mild discontinuity or focal defect of the tendon) and type III (remarkable discontinuity or full-thickness tear of the tendon) were recorded as tendon re-tear.

### Postoperative Rehabilitation

Rehabilitation programs in DPSB and SR groups was selfsame. The arm was fixed with abduction brace 6 weeks postoperatively. Passive forward flexion of the joint in supine position was initiated 2 days postoperatively. Active ROM exercise was initiated 6 weeks postoperatively. Home ROM exercises continued 3 month postoperatively. Premorbid levels of sport activities were initiated 6 months postoperatively.

### Statistical Analysis

SPSS (v 22.0; IBM Corp) was used for statistical analysis. Pearson chi-square test was used to compare sex and tear size between groups. Mann–Whitney U test was used to compare mean age, symptom period and follow-up duration, implant number and cost, suture number, surgical time, postoperative values within VAS, ASES, UCLA scores, and ROM. Wilcoxon signed-rank test was used to compare pre- and postoperative values within VAS, ASES, UCLA scores, and ROM. Fisher's exact test was used to compare retear rate of the tendon. Statistical significance was set at *P* < 0.05.

Post hoc power analysis (SPSSAU: https://spssau.com/index.html) was performed to eliminate type II errors when the significant difference was found between DPSB and SR groups, the power to detect a significant difference was 0.80 with an alpha error of 0.05.

## Results

### Patient Characteristics

The mean follow-up was 28.1 (24–35 months) in DPSB and 27.1 months (24–33 months) in SR group. The patient characteristics of sex, mean age, symptom duration, follow-up duration, and tear size were not significantly different between DPSB and SB groups (Table 1).

**Table 1:** Characteristics of Patients in DPSB and SR Groups

	DPSB group (95% CI) (*n* = 46)	SR group (95% CI) (*n* = 41)	P Value
Sex, n			
Male	26	33	0.789 ②
Female	20	19	
Mean age, years	61.0 ± 8.7	63.9 ± 8.7	0.108 ①
Symptom period, mo	9.7 ± 8.3	7.7 ± 6.3	0.132 ①
Follow-up, mo	29.5 ± 4.1	29.1 ± 2.7	0.867 ①
	(24–33)	(24–33)	
Tear size, n			
Small-sized tear	11	10	
Middle-sized tear	25	22	
Large-sized tear	10	9	

DPSB group, the group of double-pulley suture-bridge

SR group, the group of single-row

Values are given as mean ± SD

Data are presented as mean (95% CI)

① P values were calculated using the Mann–Whitney U test

② P values were calculated using Pearson chi-square test

### Implant Number and Cost, Suture Number, and Surgical Time

Small-sized tendon repair required the mean of 2.0 suture anchors in DPSB group and 1.8 suture anchors in SR group; middle-sized tendon repair required the mean of 2.9 suture anchors in DPSB group and 2.8 suture anchors in SR group; large-sized tendon repair required the mean of 3.9 suture anchors in DPSB group and 4.1 suture anchors in SR group. DPSB group required the mean of 2.9 suture anchors and SR group required the mean of 2.9 suture anchors. All of the above data were of no significant difference between both groups.

Small-sized tendon repair required the mean costs of $779 in DPSB group and $671 in SR group; middle-sized tendon repair required the mean cost of $1086 in DPSB group and $1065 in SR group; large-sized tendon repair required the mean cost of $1450 in DPSB group and $1530 in SR group. The mean cost of implant was required $1092 in DPSB group and $1080 in SR group. All of the above data were of no significant difference between both groups.

Small-sized tendon tear was repaired with 4.1 set of sutures containing DPSBs and SRs in DPSB group and 3.6 set of SRs in SR group; middle-sized tendon tear was repaired with 5.8 set of sutures containing DPSBs and SRs in DPSB group and 5.7 set of SRs in SR group; large-sized tendon tear was repaired with 7.8 set of sutures containing DPSBs and SRs in DPSB group and 8.2 set of SRs in SR group. The mean suture number used for tendon repair was 5.8 set of sutures containing DPSBs and SRs in DPSB group and 5.7 set of SRs in SR group. All of the above suture numbers were of no significant difference between both groups.

Small-sized tendon repair required the mean surgical time of 70.5 min in DPSB group and 66.7 min in SR group; middle-sized tendon repair required the mean surgical time of 90.6 min in DPSB group and 88.2 min in SR group; large-sized tendon repair required the mean surgical time of 97.1 min in DPSB group and 95.2 min in SR group. The mean surgical time was required 87.4 min in DPSB group and 84.2 min in SR group. All of the above data were of no significant difference between both groups (Table 2).

**Table 2:** Implant Number and Cost, Suture Numbers, and Surgical Time in DPSB and SR Groups

	DPSB group (95% CI) (*n* = 46)	SR group (95% CI) (*n* = 41)	P Value
Implant number			
Small-sized tear	2.0 ± 0.3	1.8 ± 0.4	0.083
Middle-sized tear	2.9 ± 0.2	2.8 ± 0.3	0.536
Large-sized tear	3.9 ± 0.3	4.1 ± 0.6	0.330
Total	2.9 ± 0.6	2.9 ± 0.9	0.669
Implant cost ②			
Small-sized tear	779.6 ± 111.5	671.8 ± 157.2	0.083
Middle-sized tear	1086.4 ± 102.4	1065.5 ± 129.9	0.536
Large-sized tear	1450.8 ± 117.6	1530.4 ± 225.3	0.330
Total	1092.2 ± 252.2	1080.4 ± 330.6	0.669
Suture number			
Small-sized tear	4.1 ± 0.6	3.6 ± 0.8	0.083
Middle-sized tear	5.8 ± 0.5	5.7 ± 0.7	0.536
Large-sized tear	7.8 ± 0.6	8.2 ± 1.2	0.330
Total	5.8 ± 1.3	5.7 ± 1.8	0.669
Surgical time ③			
Small-sized tear	70.5 ± 5.3	66.7 ± 1.6	0.142
Middle-sized tear	90.6 ± 3.6	88.2 ± 4.5	0.064
Large-sized tear	97.0 ± 4.2	95.2 ± 6.1	0.241
Total	87.4 ± 10.2	84.2 ± 11.3	0.105

DPSB group, the group of double-pulley suture-bridge

SR group, the group of single-row

Values are given as mean ± SD

Data are presented as mean (95% CI)

① P values were calculated using the Mann–Whitney U test

② The number 1 of implant cost means 1 dollar

③ The number 1 of surgical time means 1 min

### Range of Motion

In DPSB and SR group, the preoperative forward flexion, the abduction, and the external and internal rotations were all significantly improved at the final follow-up (*P* < 0.001, *P* < 0.001, *P* < 0.001, and *P* < 0.001, respectively, with power of 1.0, 1.0, 1.0, and 1.0, respectively, in DPSB group and *P* < 0.001, *P* < 0.001, *P* < 0.001, and *P* = 0.001, respectively, with power of 1.0, 1.0, 1.0, and 1.0, respectively, in SR group).

The postoperative scores of forward flexion, abduction, and internal rotation between DPSB and SR group were of significant difference (*P* = 0.027, *P* = 0.017, and *P* = 0.029, respectively) (Table 3).

**Table 3:** Range of Motion, Clinical Outcomes, and Satisfaction in DPSB and SR Groups

	DPSB group (95% CI) (*n* = 46)			SR group (95% CI) (*n* = 41)			DPSB VS SR
	Preoperative	Postoperative	P value②	Preoperative	Postoperative	P value②	P value①
FF	87.3 ± 27.6	156.3 ± 27.6	< 0.001	82.2 ± 26.0	150.1 ± 25.9	< 0.001	0.027
ABD	68.8 ± 35.3	156.9 ± 27.0	< 0.001	63.2 ± 25.6	153.5 ± 16.2	< 0.001	0.017
ER	28.6 ± 7.5	47.4 ± 11.1	< 0.001	29.5 ± 7.7	47.5 ± 10.1	< 0.001	0.848
IR	21.7 ± 3.0	16.8 ± 3.1	< 0.001	21.9 ± 2.6	18.2 ± 3.4	0.001	0.029
VAS	7.7 ± 1.3	1.4 ± 0.5	< 0.001	7.9 ± 1.2	1.7 ± 0.6	< 0.001	0.011
ASES	28.2 ± 9.7	90.7 ± 5.6	< 0.001	28.8 ± 7.9	89.7 ± 3.7	< 0.001	0.005
UCLA	10.2 ± 3.3	31.2 ± 2.8	< 0.001	10.5 ± 3.2	29.7 ± 3.5	< 0.001	0.030
Satisfaction	0	4.5 ± 0.8		0	4.1 ± 0.8		0.011

DPSB group, the group of double-pulley suture-bridge

SR group, the group of single-row

Values are given as mean ± SD

Data are presented as mean (95% CI)

FF, forward flexion; ABD, abduction; ER, external rotation; IR, internal rotation; VAS score, Visual analog scale pain score; ASES score, American Shoulder and Elbow Surgeon score; UCLA, University of California, Los Angeles score

Internal rotation was considered as the topmost vertebral level could reach by thumb tip. The vertebral level was transformed to numbers for statistical analysis: C1 to C7 were noted 1 to 7, T1 to T12 were noted 8 to 19, L1 to L5 were noted 20 to 24, sacrum was noted 25, and buttock was noted 26

① P values were calculated using the Mann–Whitney U test

② P values were calculated using the Wilcoxon signed-rank test

### Clinical Outcomes

In DPSB and SR group, the preoperative scores of VAS, ASES, and UCLA were all significantly improved at the final follow-up (*P* < 0.001, *P* < 0.001, and *P* < 0.001, respectively, with power of 1.0, 1.0, and 1.0, respectively in DPSB group and *P* < 0.001, *P* < 0.001 and *P* < 0.001, respectively, with power of 1.0, 1.0, and 1.0, respectively, in SR group).

The postoperative scores of VAS, ASES, and UCLA between DPSB and SR group were of significant difference (*P* = 0.011, *P* = 0.005, and *P* = 0.030, respectively) (Table 3).

### MRI and Ultrasound Evaluation

At 6 months of follow-up, 23 patients (50.0%) of DPSB group underwent MRI examination, 1 patients could not perform MRI due to the contraindication of conventional pacemaker, and 22 patients refused it. 9 (40.9%) of the 22 patients underwent ultrasound examination, 12 patients refused any radiologic follow-up. Overall, 32 patients (89%) underwent MRI or ultrasound examination in DPSB group. 12 patients (29.2%) of SR group underwent MRI examination, 29 patients refused it. 10 (34.4%) of the 29 patients underwent ultrasound examination, 19 patients refused any radiologic follow-up. Overall, 22 patients (53.6%) underwent MRI or ultrasound examination in SR group.

Sugaya type IV or V tendon re-tears with MRI scan were observed in 4 of 23 patients of DPSB group; types II and III tendon re-tear with ultrasound were observed in 1 of 9 patients of DPSB group. The total re-tear of the tendon was observed in 5 of 32 (15.6%) patients of DPSB group. Sugaya type IV or V tendon re-tears with MRI scan were observed in 3 of 12 patients of SR group; types II and III tendon re-tear with ultrasound were observed in 3 of 10 patients of SR group. The total re-tears of the tendon were observed in 6 of 22 (27.2%) patients of SR group (Table 4).

**Table 4:** Follow-Up of Re-tear Rate in DPSB and SR Groups

	DPSB group (*n* = 32)	SR group (*n* = 22)
Sugaya classification		
Type I	11	6
Type II	5	2
Type III	3	1
Type IV	2	1
Type V	2	2
MRI re-tear number,	4	3
Ultrasound classification		
Type I	8	7
Type II	1	1
Type III	0	2
Ultrasound re-tear number	1	3
Total re-tear rate	15.6%	27.2%

DPSB group, the group of double-pulley suture-bridge

SR group, the group of single-row

## Discussion

DPSB is a novel surgical technique of suture bridge. Suture anchors were the sole implant used in DPSB and SR groups; DPSB and SR groups achieved comparable implant number and cost, surgical time, and suture number; however, the clinical outcomes of DPSB group were significantly better than SR group, and re-tear rate of DPSB group was lower than SR group.

The great cost of rotator cuff repair needs to be focused on [22]. The number of anchor used is the primary factor of surgical cost in cuff repair [3, 23]. Compared with SR method, DR repair usually needs more anchors when treating a similar size of cuff tear [24]. Given the expensive cost of lateral-row anchor, the additional row anchor is not an economically choice in DR repair [13]. It is essential for surgeons to lower the surgical cost without compromising clinical outcomes [25]. DPSB repair presented in the study, using suture anchor as lateral-row anchor instead of the expensive conventional lateral-row, can significantly reduce the surgical cost; there was no significant difference of an implant cost between DPSB and SR repair in our results.

Surgical time is also crucial for the surgical cost of rotator cuff repair [1, 2]. The surgical cost of suture-bridge repair is evenly shared by surgical time and implant cost [9]. Each minute in the operating room increases 47 dollars [2]. Shorter surgical time can be achieved using SR fixation [26]. Franceschi et al. [24] expounded the mean surgical time for DR repair was 65 ± 23.4 min, while SR repair was 42 ± 18.9 min. Nevertheless, lesser anchor used is the reason why reduced surgical time can be obtained in SR fixation [27], but lesser anchor used may result in poor clinical results. In our study, surgical time between DPSB and SR repair was not significantly different because suture passing the tendon and strand management were the major procedures that consumed surgical time.

The durability of tendon relies on the number of suture crossing the tendon [28]. Greater tendon strength and lower re-tear rate can achieve when applying more suture [29, 30]. The ratio of suture number to tendon volume is important for good surgical outcomes [31]. The ovine model established the incremental number of suture crossing the tendon can significantly increase the load of tendon failure [32–34]. Better biomechanics of DR repair is relevant to more suture crossing the tendon rather than the addition of row anchor [35, 36]. In our study, the approximately identical number of suture crossing the tendon can be obtained in DPSB and SR repair; however, DPSBs can obtain better biomechanics and footprint restoration.

The pullout of lateral anchor is a troublesome complication in DR repair [14]. Barber et al. [37] considered lateral-row anchor offered inferior fixation strength in cyclic loading. Tsiouri et al. [38] reported lateral-row anchor failed 2 weeks postoperatively. The material of anchor decides the stability of anchor fixation [39]. Barber et al. [40] found metallic anchor was to be the most hard anchor in porcine bone. Tingart et al. [41] reported the failure load of biodegradable anchor was as low as 112 N in lateral portion of greater tuberosity, compared with 184 N for metallic anchor. Yang et al. [42] noted metallic anchor possessed the least displacement. In our study, Twinfix Ti is the sole implant and the risk of anchor pullout was reduced compared with conventional lateral-row anchor.

In summary, arthroscopic DPSB repair using suture anchor as lateral-row anchor can obtain the following features: first, inexpensive suture anchor (Twinfix Ti) used as lateral-row anchor can reduce the implant cost. Second, surgical time between DPSB and SR repair was not of significant statistical difference. Third, the same number of suture crossing the tendon can be obtained when using the same number of Twinfix Ti in DPSB and SR repair; but DPSBs can obtain better biomechanics and footprint restoration. Fourth, the risk of lateral-row anchor pullout is reduced because Twinfix Ti, which is used as a lateral-row anchor, is a type of metallic anchor.

Even so, several limitations have to be noted. First, the patients were not randomized assignment, SR repair was performed before August 2022, and DPSB repair later on. Second, the rejection of MRI follow-up in some patients prevented us from assessment on longer-term prognosis. Third, ultrasound is reliable and cost-effective in the evaluation of repaired tendon; but the problems, such as operator dependence, specificity, and sensitivity, of the diagnosis also exist. Fourth, the follow-up duration of MRI and ultrasound was 6 months postoperatively, which will reduce the evidence quality for the conclusion. Fifth, a chance of observer bias in collecting the postoperative scores and ROM. Sixth, the histological evaluation, which is the gold standard for postoperative tendon healing, was not performed. Seventh, the patient sample of the study is small, and more sample and multicenter size are necessary for more objective results.

## Conclusions

DPSB is a novel surgical technique of suture bridge. Suture anchors were the sole implant used in DPSB and SR groups; DPSB and SR groups achieved comparable implant number and cost, surgical time, and suture number; however, the clinical outcomes of DPSB group were significantly better than SB group, and re-tear rate of DPSB group was lower than SR group.

## Supplementary Information

Below is the link to the electronic supplementary material.

Supplementary file1 (PNG 43 KB)

Supplementary file2 (PNG 46 KB)

Supplementary file3 (PNG 39 KB)

Supplementary file4 (PNG 33 KB)

Supplementary file5 (PNG 30 KB)

Supplementary file6 (PNG 34 KB)

Supplementary file7 (PNG 31 KB)

Supplementary file8 (PNG 30 KB)

Supplementary file9 (PNG 30 KB)

Supplementary file10 (PNG 37 KB)

Supplementary file11 (PNG 33 KB)

Supplementary file12 (PNG 34 KB)

Supplementary file13 (PNG 32 KB)

Supplementary file14 (PNG 47 KB)

Supplementary file15 (PNG 31 KB)

Supplementary file16 (PNG 30 KB)

Supplementary file17 (PNG 42 KB)

Supplementary file18 (PNG 38 KB)

Supplementary file19 (PNG 33 KB)

Supplementary file20 (PNG 39 KB)

Supplementary file21 (PNG 31 KB)

Supplementary file22 (PNG 30 KB)

Supplementary file23 (PNG 34 KB)

Supplementary file24 (PNG 48 KB)

Supplementary file25 (PNG 30 KB)

Supplementary file26 (PNG 43 KB)

Supplementary file27 (PNG 28 KB)

Supplementary file28 (PNG 39 KB)

Supplementary file29 (PNG 49 KB)

Supplementary file30 (PNG 29 KB)

Supplementary file31 (PNG 32 KB)

Supplementary file32 (PNG 32 KB)

Supplementary file33 (PNG 46 KB)

Supplementary file34 (PNG 39 KB)

Supplementary file35 (PNG 39 KB)

Supplementary file36 (PNG 60 KB)

Supplementary file37 (PNG 29 KB)

Supplementary file38 (PNG 28 KB)

Supplementary file39 (PNG 41 KB)

Supplementary file40 (PNG 43 KB)

Supplementary file41 (PNG 44 KB)

Supplementary file42 (PNG 44 KB)

Supplementary file43 (PNG 47 KB)

Supplementary file44 (PNG 48 KB)

Supplementary file45 (PNG 42 KB)

Supplementary file46 (PNG 41 KB)

Supplementary file47 (PNG 39 KB)

Supplementary file48 (PNG 35 KB)

Supplementary file49 (PNG 37 KB)

Supplementary file50 (PNG 38 KB)

Supplementary file51 (PNG 35 KB)

Supplementary file52 (PNG 34 KB)

Supplementary file53 (PNG 34 KB)

Supplementary file54 (PNG 36 KB)

Supplementary file55 (PNG 40 KB)

Supplementary file56 (PNG 36 KB)

Supplementary file57 (PNG 31 KB)

Supplementary file58 (PNG 45 KB)

Supplementary file59 (PNG 36 KB)

Supplementary file60 (PNG 37 KB)

Supplementary file61 (PNG 35 KB)

Supplementary file62 (PNG 48 KB)

Supplementary file63 (PNG 32 KB)

Supplementary file64 (PNG 47 KB)

Supplementary file65 (PNG 31 KB)

Supplementary file66 (PNG 32 KB)

Supplementary file67 (PNG 34 KB)

Supplementary file68 (PNG 39 KB)

Supplementary file69 (PNG 38 KB)

Supplementary file70 (PNG 43 KB)

Supplementary file71 (PNG 34 KB)

Supplementary file72 (PNG 45 KB)

Supplementary file73 (PNG 46 KB)

Supplementary file74 (PNG 42 KB)

Supplementary file75 (PNG 43 KB)

Supplementary file76 (PNG 41 KB)

Supplementary file77 (PNG 41 KB)

Supplementary file78 (PNG 42 KB)

Supplementary file79 (PNG 43 KB)

Supplementary file80 (PNG 51 KB)

Supplementary file81 (PNG 41 KB)

Supplementary file82 (PNG 36 KB)

Supplementary file83 (PNG 46 KB)

Supplementary file84 (PNG 32 KB)

Supplementary file85 (PNG 34 KB)

Supplementary file86 (PNG 44 KB)

Supplementary file87 (PNG 41 KB)

## Data Availability

The datasets used and/or analyzed during the current study are available from the corresponding author on reasonable request.

## Abbreviations

DPSB: Double-pulley suture-bridge
SR: Single-row
VAS: Visual analog scale
ASES: American Shoulder and Elbow Surgeons
UCLA: University of California, Los Angeles
ROM: Range of motion
MRI: Magnetic resonance imaging
DR: Double-row
