# Supplementary figures and images for: Comparison of Implant Type, Number and Cost, Suture Number, Surgical Time, Clinical Outcomes of Arthroscopic Double-Pulley Suture-Bridge and Single-Row in Repair Supraspinatus Tendon Tears: A Novel Suture-Bridge Technique
Source: Indian J Orthop. 2025 Dec 25;60(7):1828–39. doi: 10.1007/s43465-025-01645-6 (PMC13385317; doi:10.1007/s43465-025-01645-6)

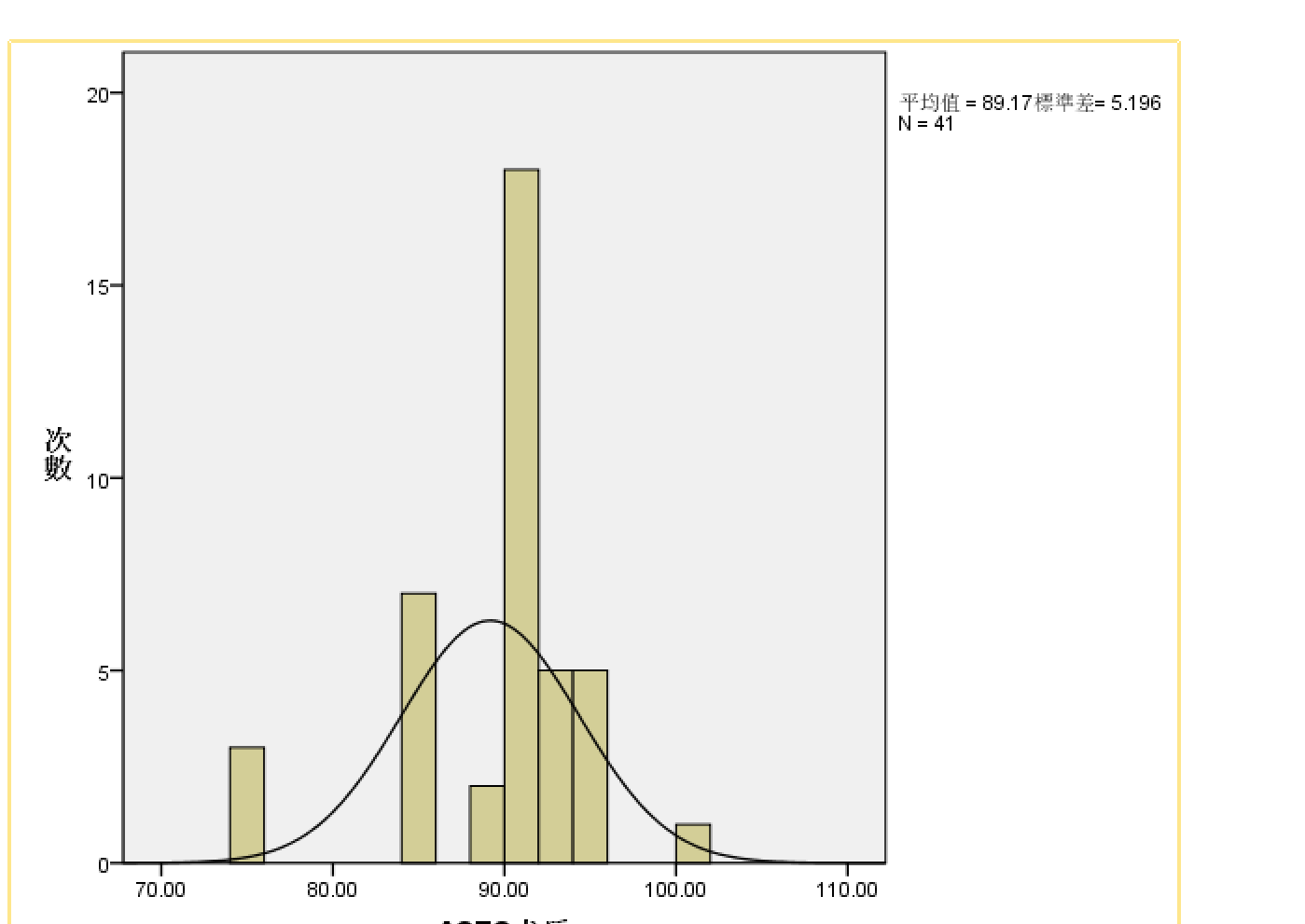

Supplement: Supplementary file 1 — Supplementary file1 (PNG 43 KB) [file 43465_2025_1645_MOESM1_ESM.png]

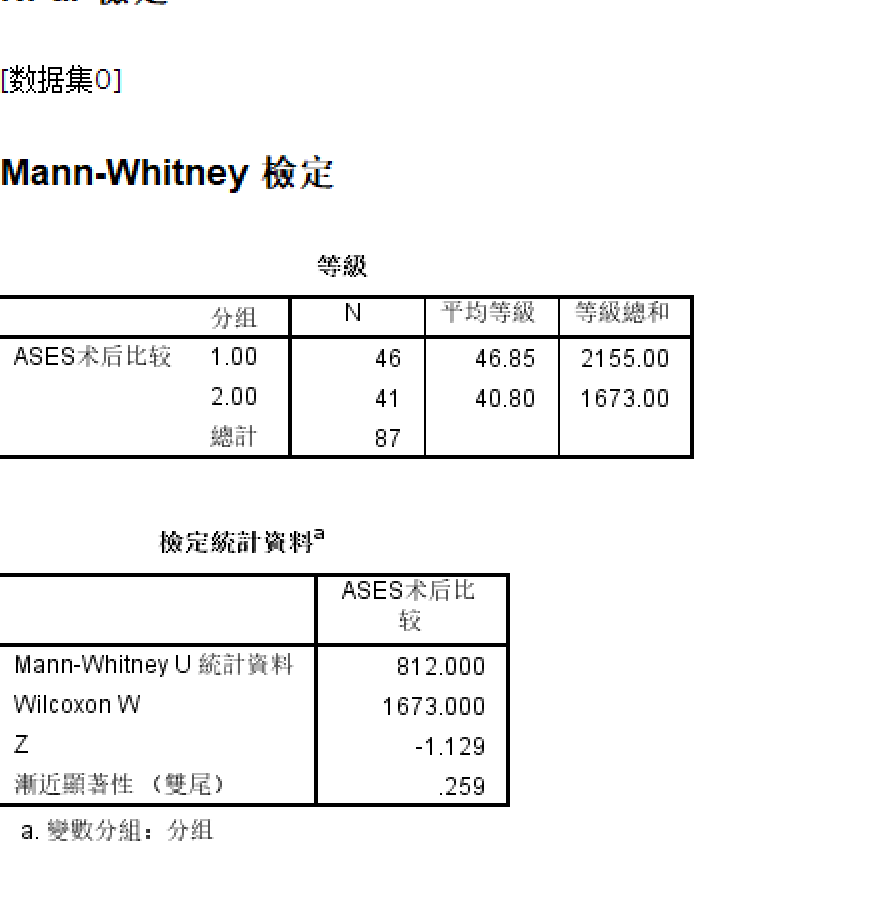

Supplement: Supplementary file 2 — Supplementary file2 (PNG 46 KB) [file 43465_2025_1645_MOESM2_ESM.png]

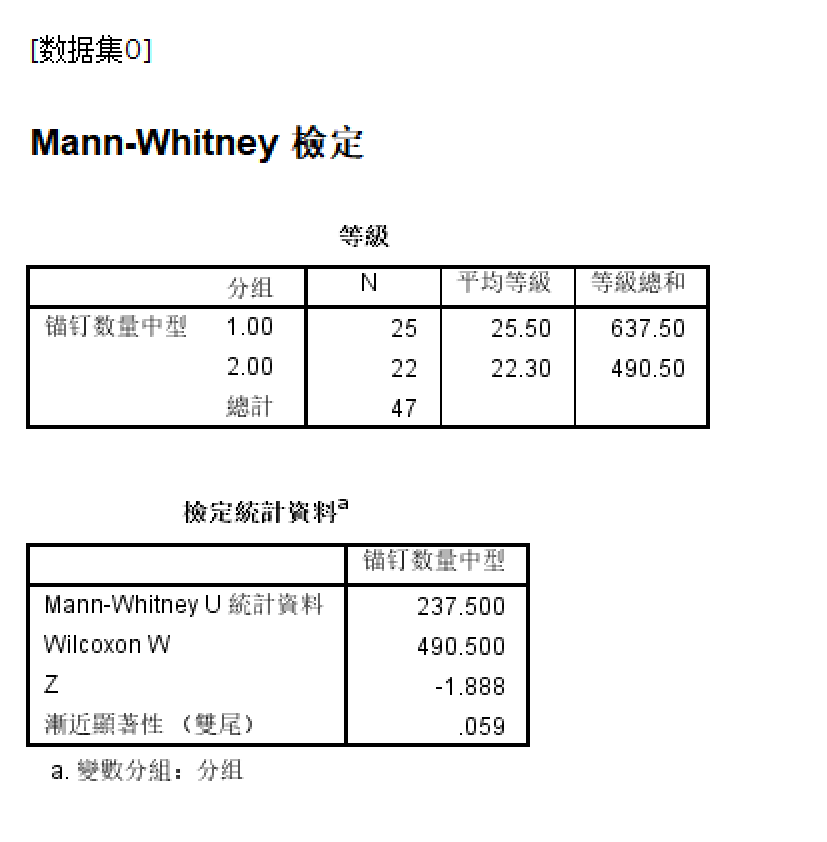

Supplement: Supplementary file 3 — Supplementary file3 (PNG 39 KB) [file 43465_2025_1645_MOESM3_ESM.png]

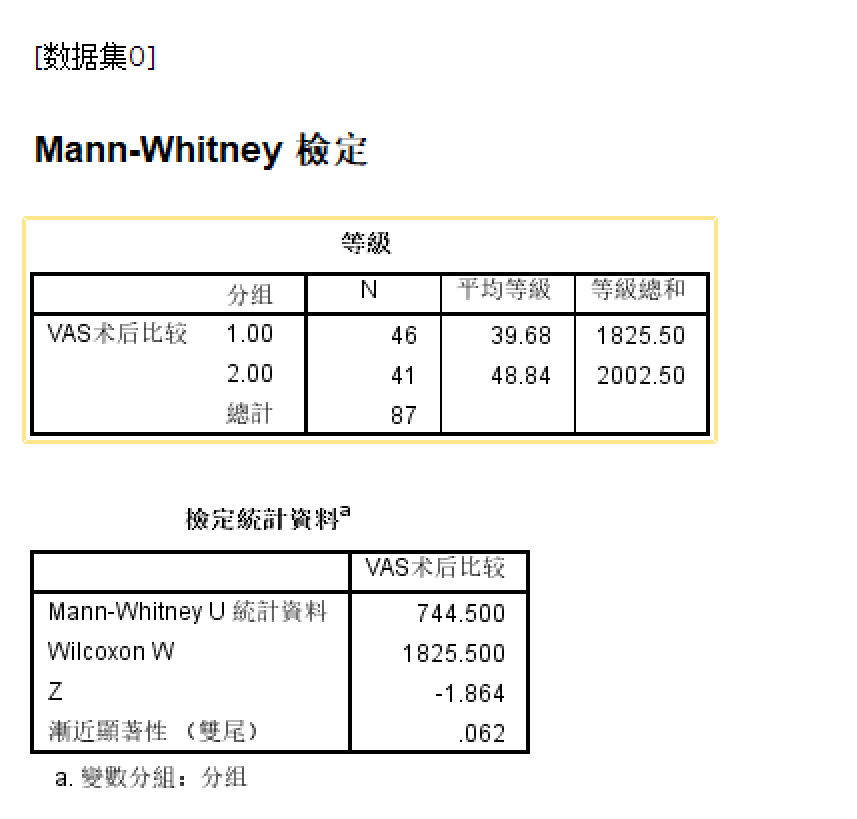

Supplement: Supplementary file 4 — Supplementary file4 (PNG 33 KB) [file 43465_2025_1645_MOESM4_ESM.png]

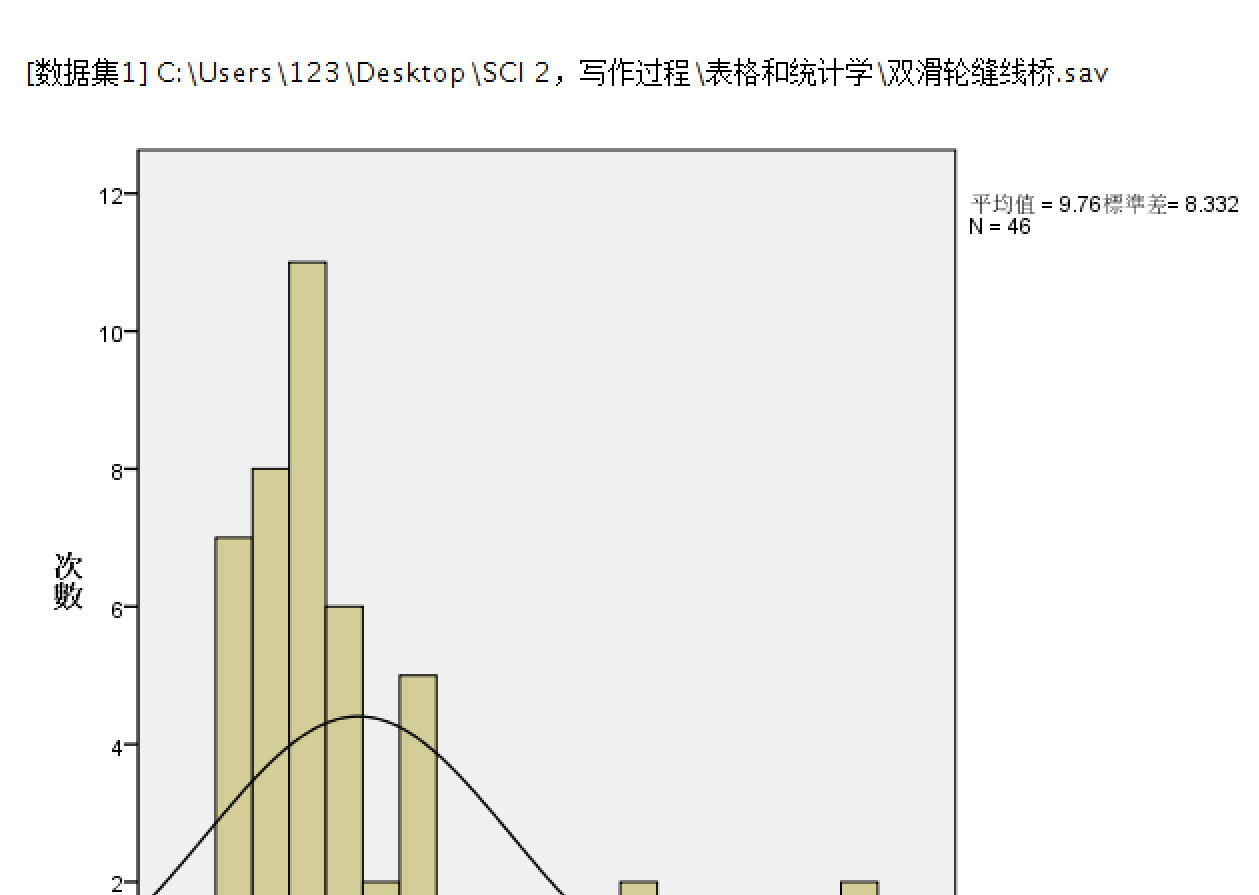

Supplement: Supplementary file 5 — Supplementary file5 (PNG 30 KB) [file 43465_2025_1645_MOESM5_ESM.png]

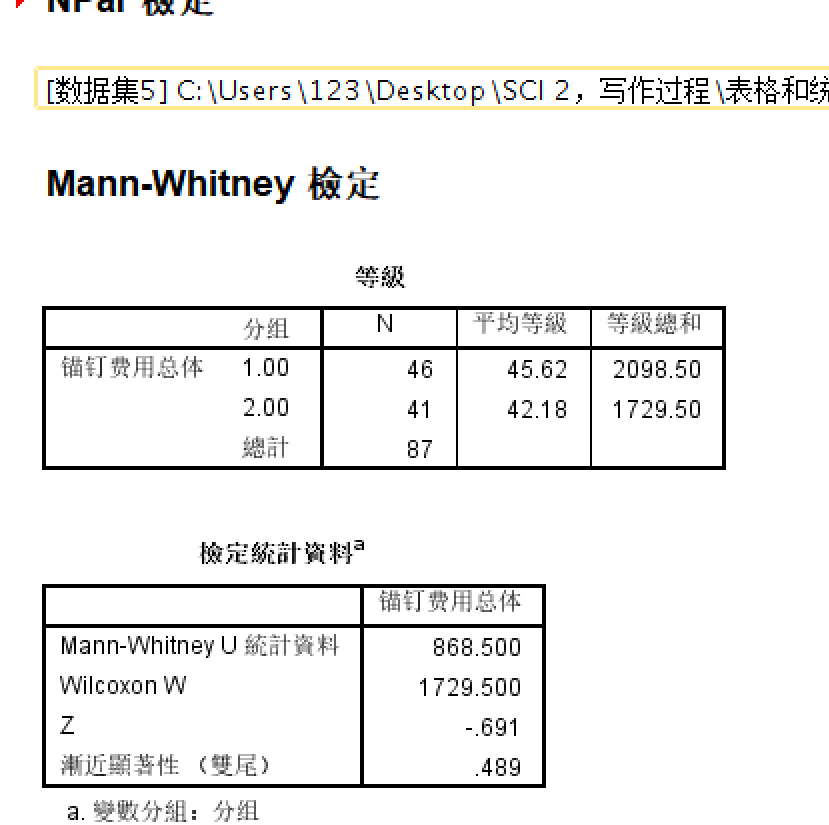

Supplement: Supplementary file 6 — Supplementary file6 (PNG 34 KB) [file 43465_2025_1645_MOESM6_ESM.png]

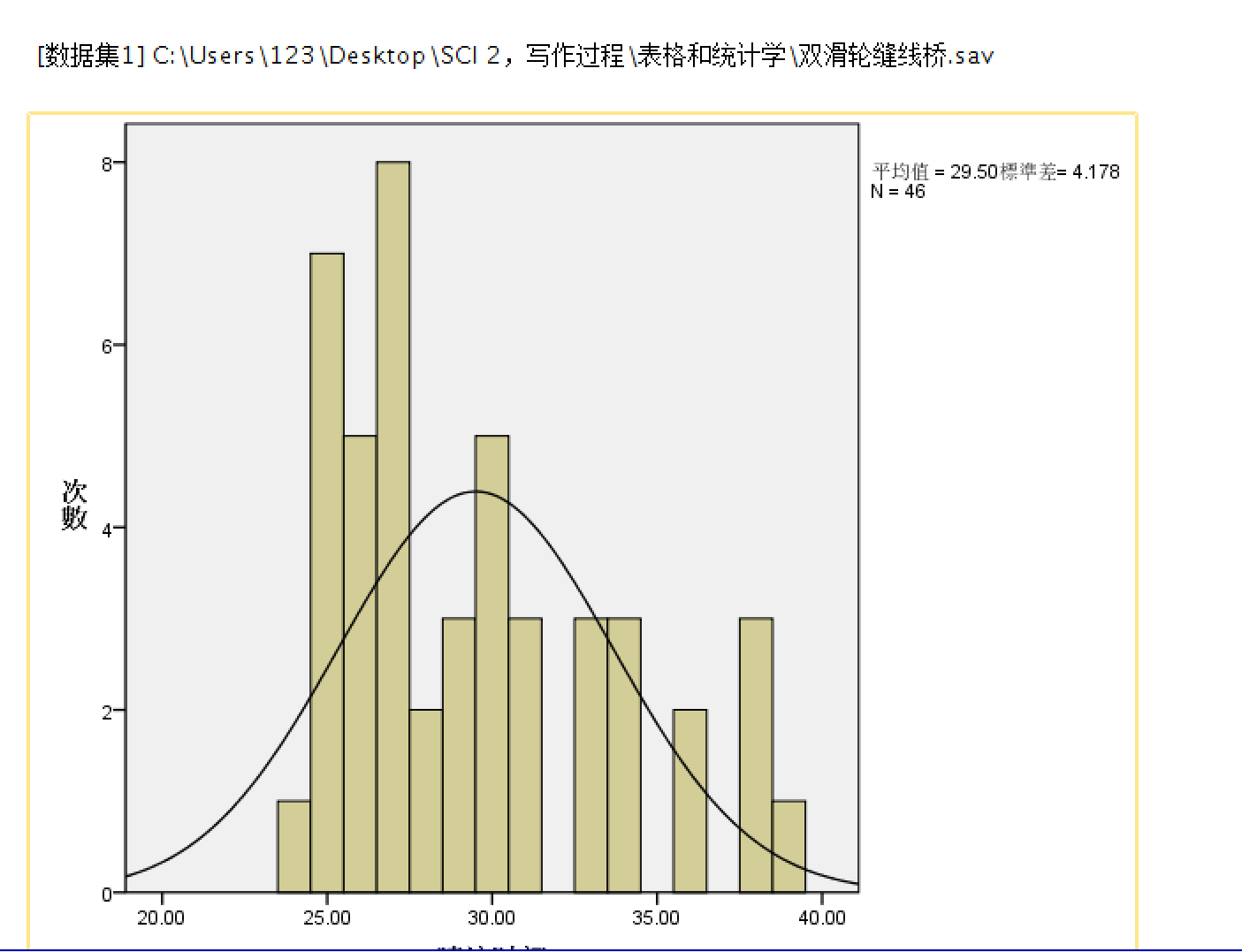

Supplement: Supplementary file 7 — Supplementary file7 (PNG 31 KB) [file 43465_2025_1645_MOESM7_ESM.png]

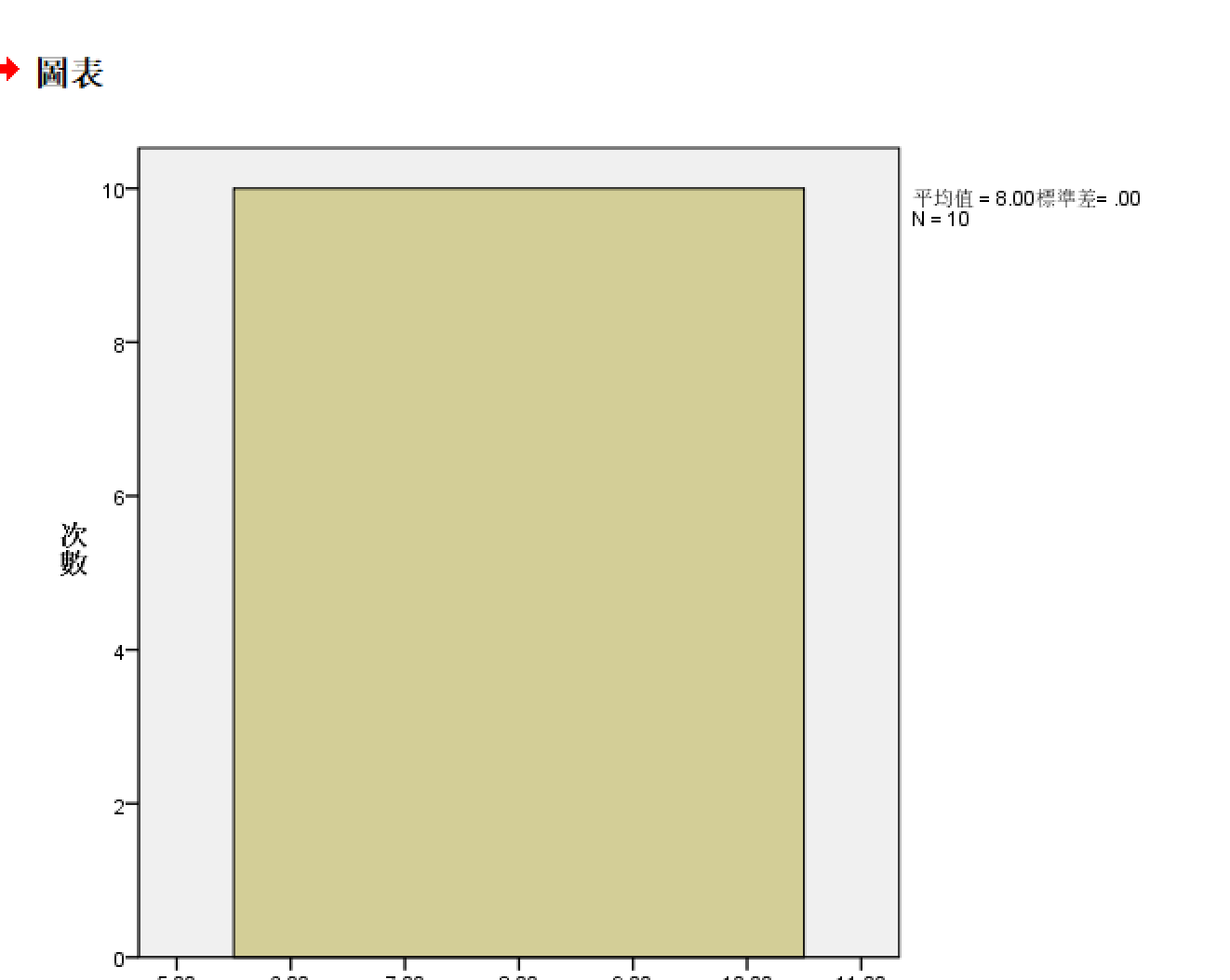

Supplement: Supplementary file 8 — Supplementary file8 (PNG 30 KB) [file 43465_2025_1645_MOESM8_ESM.png]

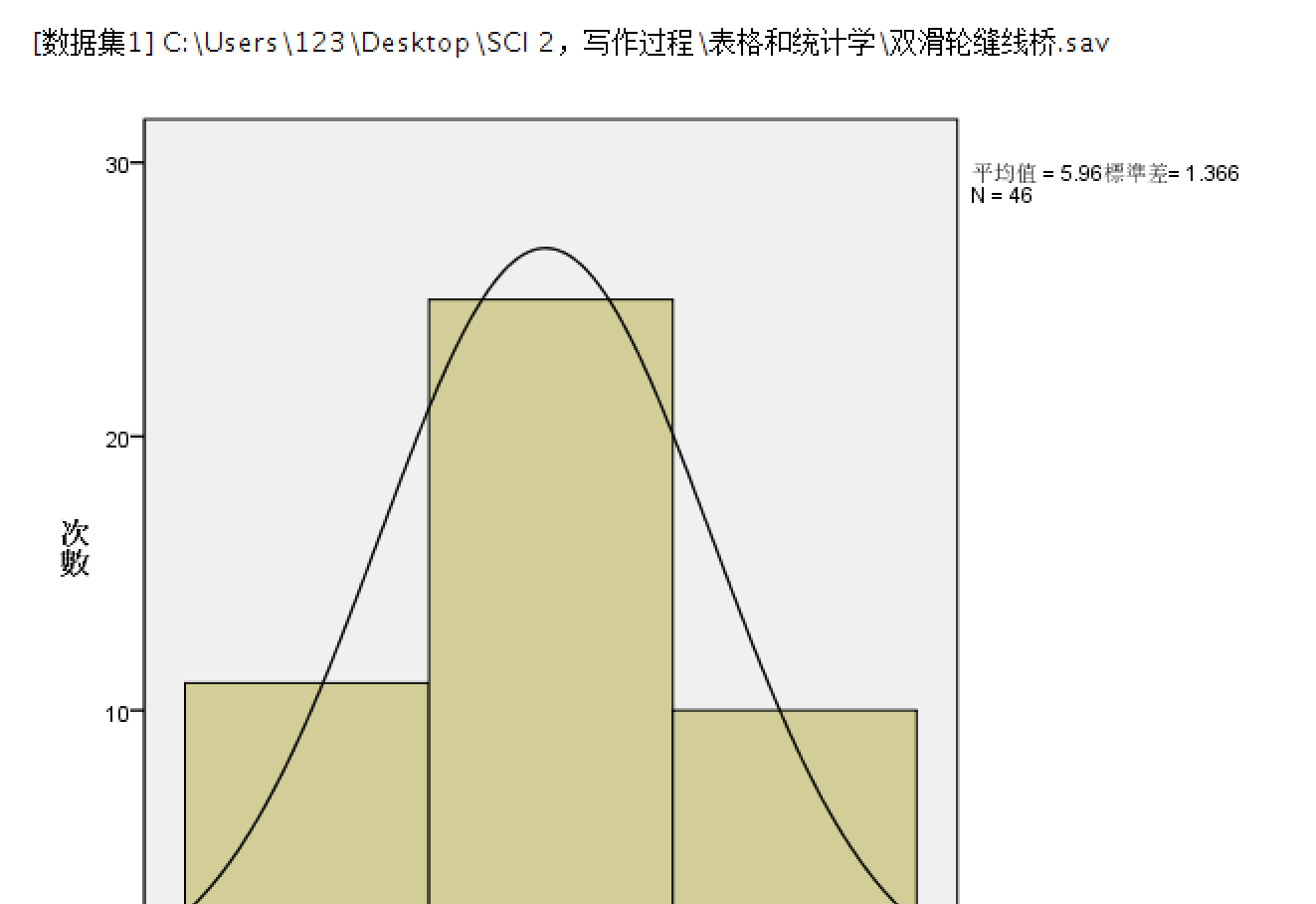

Supplement: Supplementary file 9 — Supplementary file9 (PNG 30 KB) [file 43465_2025_1645_MOESM9_ESM.png]

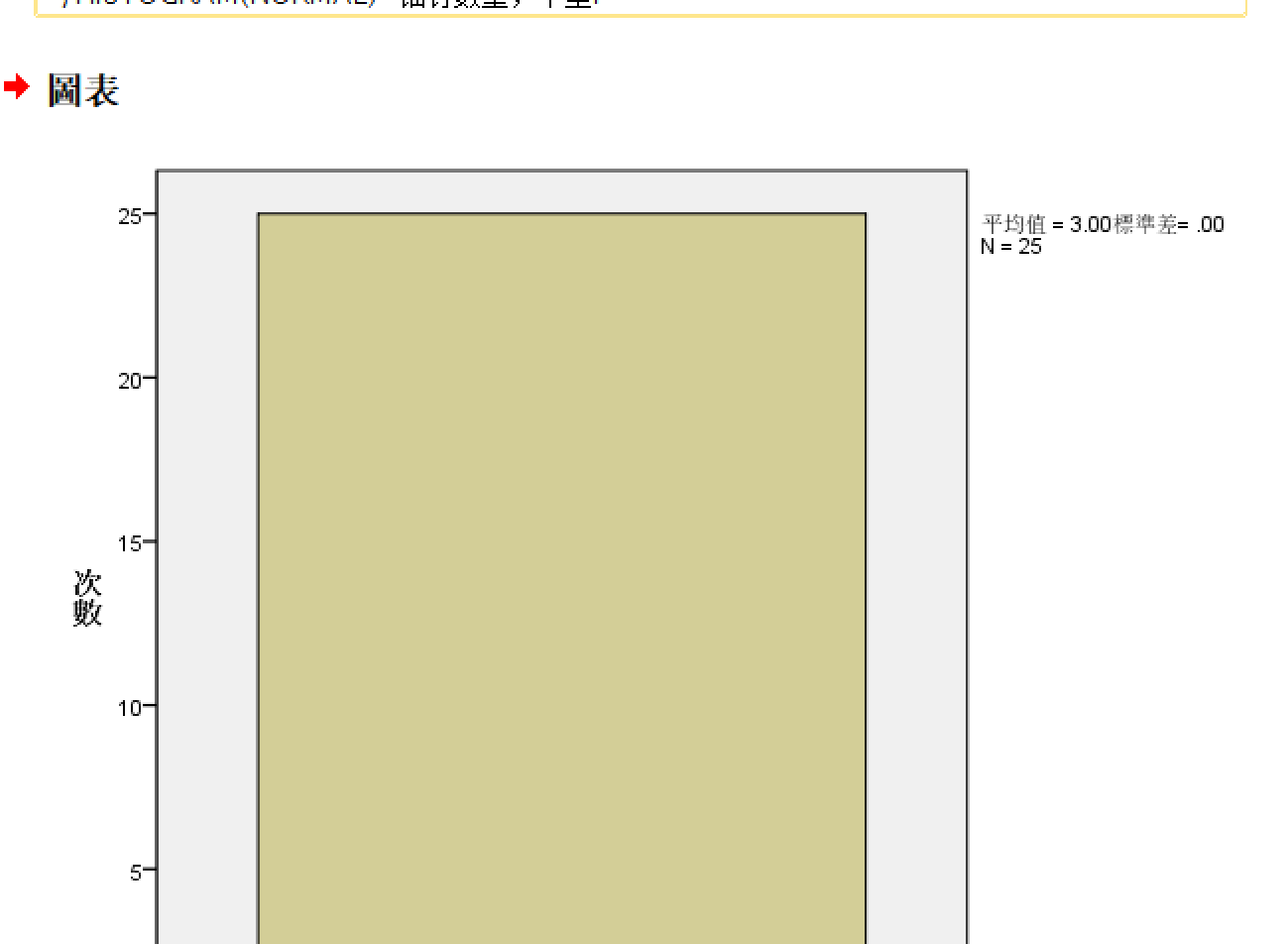

Supplement: Supplementary file 10 — Supplementary file10 (PNG 37 KB) [file 43465_2025_1645_MOESM10_ESM.png]

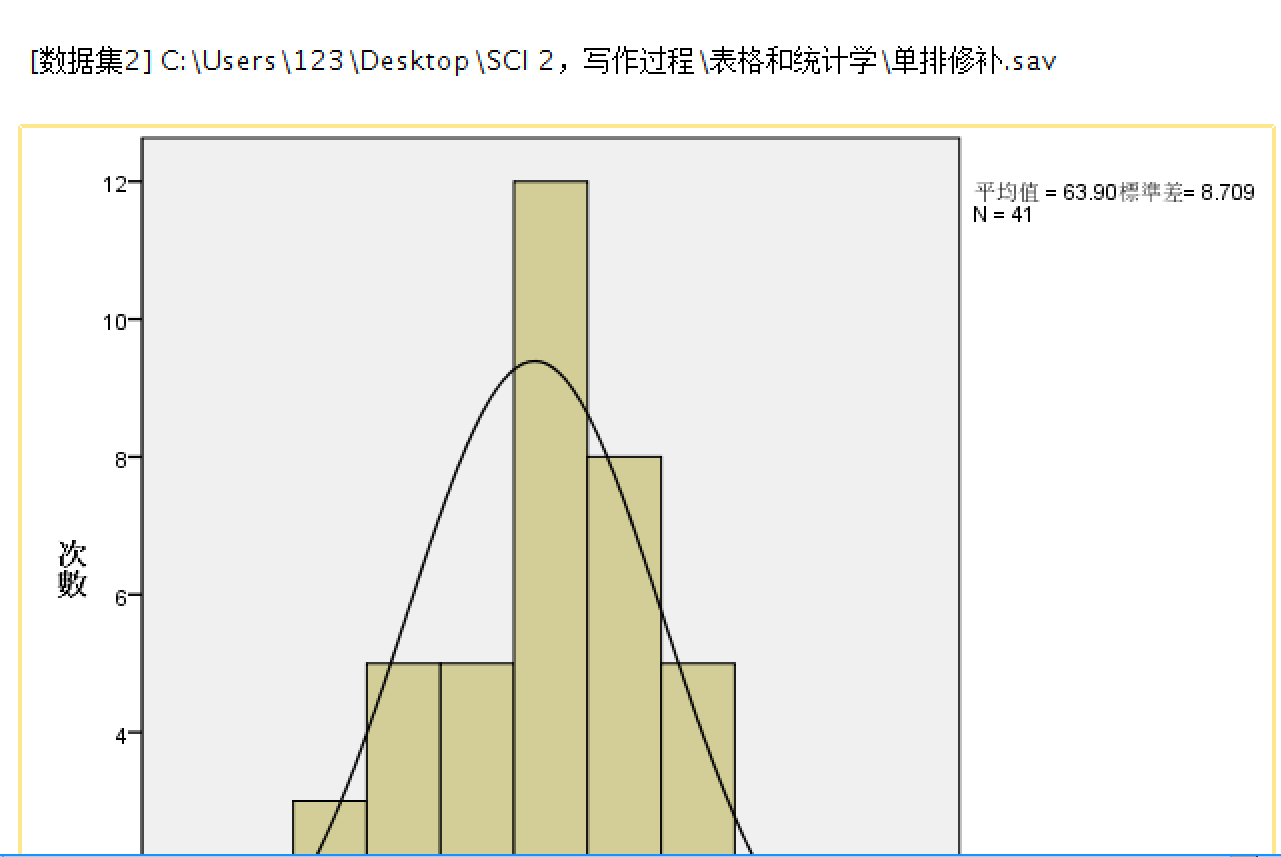

Supplement: Supplementary file 11 — Supplementary file11 (PNG 33 KB) [file 43465_2025_1645_MOESM11_ESM.png]

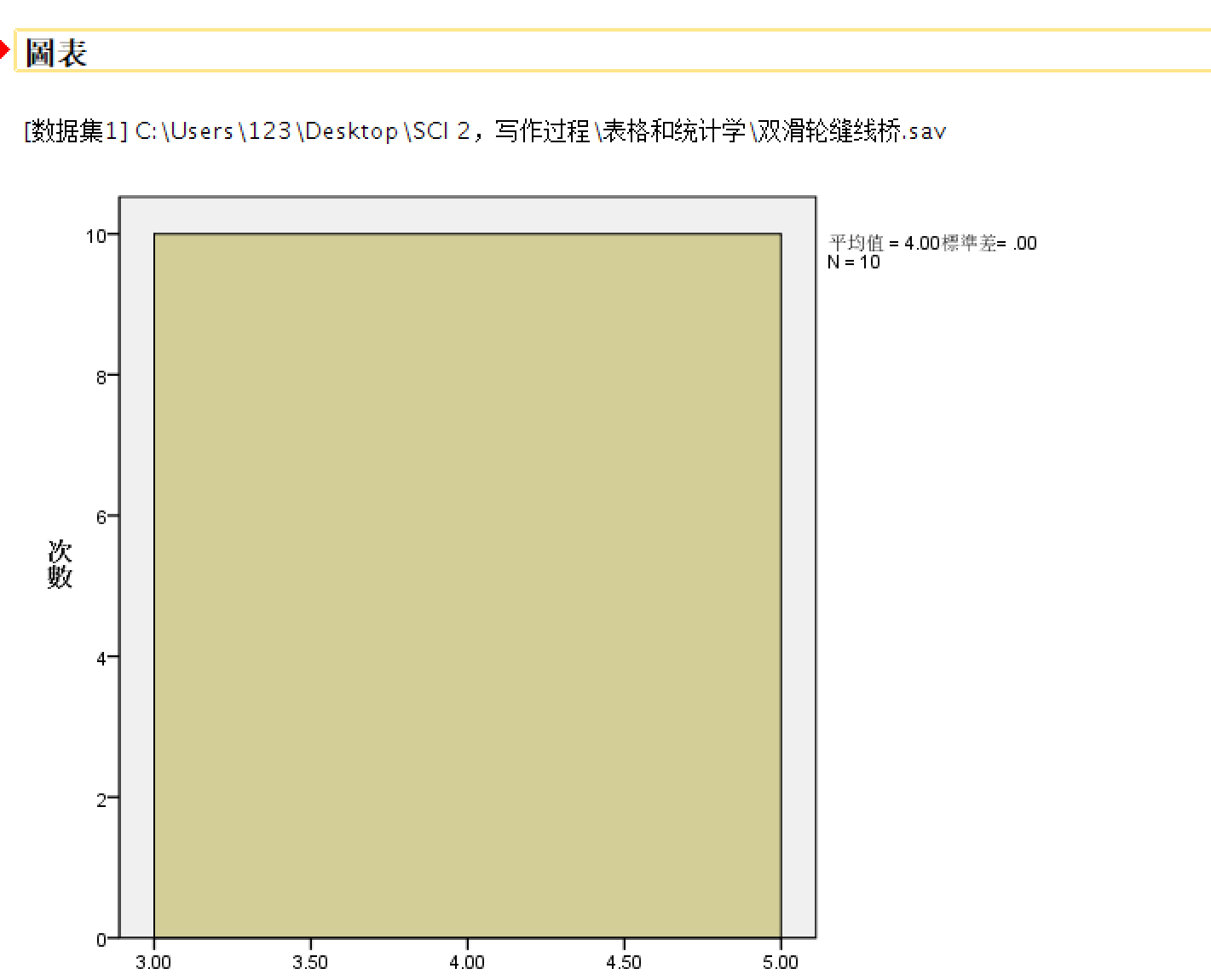

Supplement: Supplementary file 12 — Supplementary file12 (PNG 34 KB) [file 43465_2025_1645_MOESM12_ESM.png]

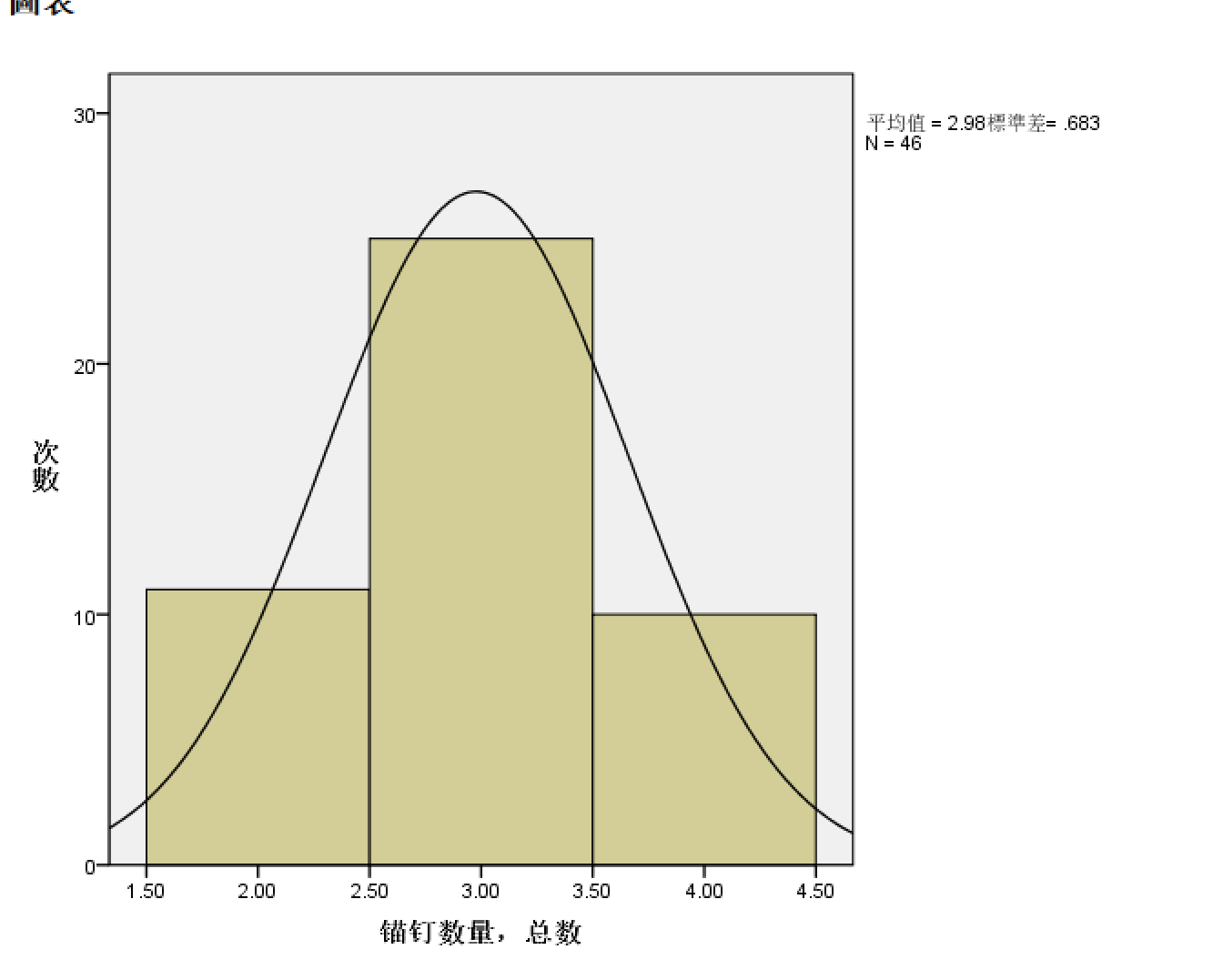

Supplement: Supplementary file 13 — Supplementary file13 (PNG 32 KB) [file 43465_2025_1645_MOESM13_ESM.png]

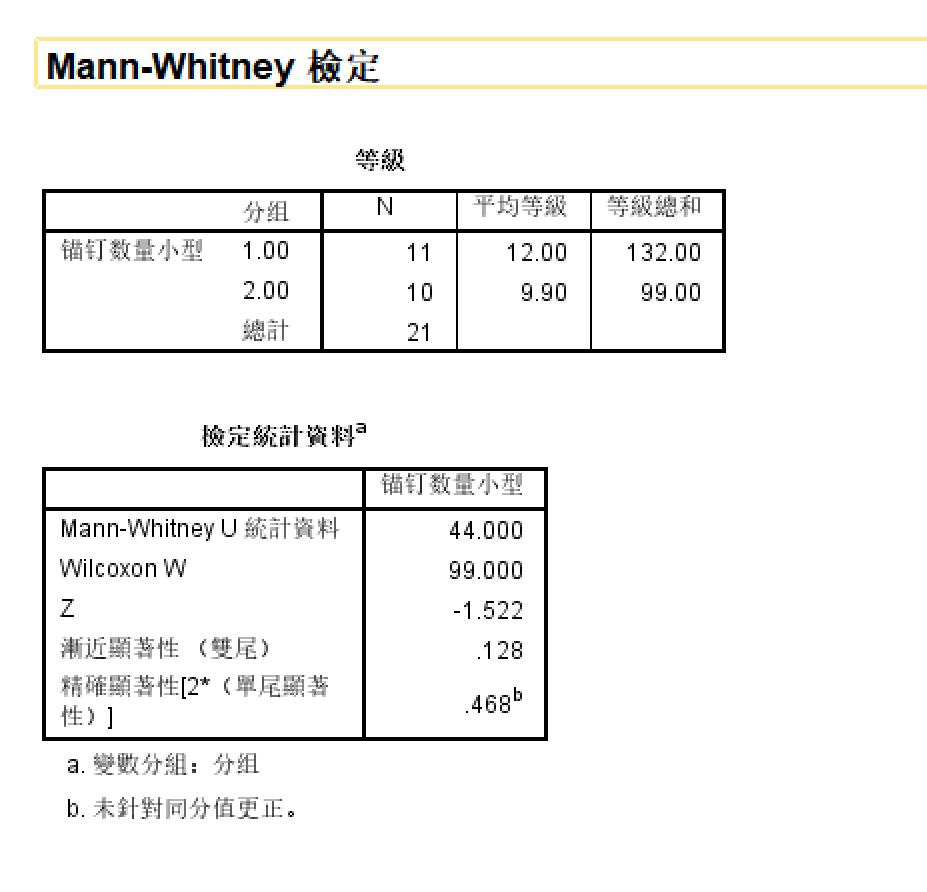

Supplement: Supplementary file 14 — Supplementary file14 (PNG 47 KB) [file 43465_2025_1645_MOESM14_ESM.png]

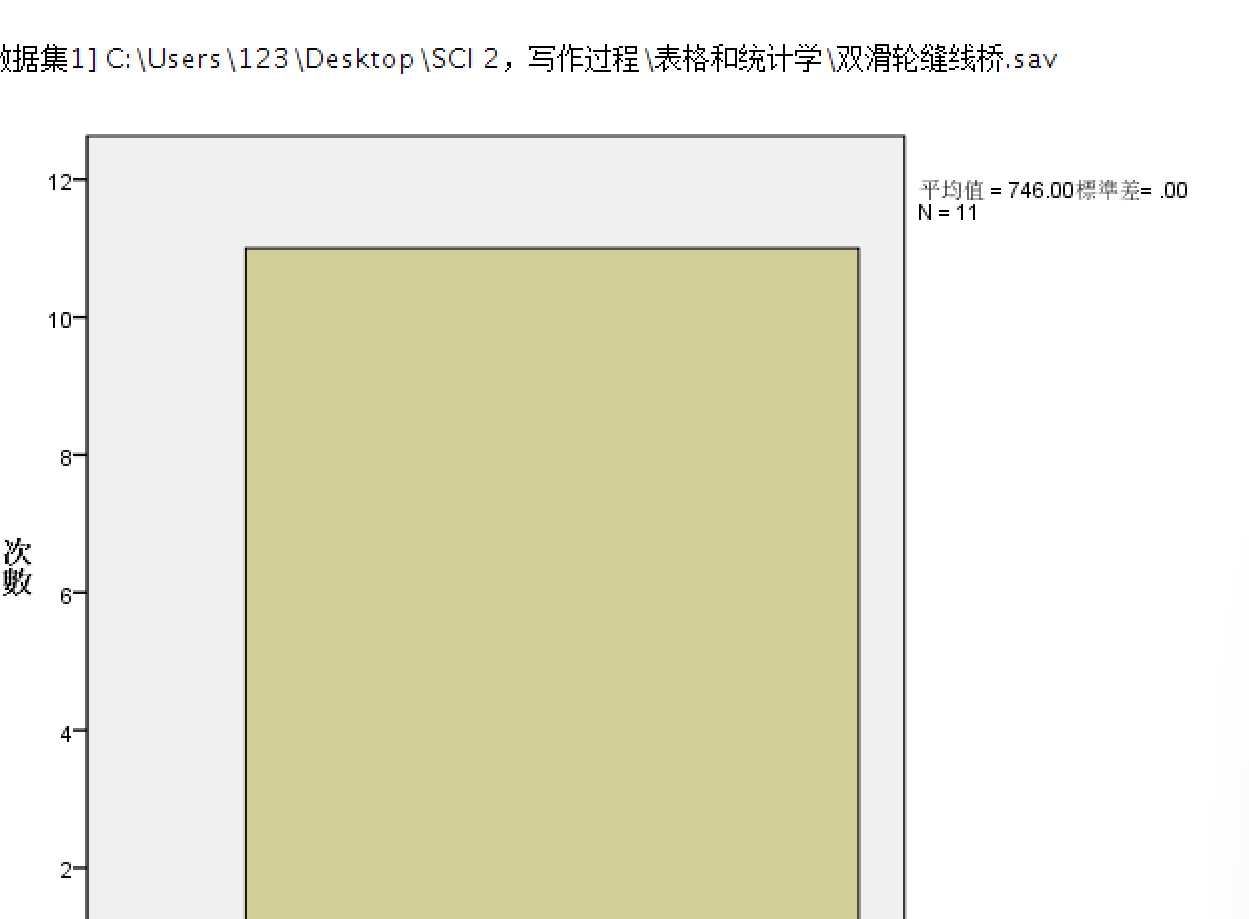

Supplement: Supplementary file 15 — Supplementary file15 (PNG 31 KB) [file 43465_2025_1645_MOESM15_ESM.png]

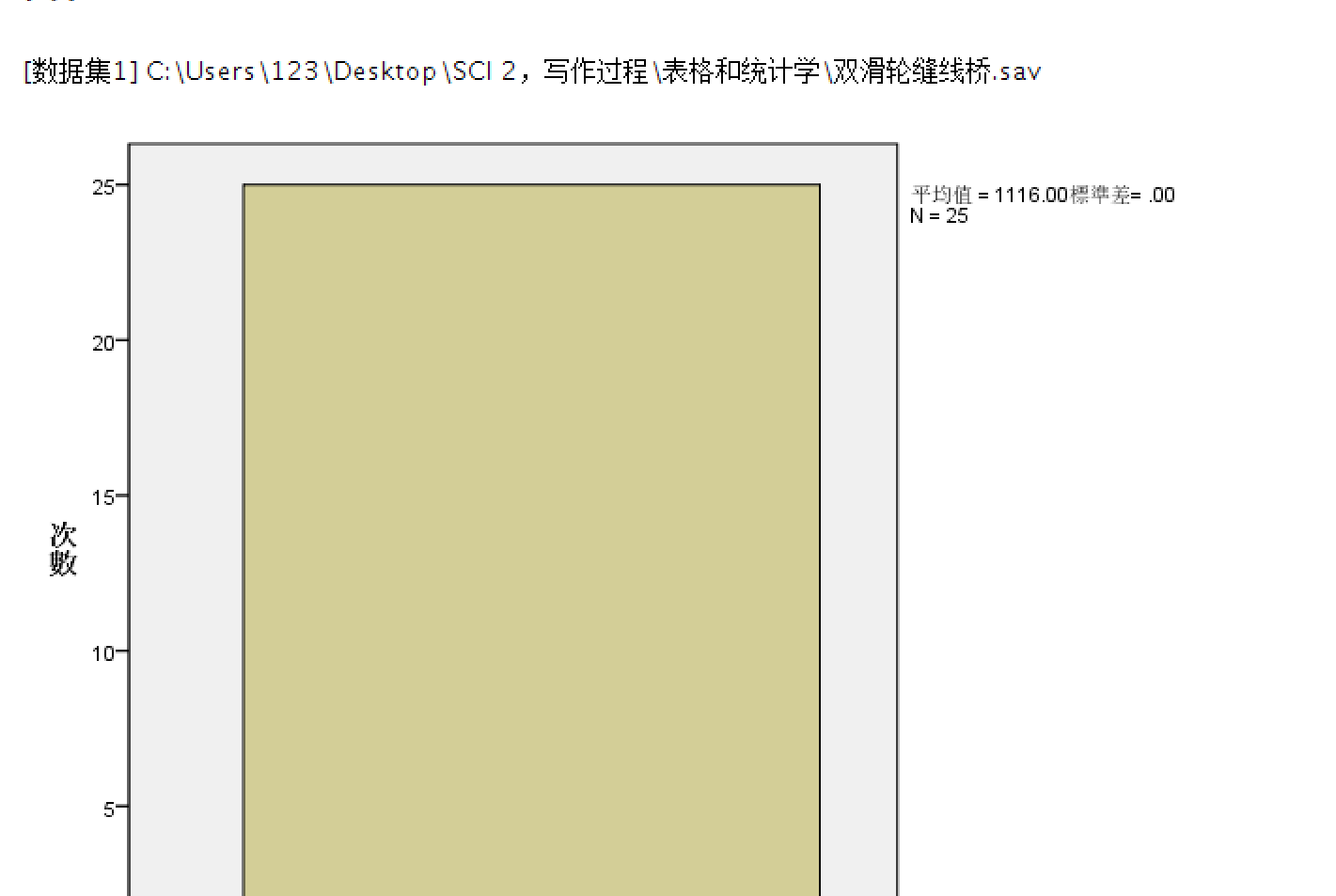

Supplement: Supplementary file 16 — Supplementary file16 (PNG 30 KB) [file 43465_2025_1645_MOESM16_ESM.png]

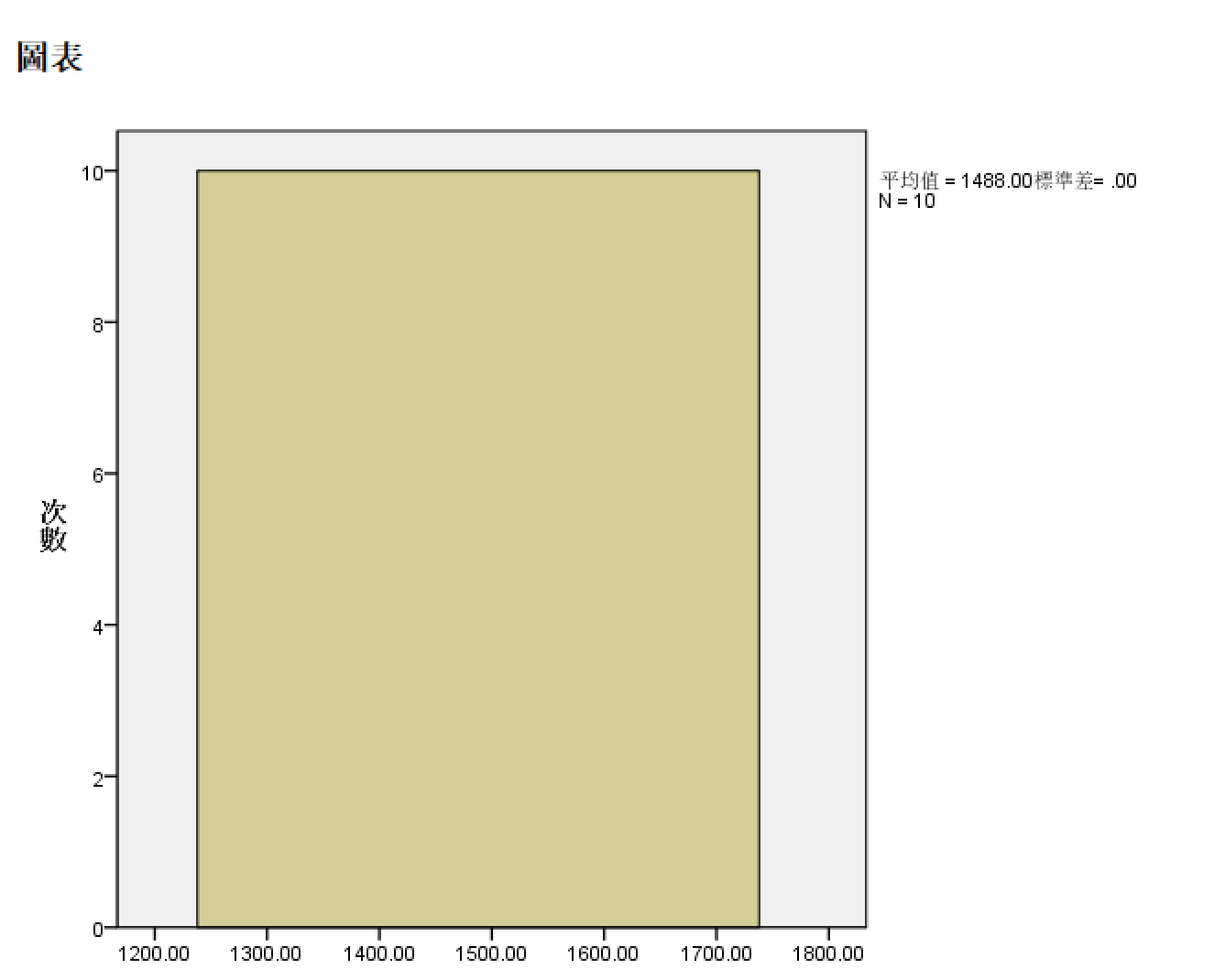

Supplement: Supplementary file 17 — Supplementary file17 (PNG 42 KB) [file 43465_2025_1645_MOESM17_ESM.png]

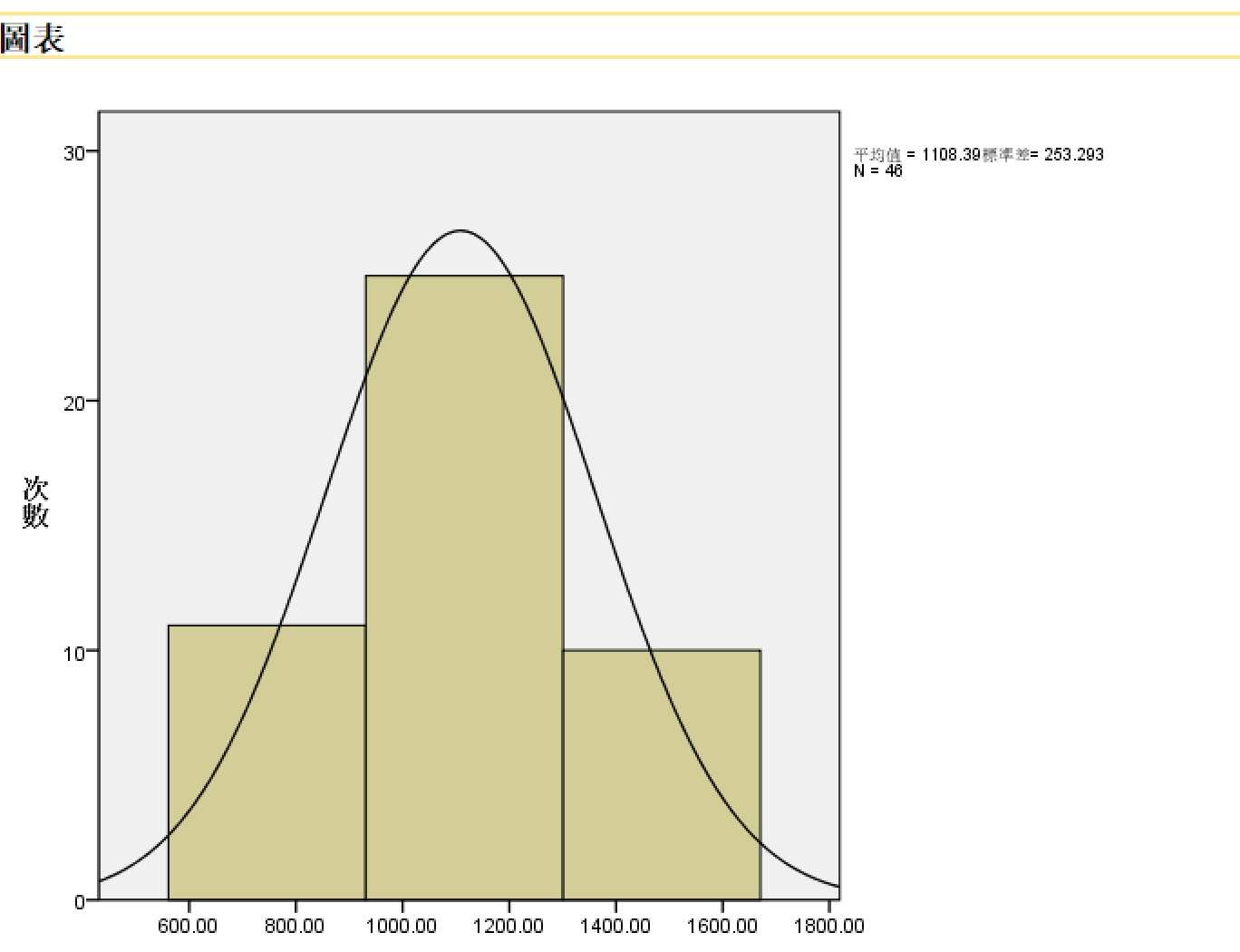

Supplement: Supplementary file 18 — Supplementary file18 (PNG 38 KB) [file 43465_2025_1645_MOESM18_ESM.png]

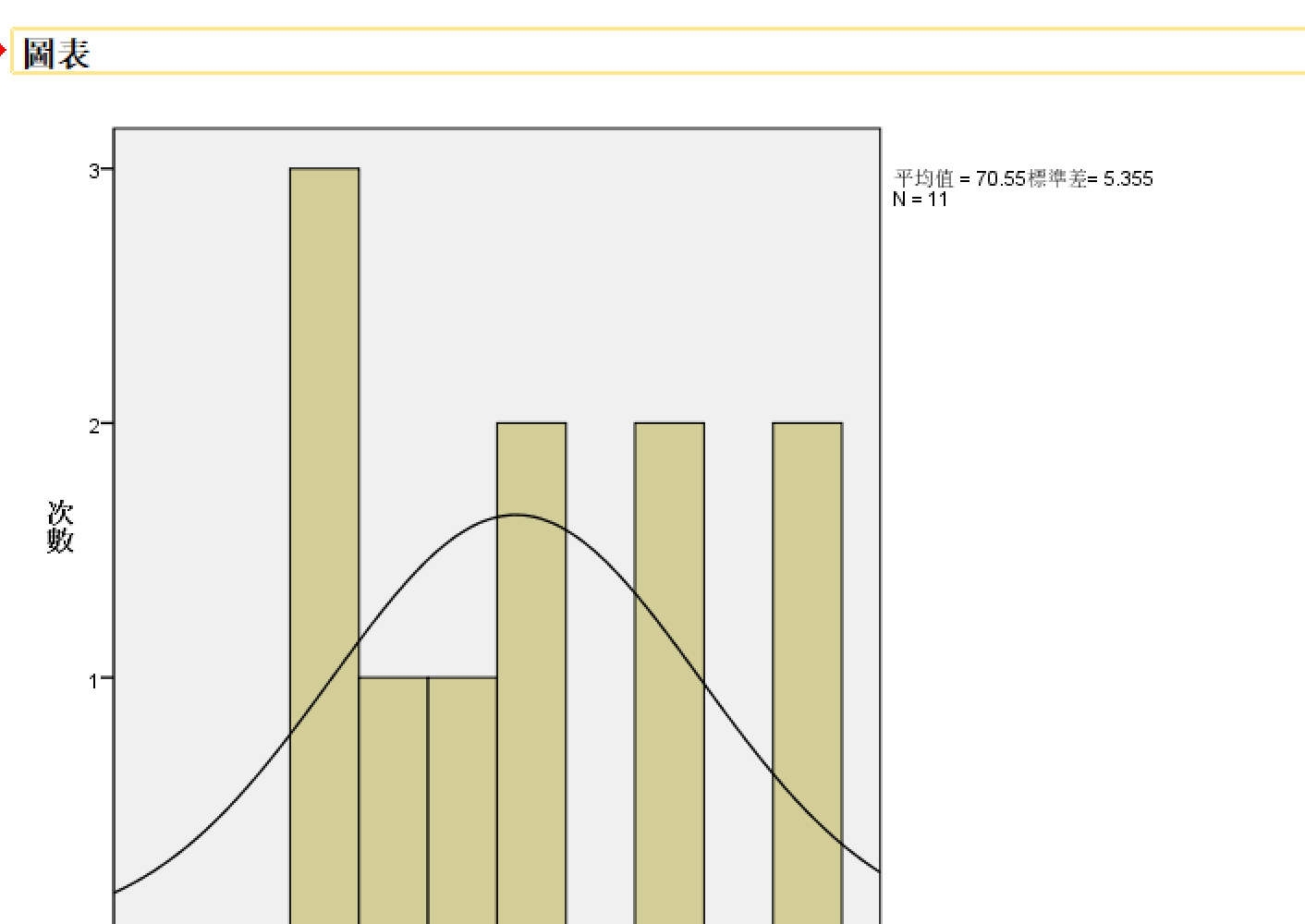

Supplement: Supplementary file 19 — Supplementary file19 (PNG 33 KB) [file 43465_2025_1645_MOESM19_ESM.png]

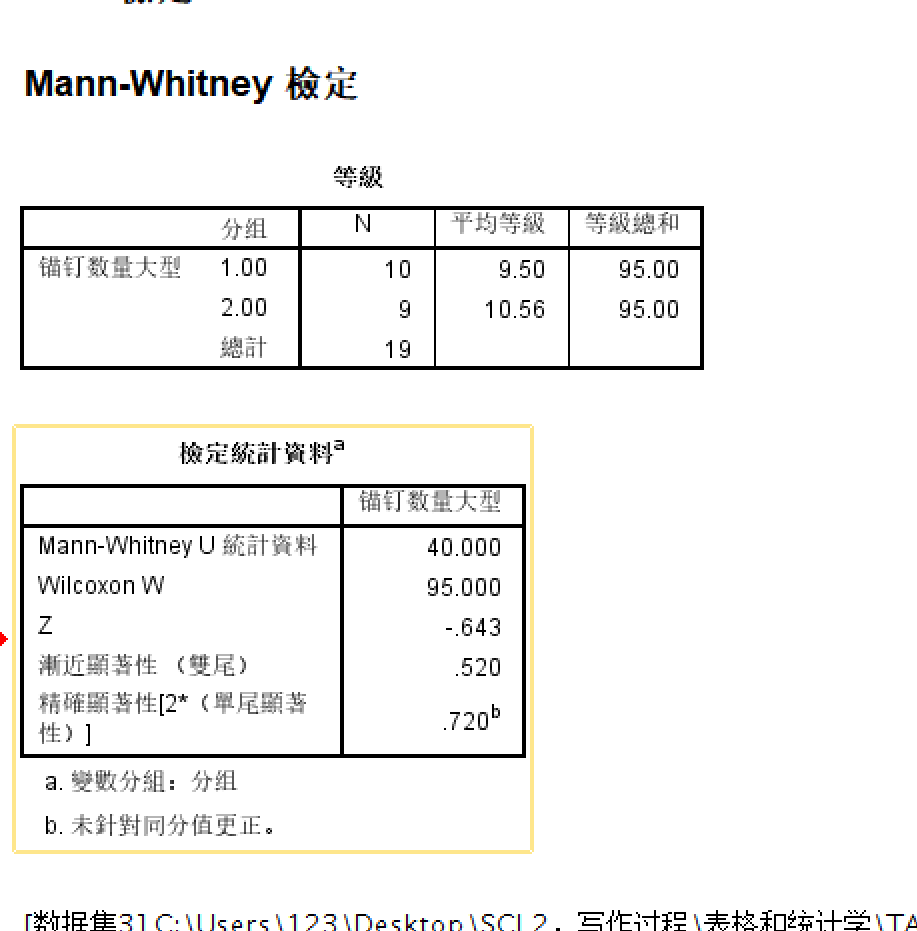

Supplement: Supplementary file 20 — Supplementary file20 (PNG 39 KB) [file 43465_2025_1645_MOESM20_ESM.png]

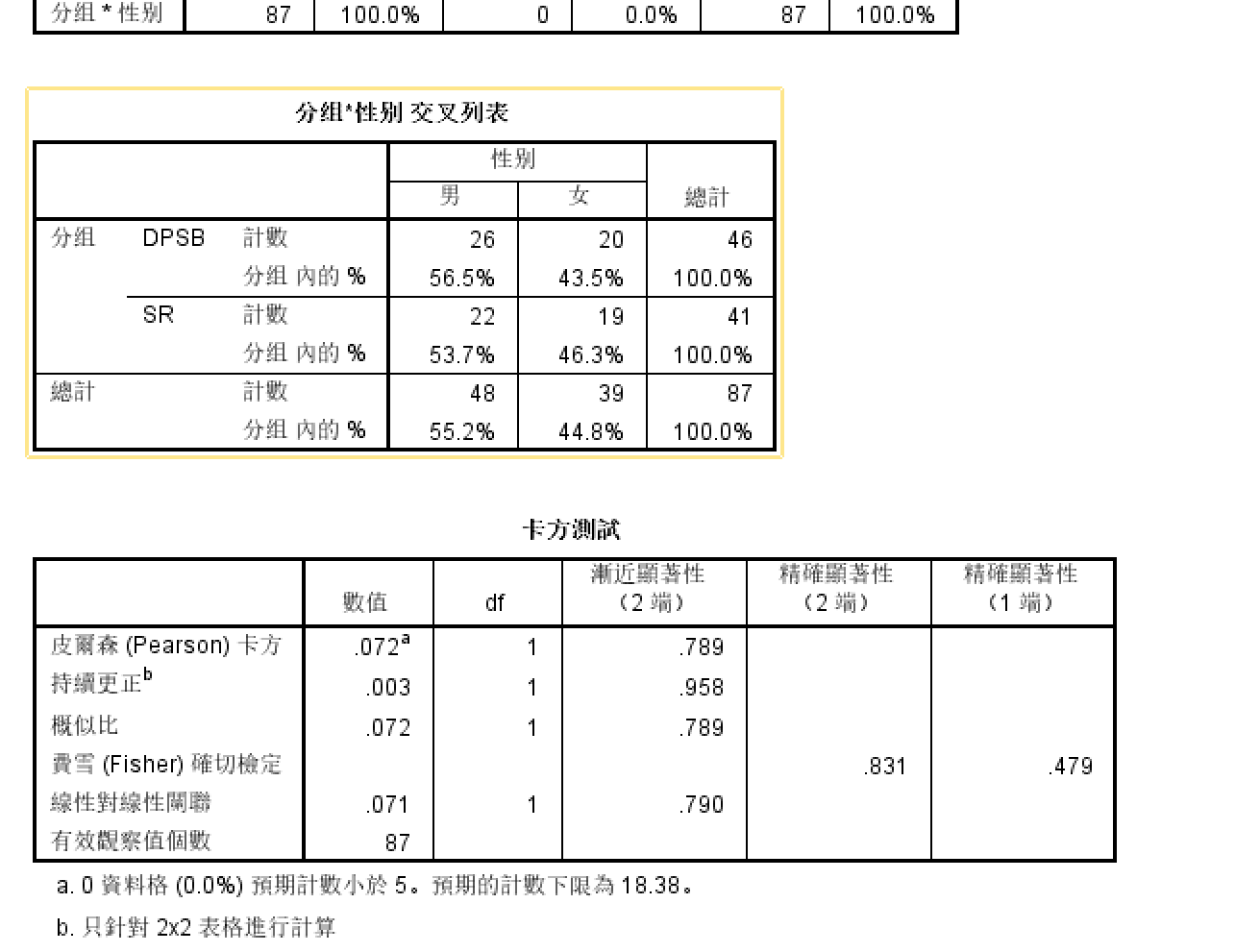

Supplement: Supplementary file 21 — Supplementary file21 (PNG 31 KB) [file 43465_2025_1645_MOESM21_ESM.png]

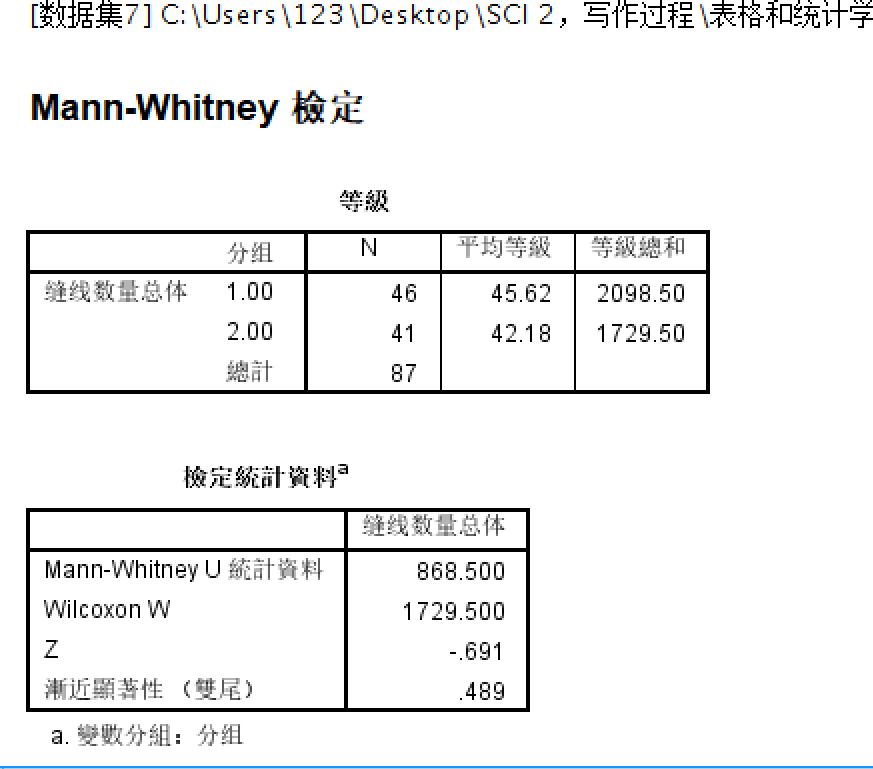

Supplement: Supplementary file 22 — Supplementary file22 (PNG 30 KB) [file 43465_2025_1645_MOESM22_ESM.png]

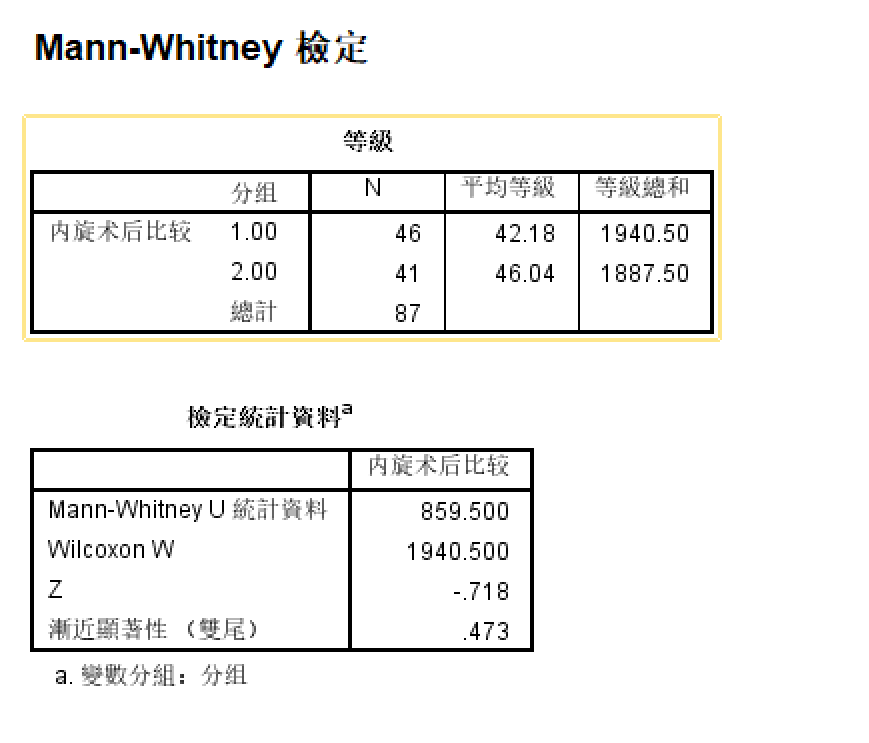

Supplement: Supplementary file 23 — Supplementary file23 (PNG 34 KB) [file 43465_2025_1645_MOESM23_ESM.png]

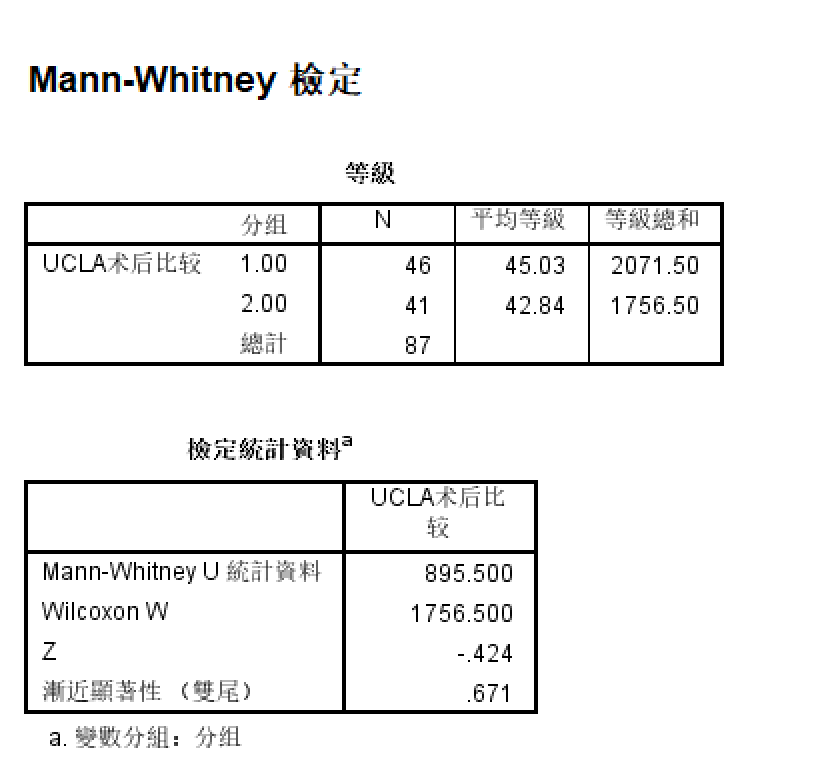

Supplement: Supplementary file 24 — Supplementary file24 (PNG 48 KB) [file 43465_2025_1645_MOESM24_ESM.png]

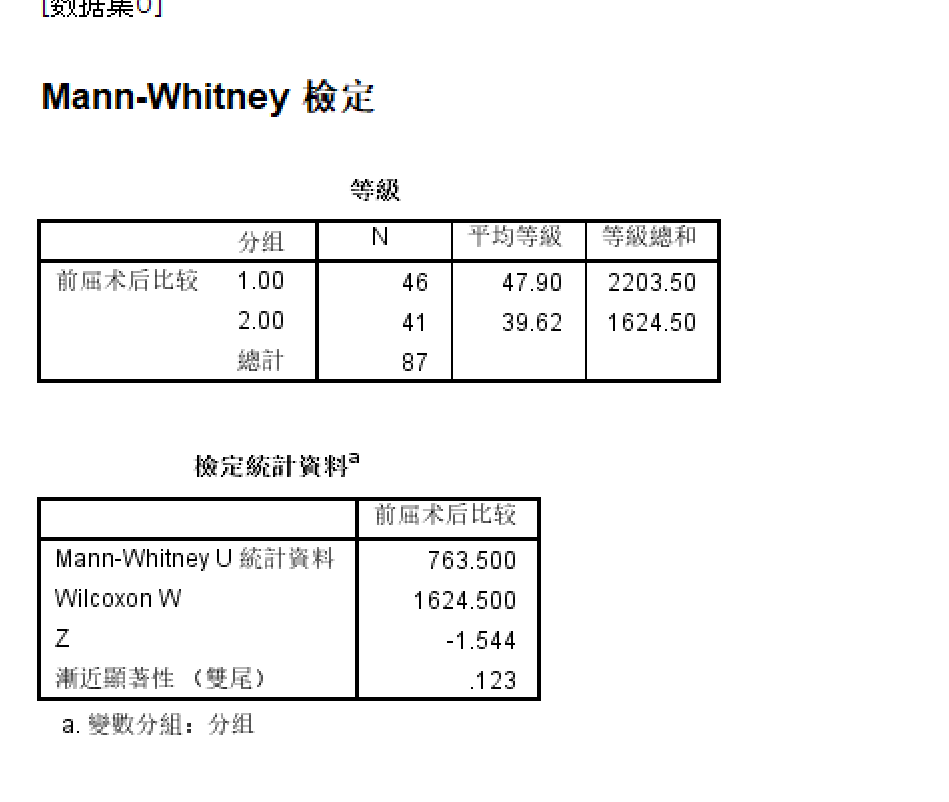

Supplement: Supplementary file 25 — Supplementary file25 (PNG 30 KB) [file 43465_2025_1645_MOESM25_ESM.png]

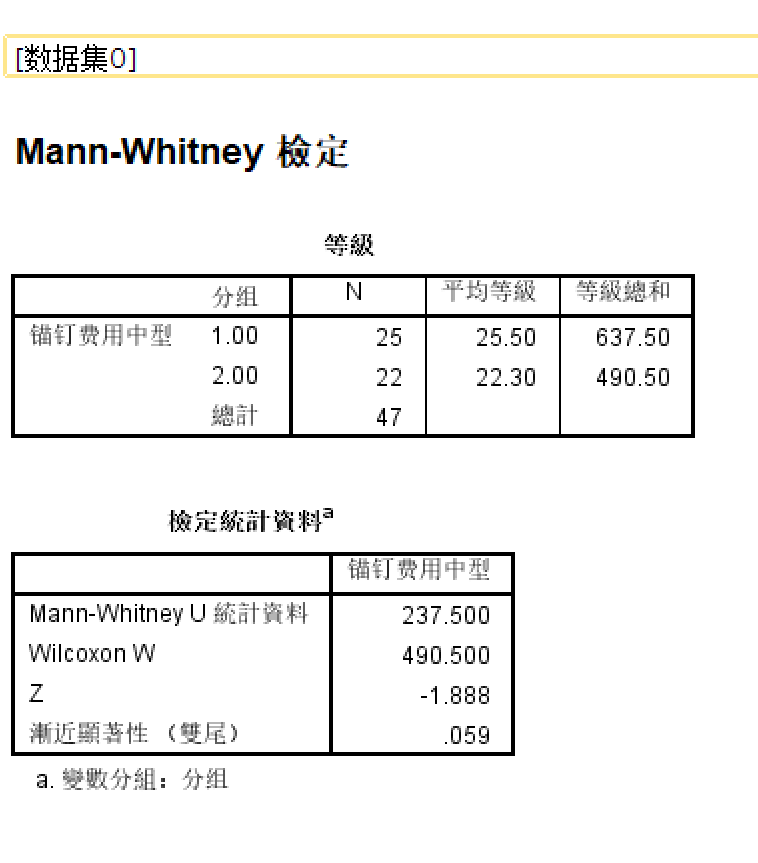

Supplement: Supplementary file 26 — Supplementary file26 (PNG 43 KB) [file 43465_2025_1645_MOESM26_ESM.png]

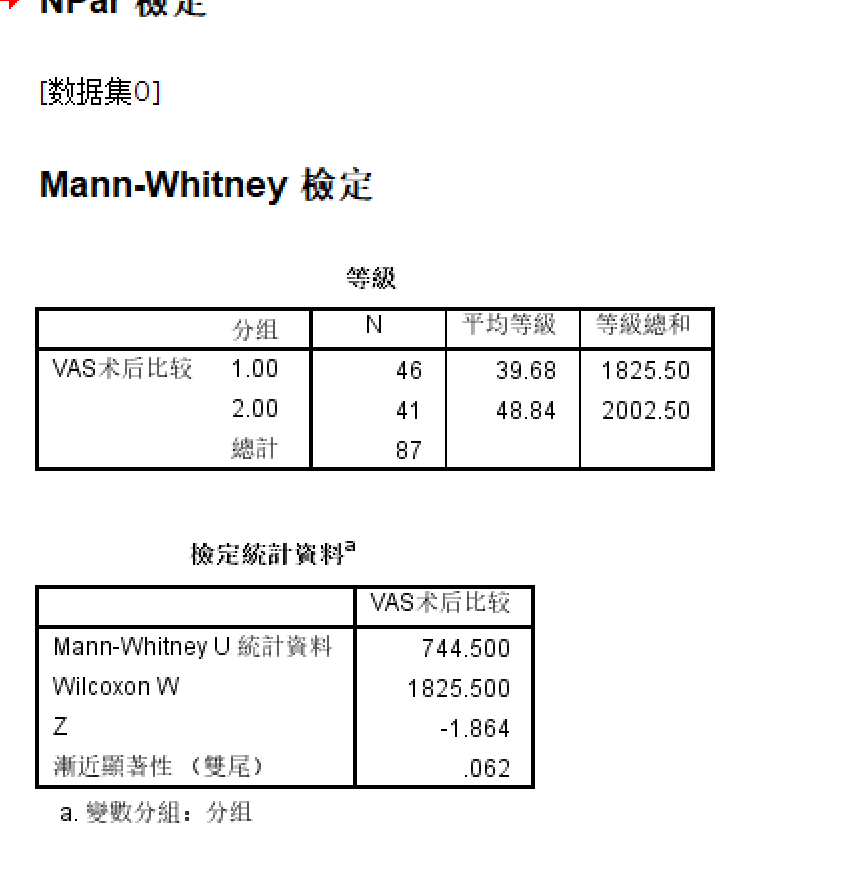

Supplement: Supplementary file 27 — Supplementary file27 (PNG 28 KB) [file 43465_2025_1645_MOESM27_ESM.png]

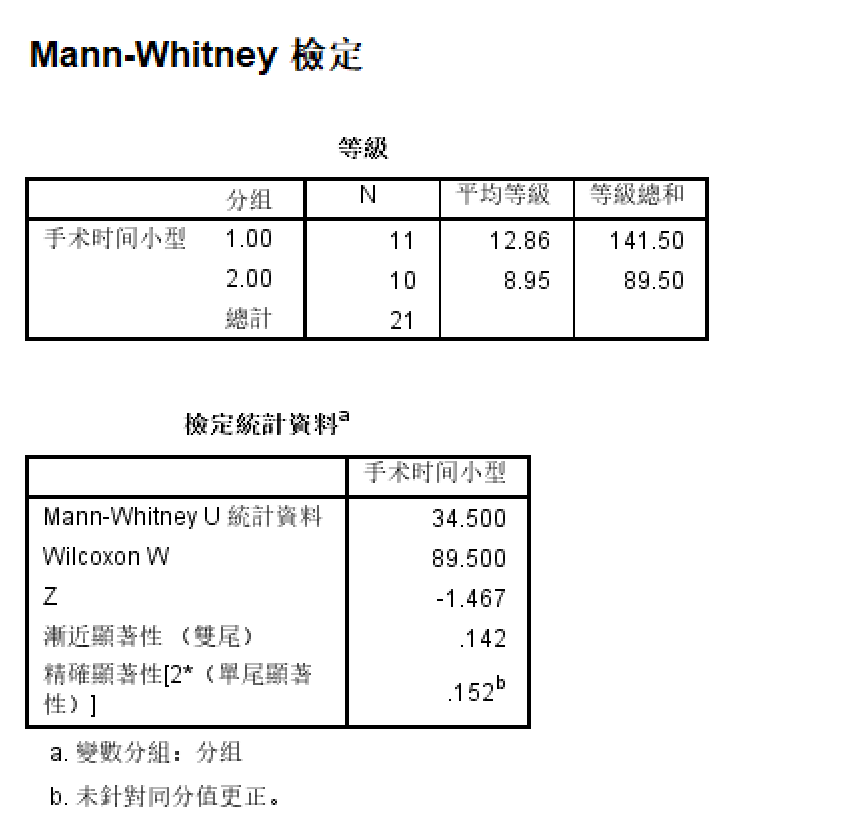

Supplement: Supplementary file 28 — Supplementary file28 (PNG 39 KB) [file 43465_2025_1645_MOESM28_ESM.png]

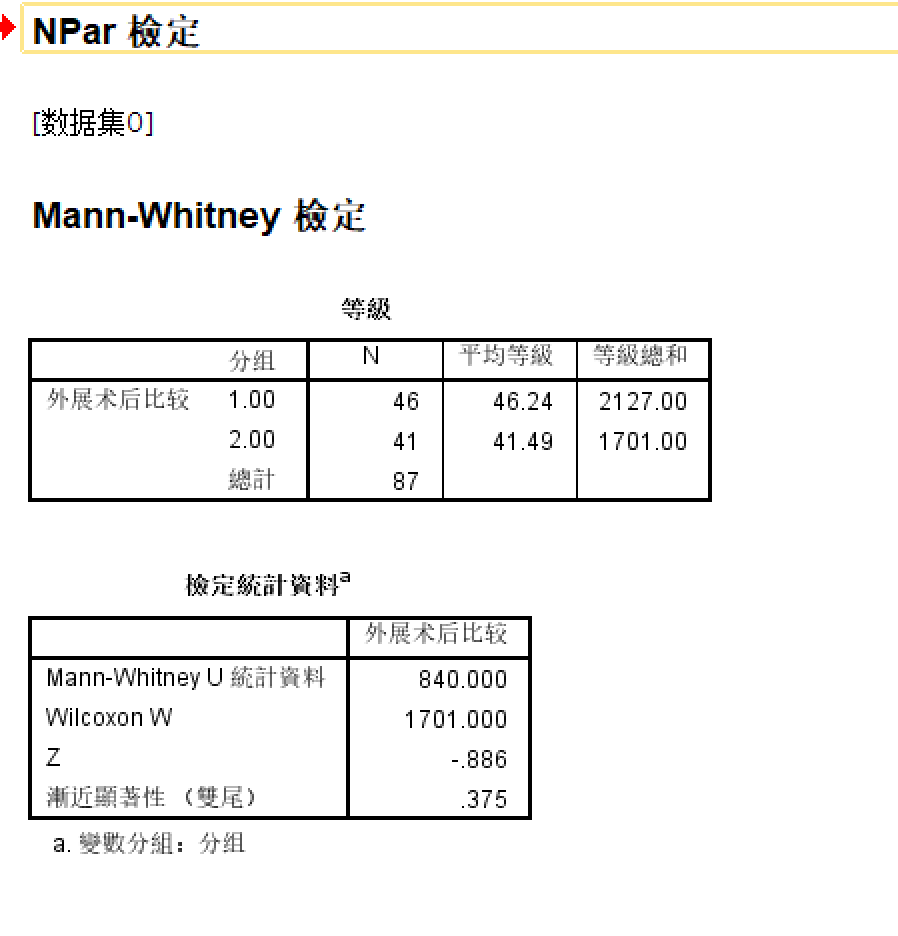

Supplement: Supplementary file 29 — Supplementary file29 (PNG 49 KB) [file 43465_2025_1645_MOESM29_ESM.png]

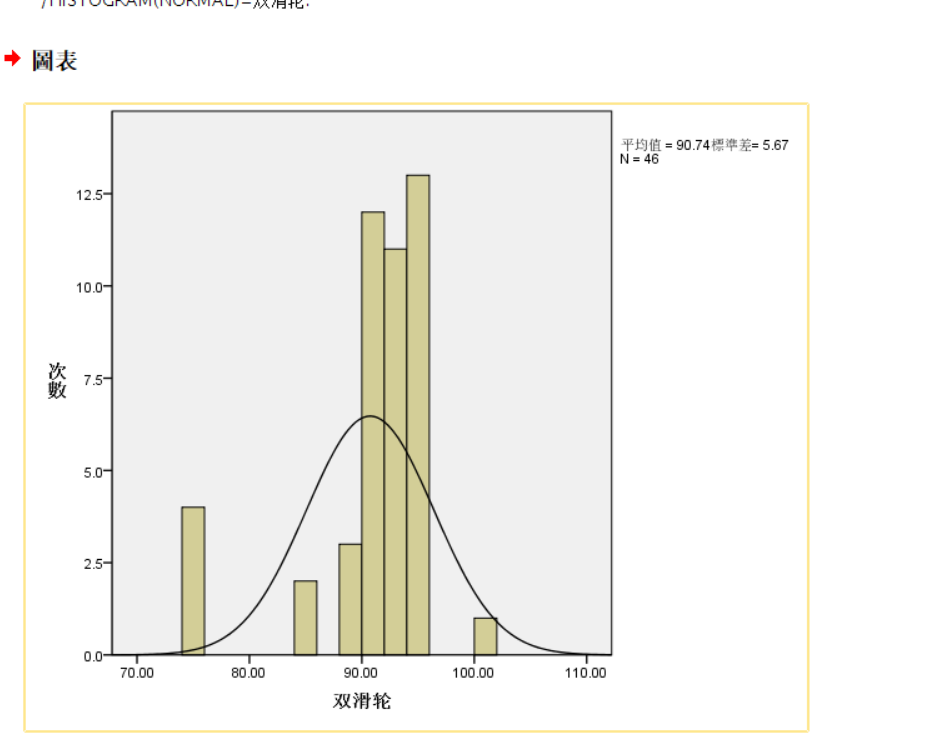

Supplement: Supplementary file 30 — Supplementary file30 (PNG 29 KB) [file 43465_2025_1645_MOESM30_ESM.png]

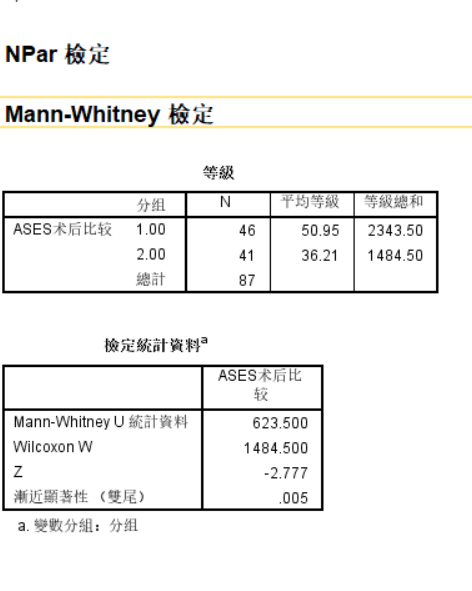

Supplement: Supplementary file 31 — Supplementary file31 (PNG 32 KB) [file 43465_2025_1645_MOESM31_ESM.png]

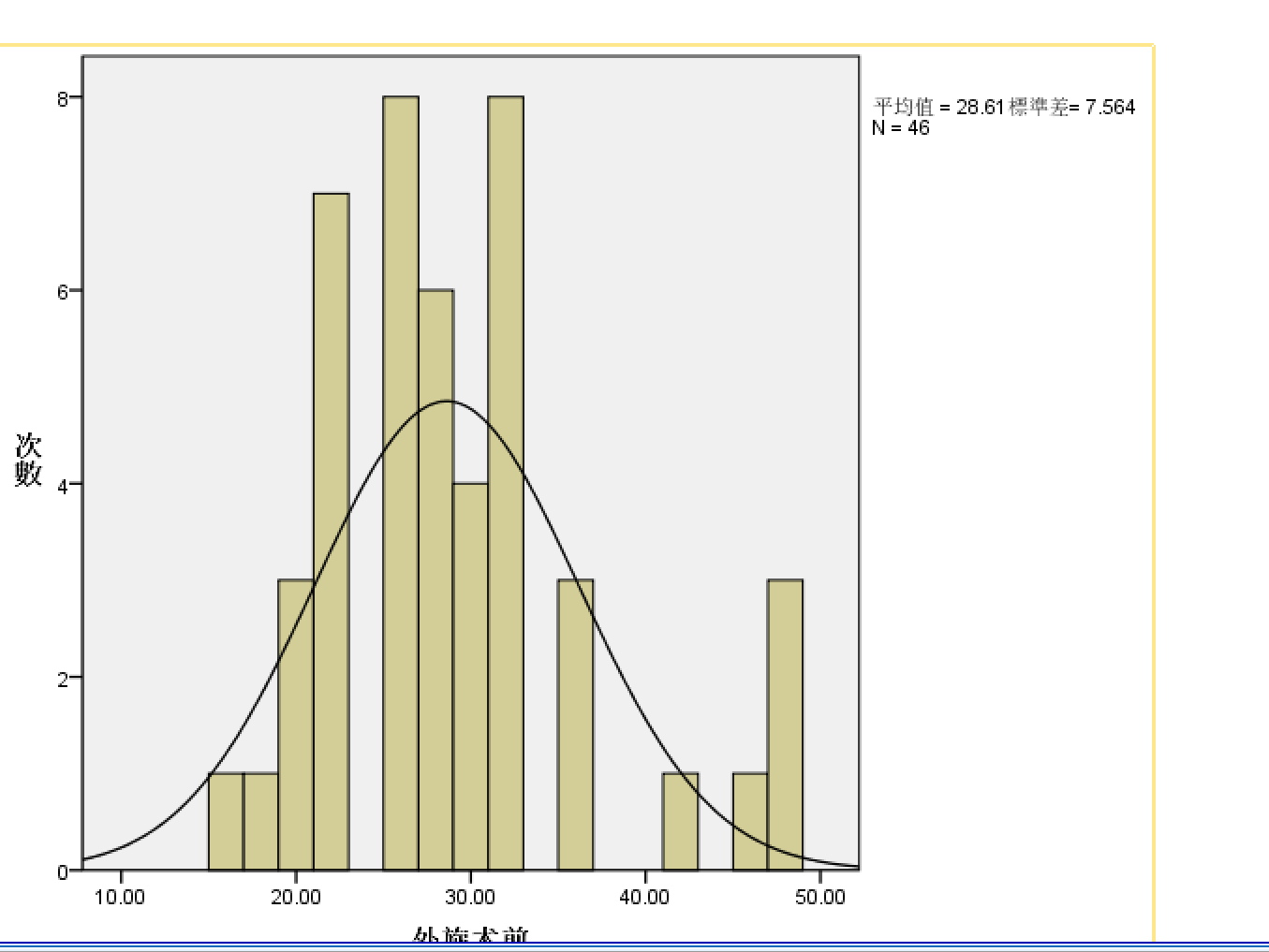

Supplement: Supplementary file 32 — Supplementary file32 (PNG 32 KB) [file 43465_2025_1645_MOESM32_ESM.png]

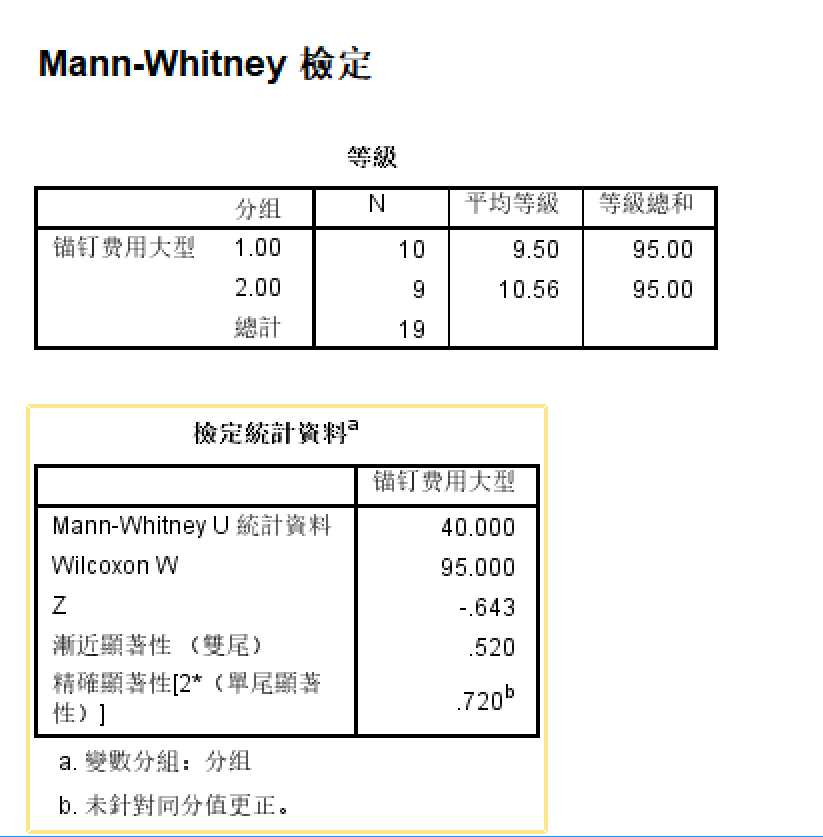

Supplement: Supplementary file 33 — Supplementary file33 (PNG 46 KB) [file 43465_2025_1645_MOESM33_ESM.png]

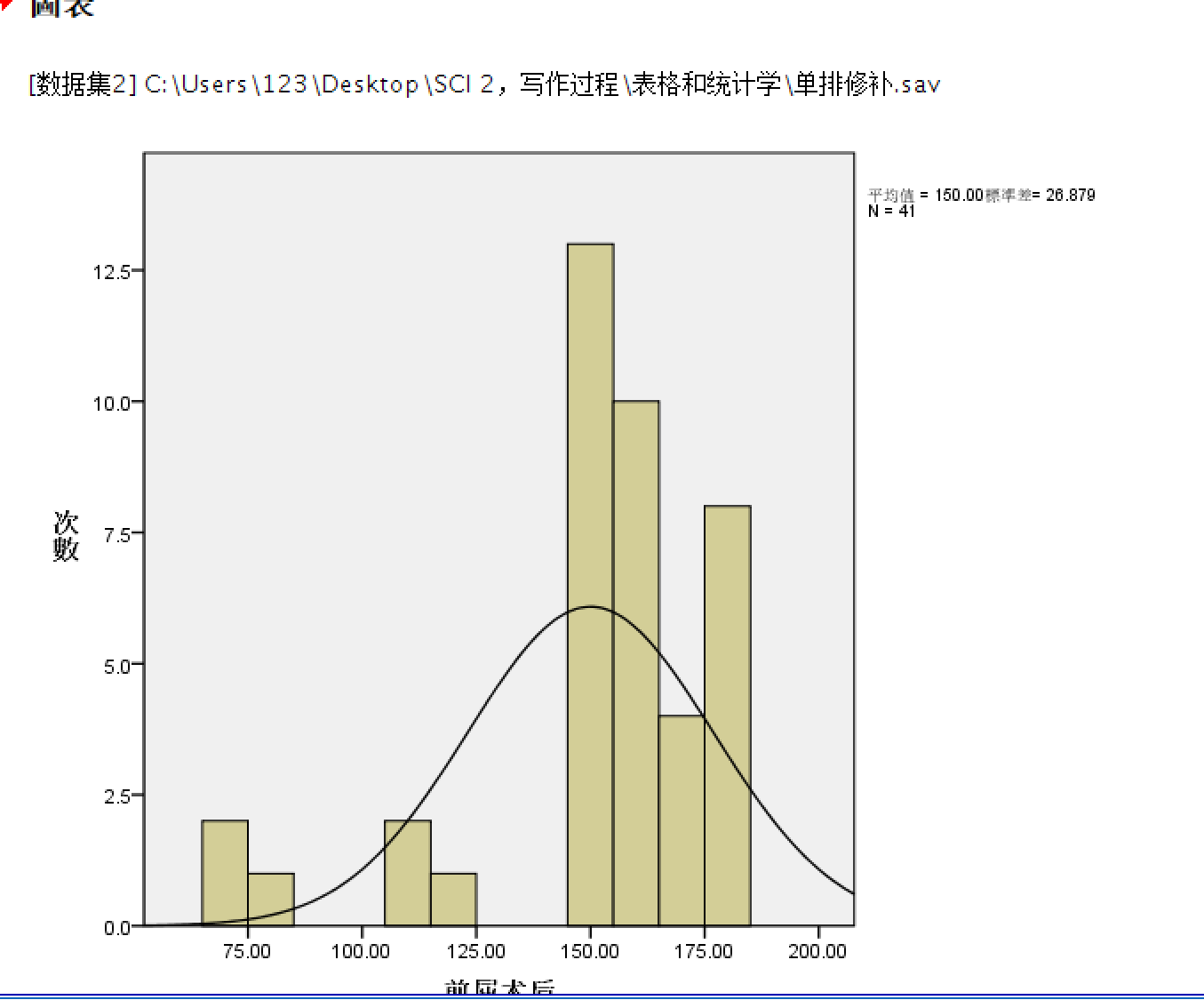

Supplement: Supplementary file 34 — Supplementary file34 (PNG 39 KB) [file 43465_2025_1645_MOESM34_ESM.png]

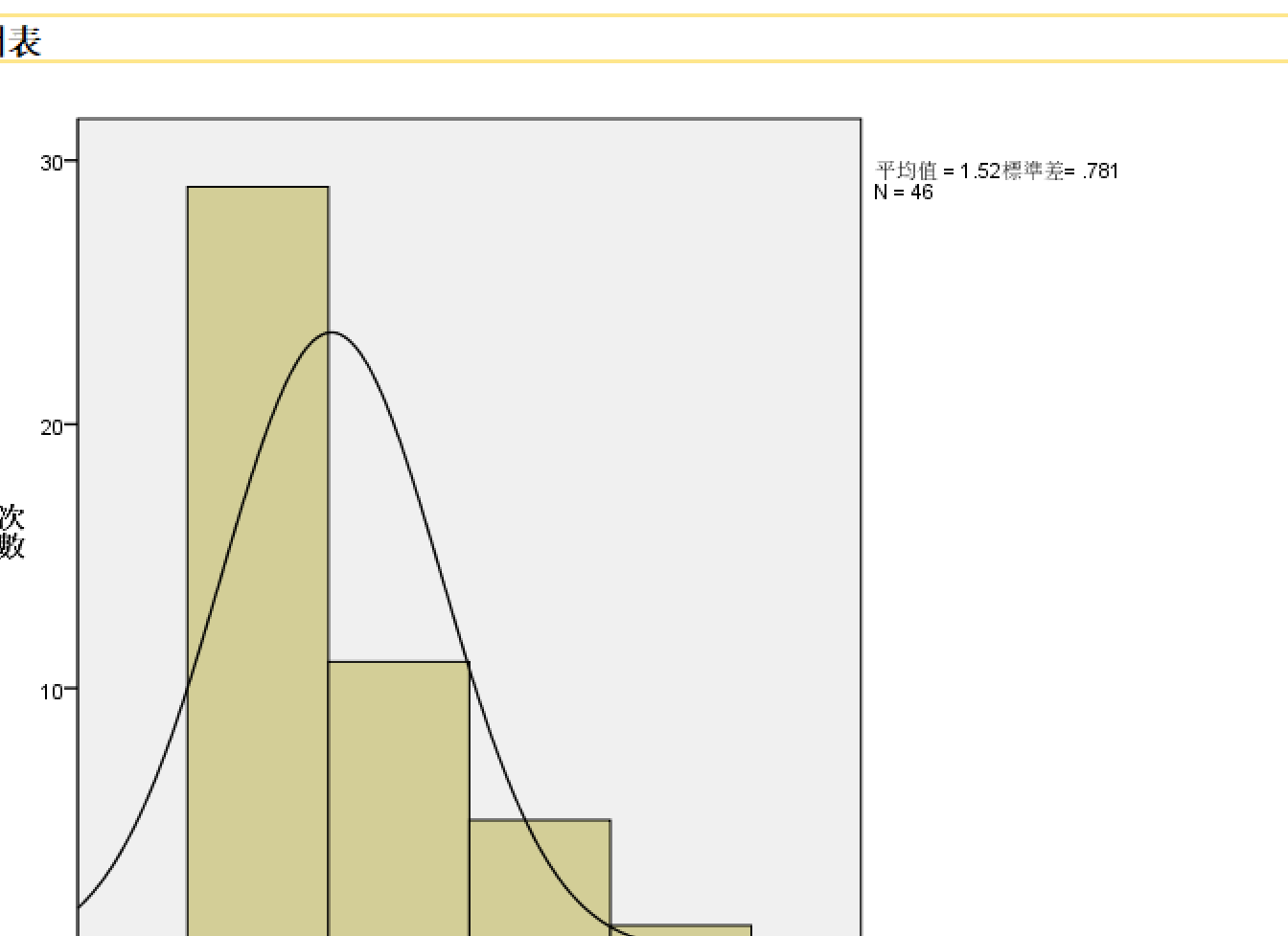

Supplement: Supplementary file 35 — Supplementary file35 (PNG 39 KB) [file 43465_2025_1645_MOESM35_ESM.png]

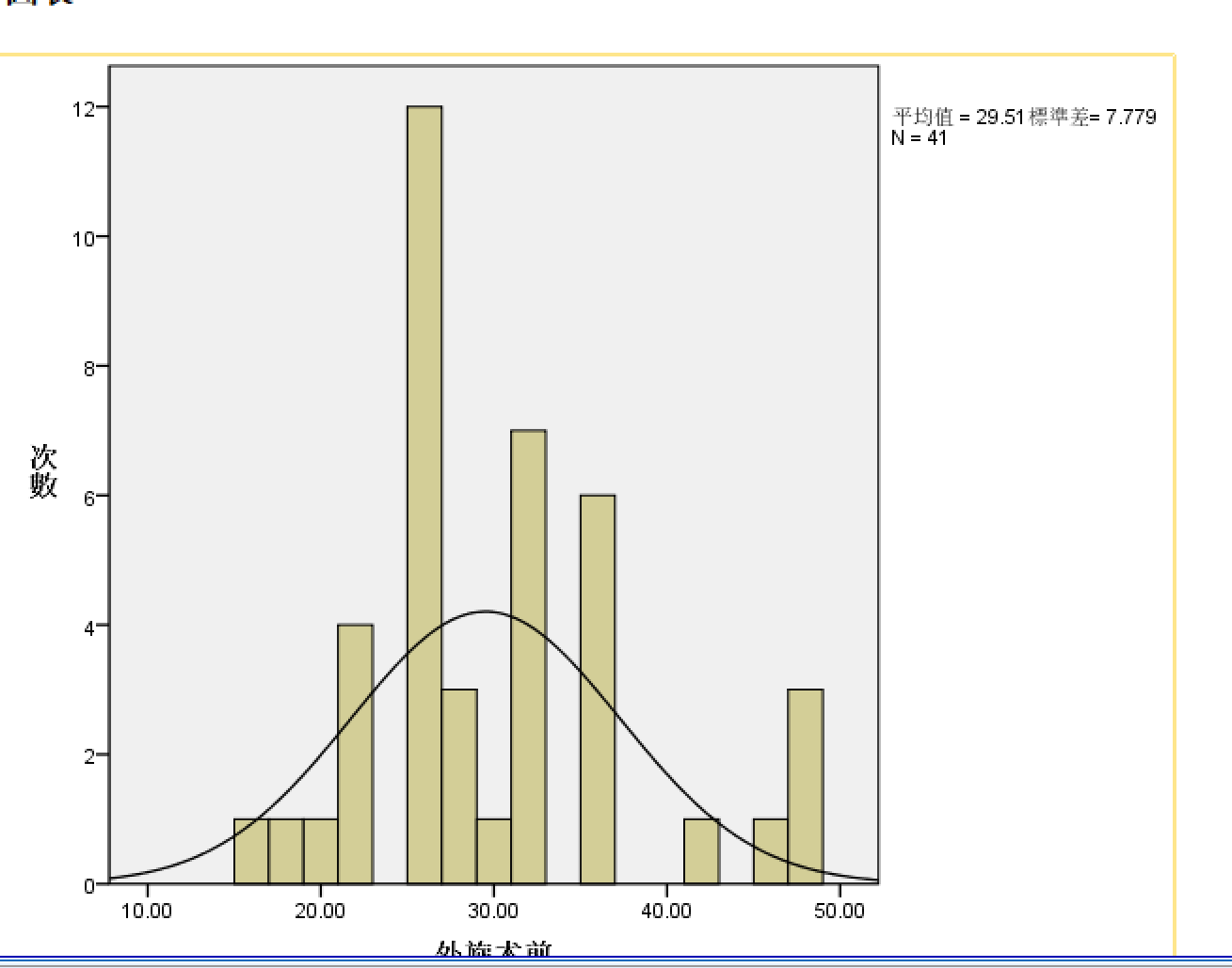

Supplement: Supplementary file 36 — Supplementary file36 (PNG 60 KB) [file 43465_2025_1645_MOESM36_ESM.png]

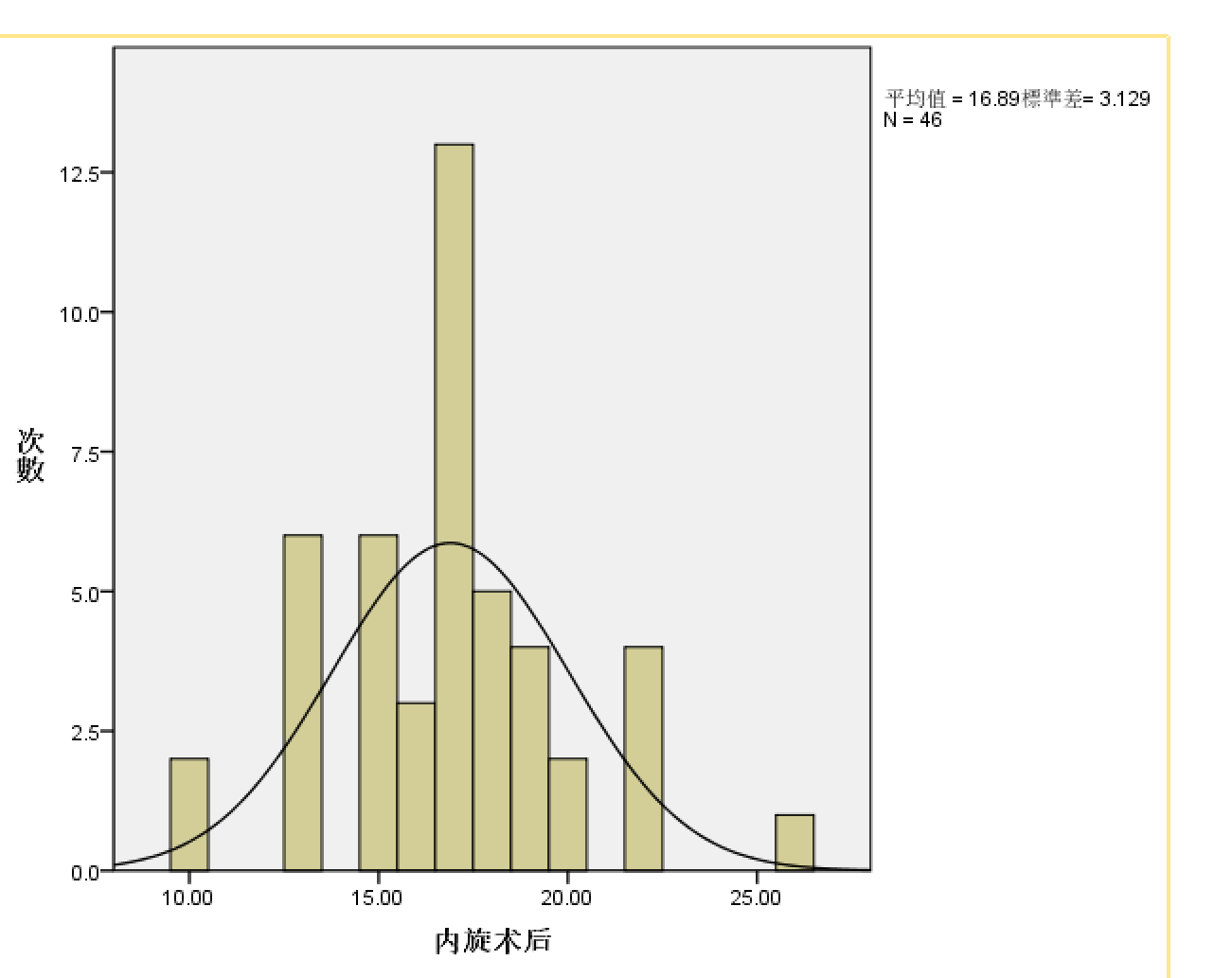

Supplement: Supplementary file 37 — Supplementary file37 (PNG 29 KB) [file 43465_2025_1645_MOESM37_ESM.png]

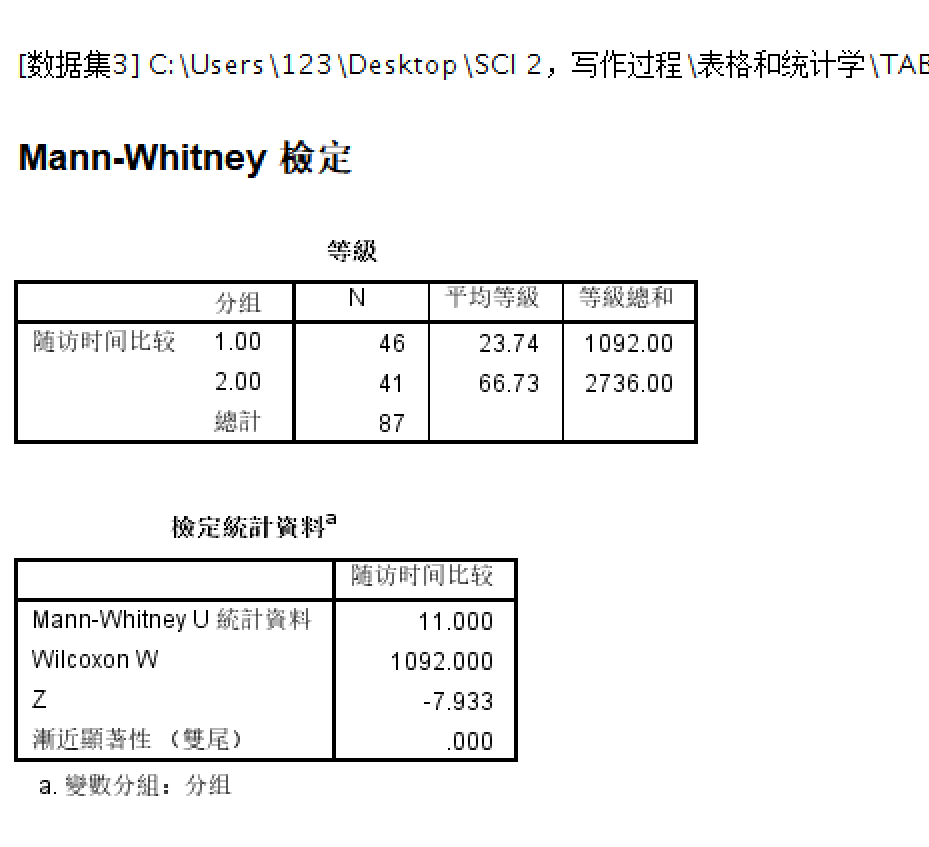

Supplement: Supplementary file 38 — Supplementary file38 (PNG 28 KB) [file 43465_2025_1645_MOESM38_ESM.png]

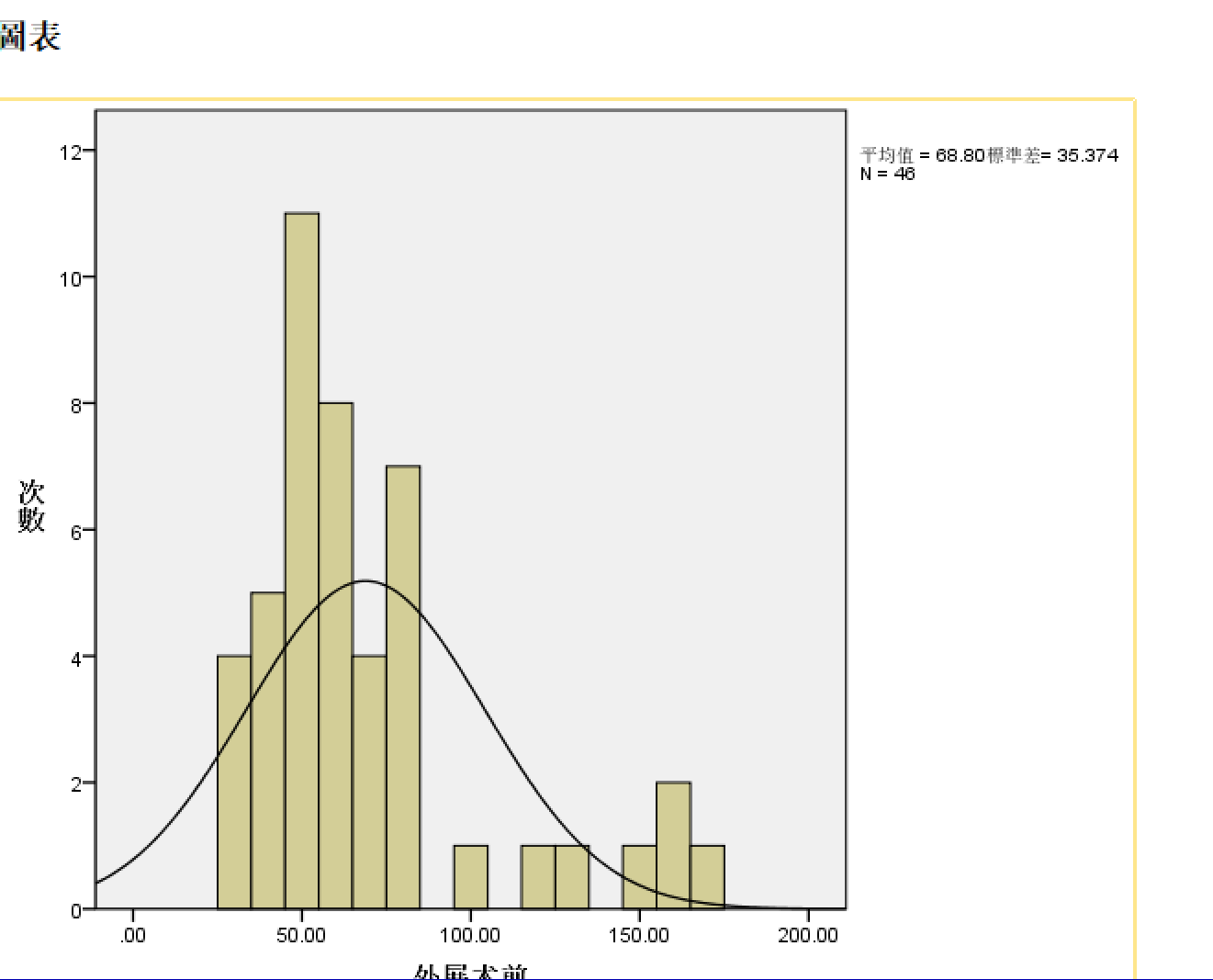

Supplement: Supplementary file 39 — Supplementary file39 (PNG 41 KB) [file 43465_2025_1645_MOESM39_ESM.png]

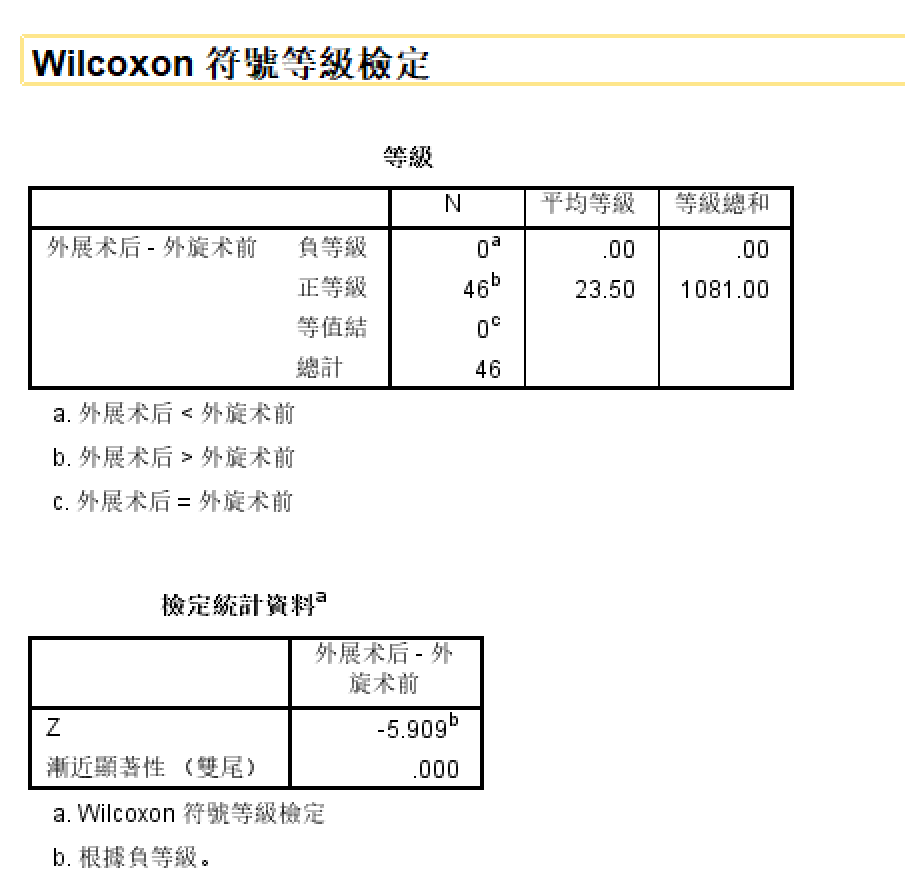

Supplement: Supplementary file 40 — Supplementary file40 (PNG 43 KB) [file 43465_2025_1645_MOESM40_ESM.png]

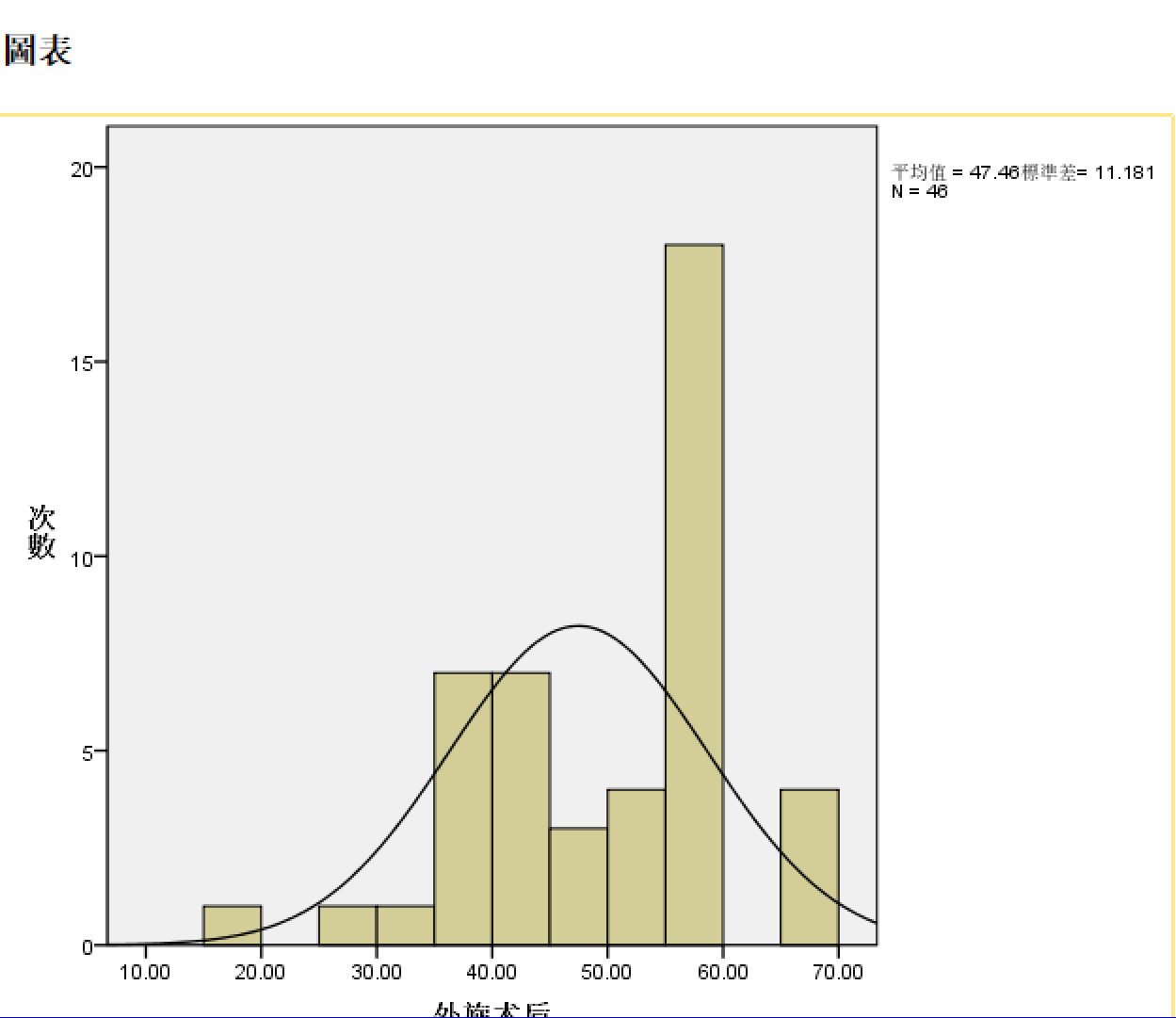

Supplement: Supplementary file 41 — Supplementary file41 (PNG 44 KB) [file 43465_2025_1645_MOESM41_ESM.png]

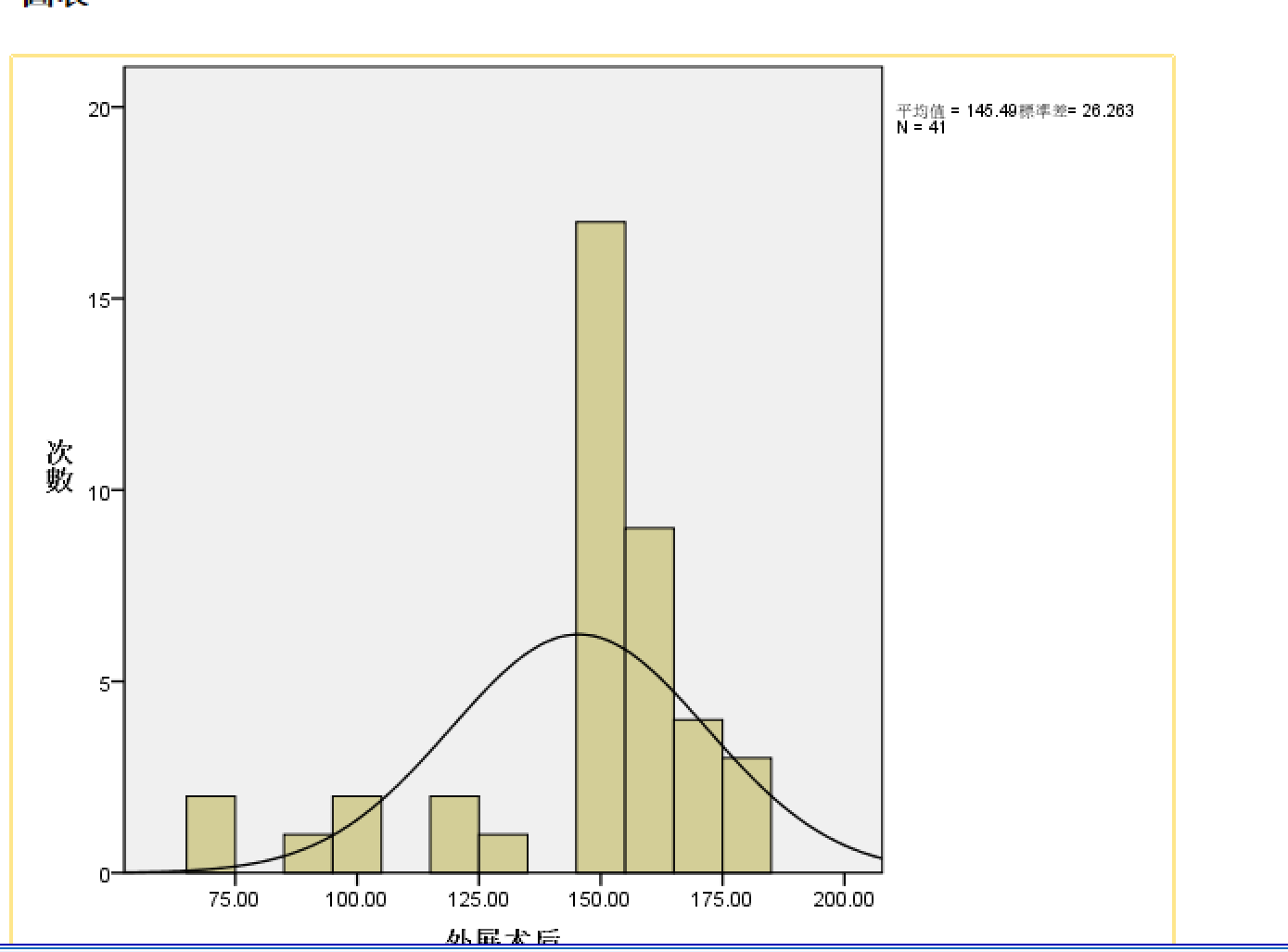

Supplement: Supplementary file 42 — Supplementary file42 (PNG 44 KB) [file 43465_2025_1645_MOESM42_ESM.png]

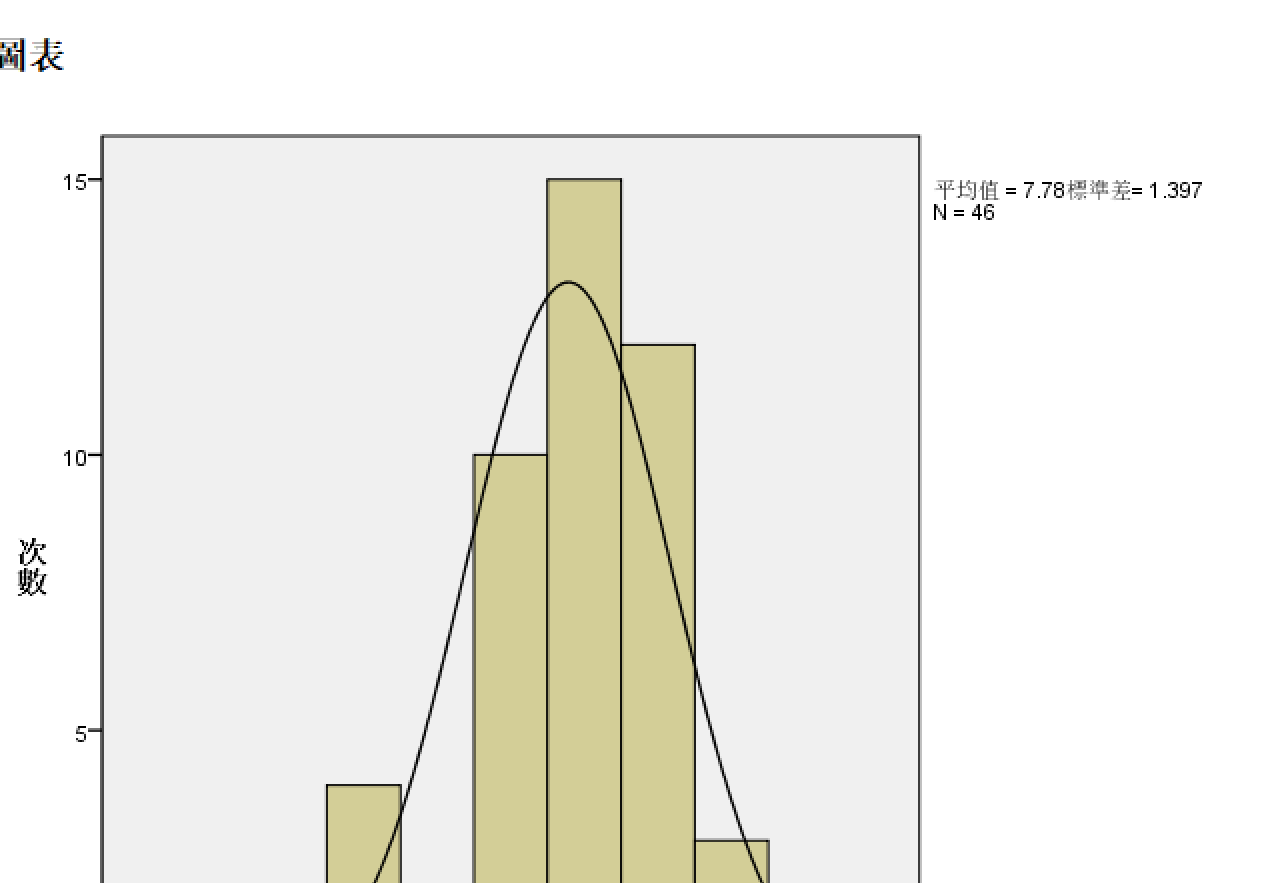

Supplement: Supplementary file 43 — Supplementary file43 (PNG 47 KB) [file 43465_2025_1645_MOESM43_ESM.png]

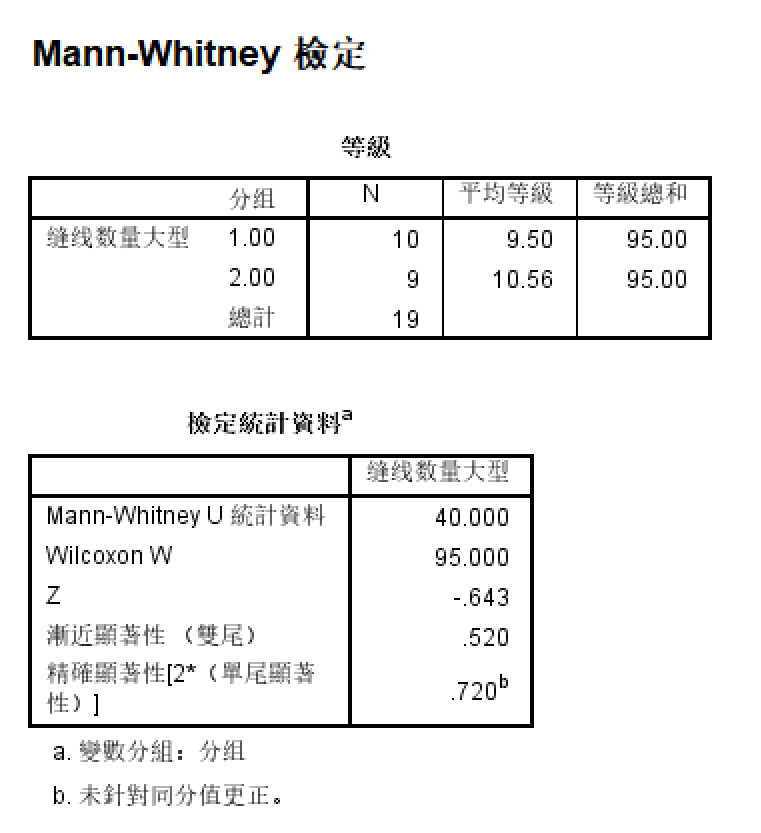

Supplement: Supplementary file 44 — Supplementary file44 (PNG 48 KB) [file 43465_2025_1645_MOESM44_ESM.png]

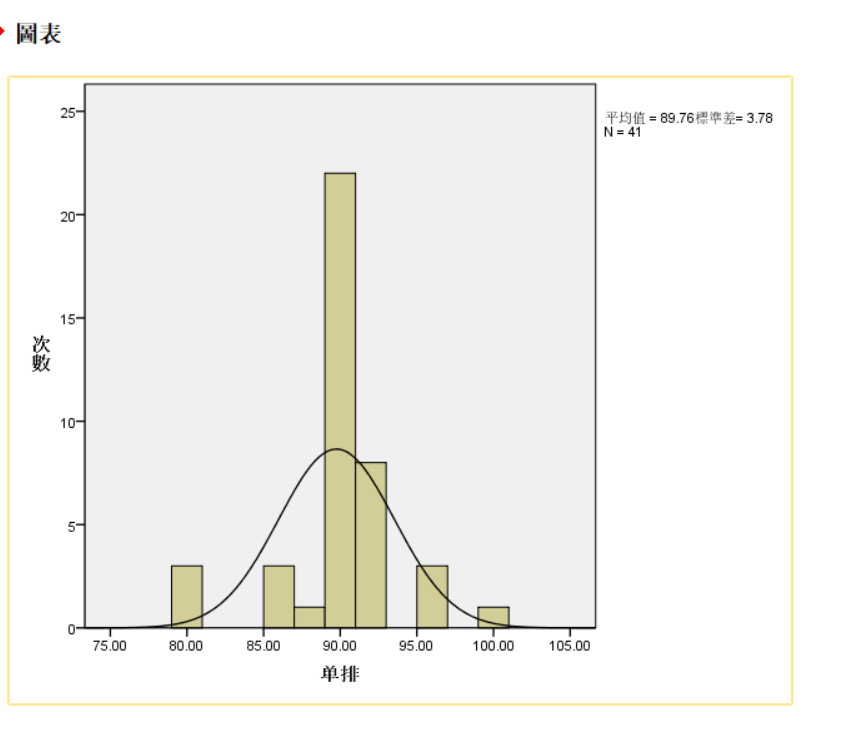

Supplement: Supplementary file 45 — Supplementary file45 (PNG 42 KB) [file 43465_2025_1645_MOESM45_ESM.png]

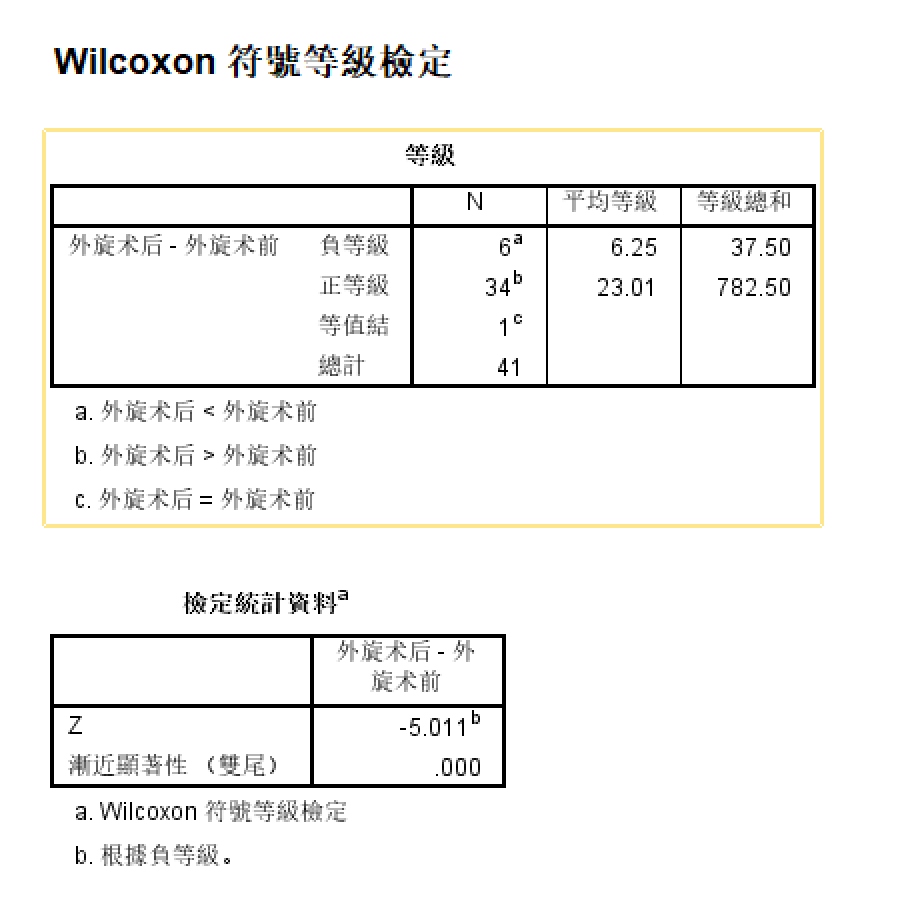

Supplement: Supplementary file 46 — Supplementary file46 (PNG 41 KB) [file 43465_2025_1645_MOESM46_ESM.png]

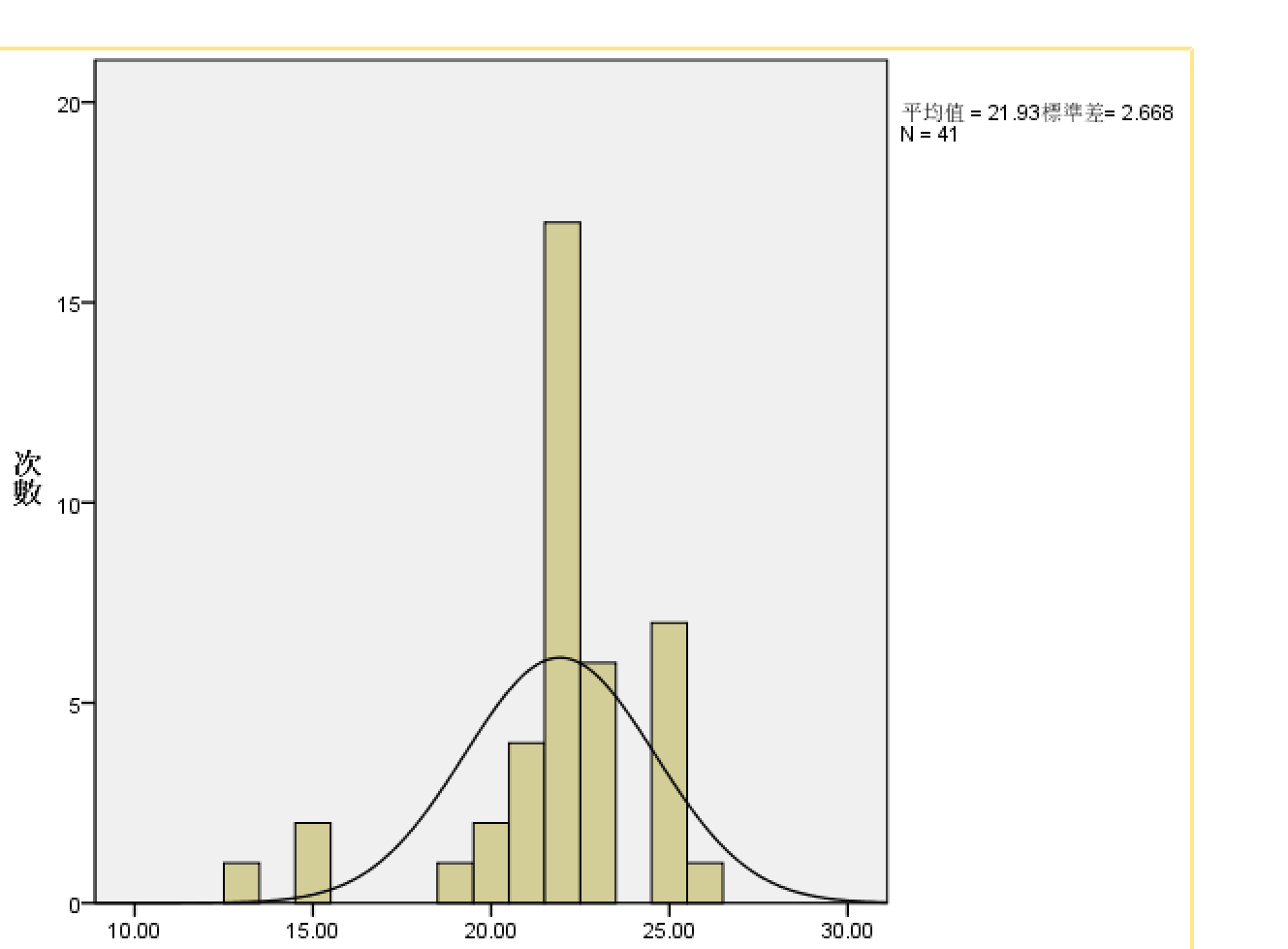

Supplement: Supplementary file 47 — Supplementary file47 (PNG 39 KB) [file 43465_2025_1645_MOESM47_ESM.png]

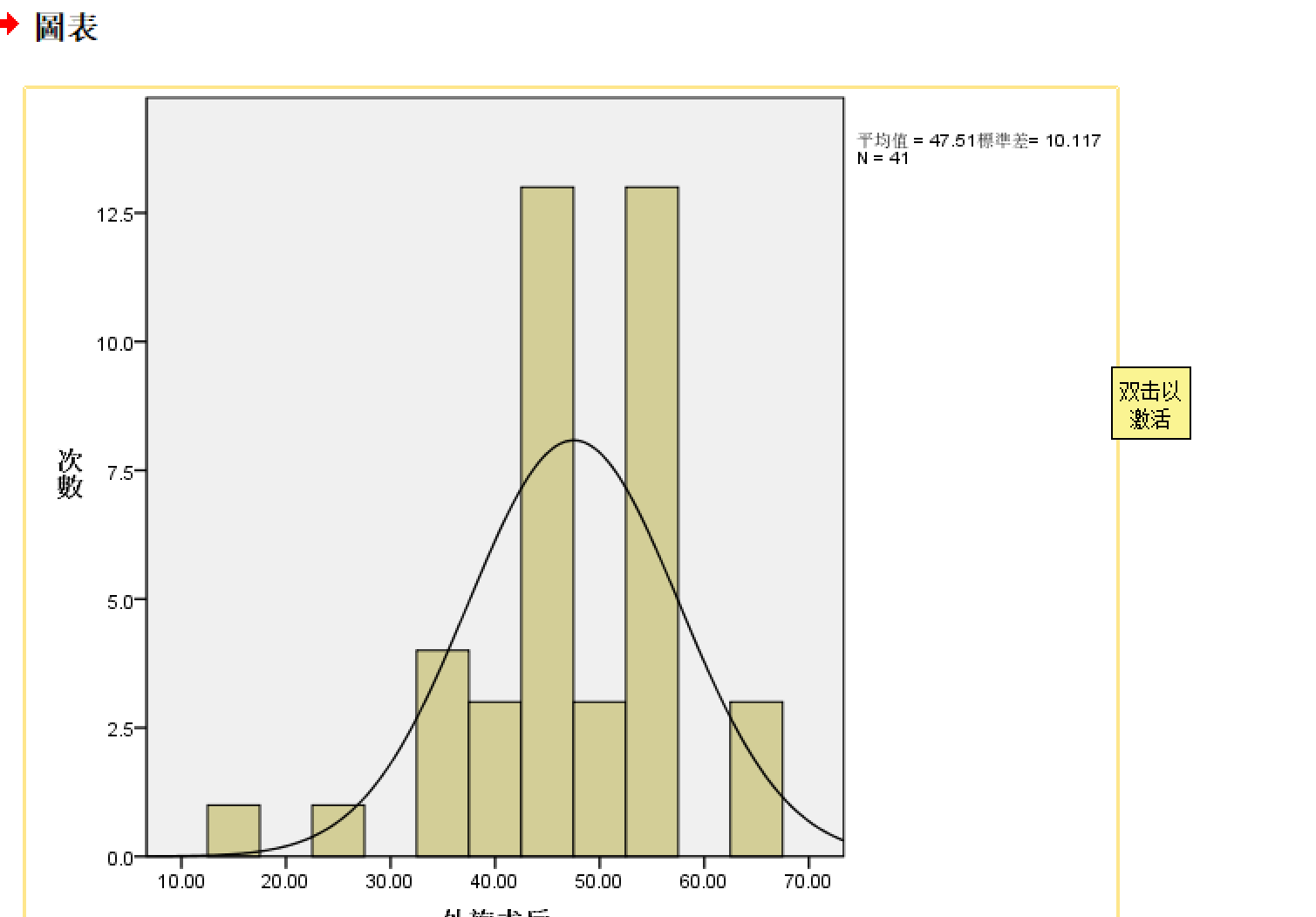

Supplement: Supplementary file 48 — Supplementary file48 (PNG 35 KB) [file 43465_2025_1645_MOESM48_ESM.png]

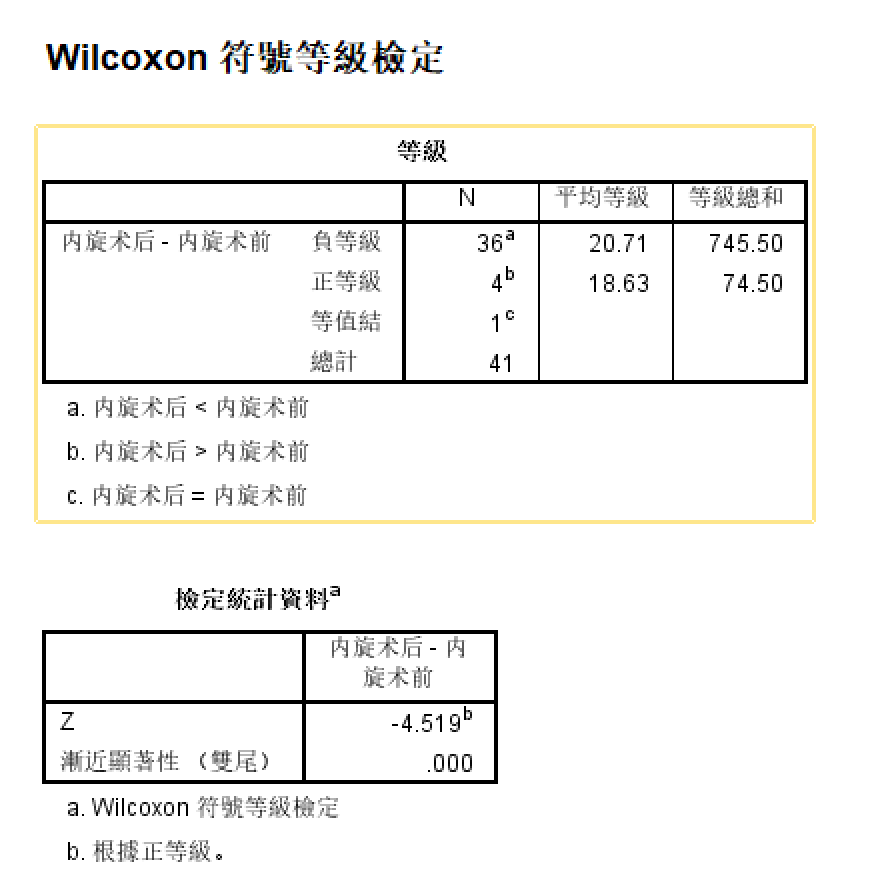

Supplement: Supplementary file 49 — Supplementary file49 (PNG 37 KB) [file 43465_2025_1645_MOESM49_ESM.png]

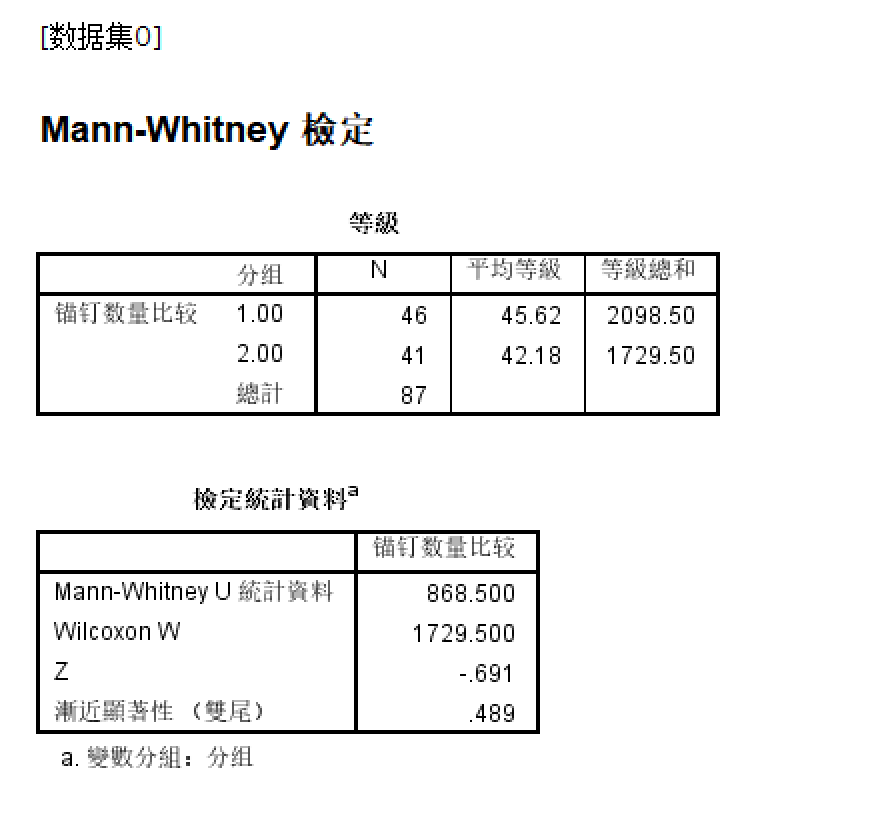

Supplement: Supplementary file 50 — Supplementary file50 (PNG 38 KB) [file 43465_2025_1645_MOESM50_ESM.png]

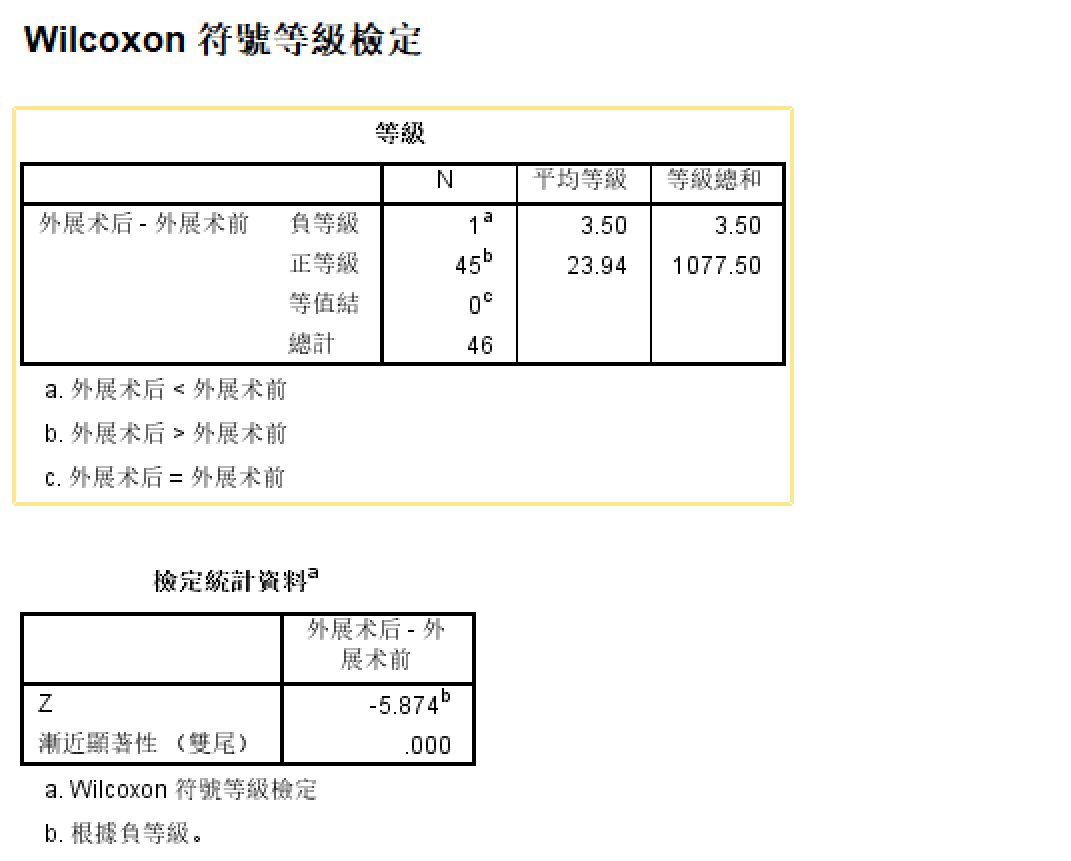

Supplement: Supplementary file 51 — Supplementary file51 (PNG 35 KB) [file 43465_2025_1645_MOESM51_ESM.png]

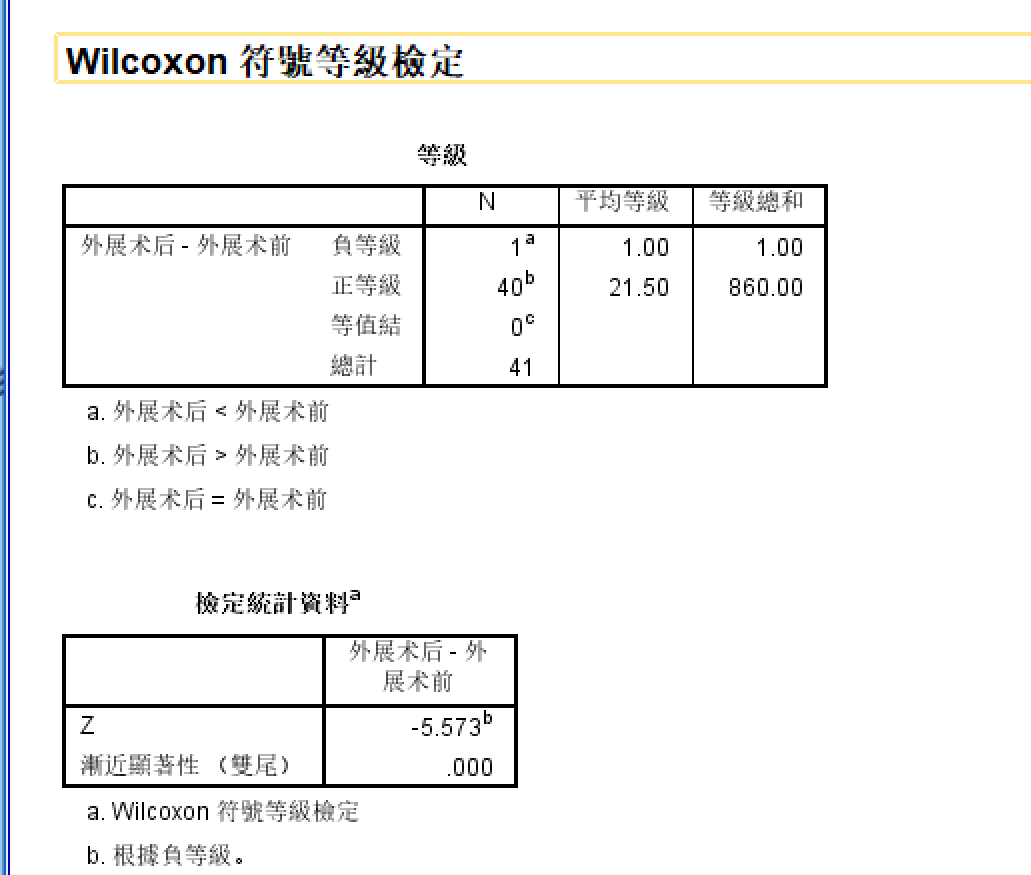

Supplement: Supplementary file 52 — Supplementary file52 (PNG 34 KB) [file 43465_2025_1645_MOESM52_ESM.png]

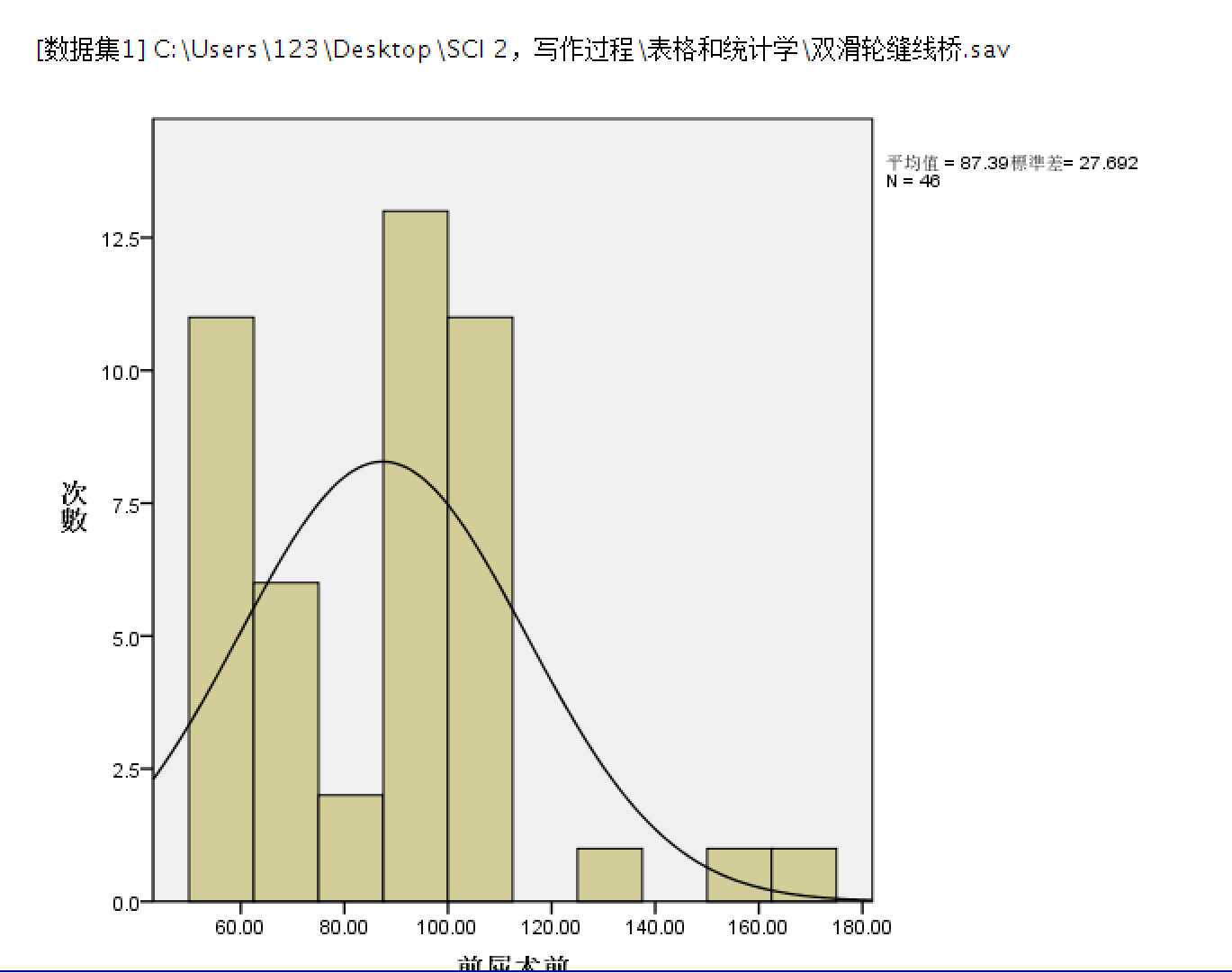

Supplement: Supplementary file 53 — Supplementary file53 (PNG 34 KB) [file 43465_2025_1645_MOESM53_ESM.png]

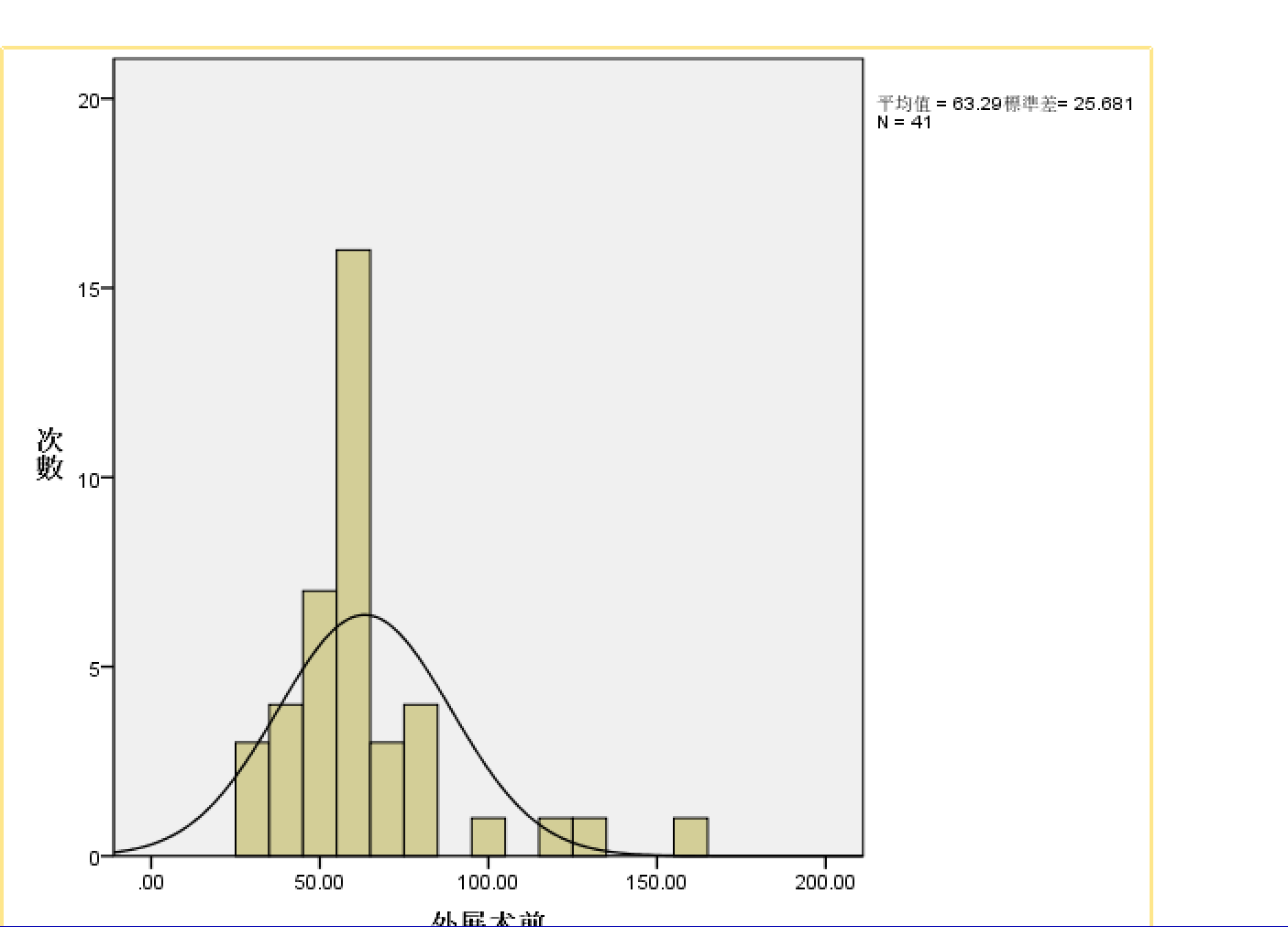

Supplement: Supplementary file 54 — Supplementary file54 (PNG 36 KB) [file 43465_2025_1645_MOESM54_ESM.png]

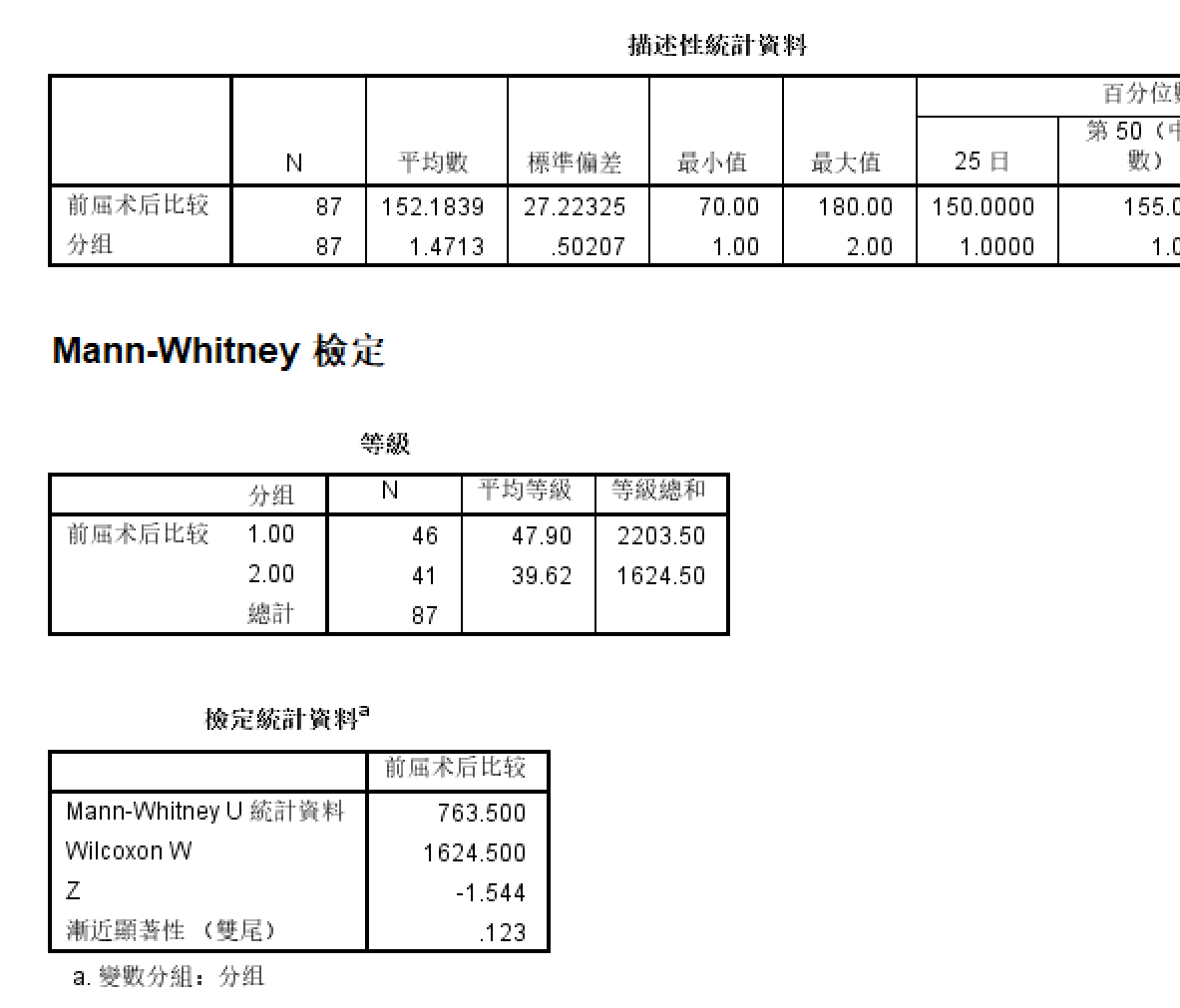

Supplement: Supplementary file 55 — Supplementary file55 (PNG 40 KB) [file 43465_2025_1645_MOESM55_ESM.png]

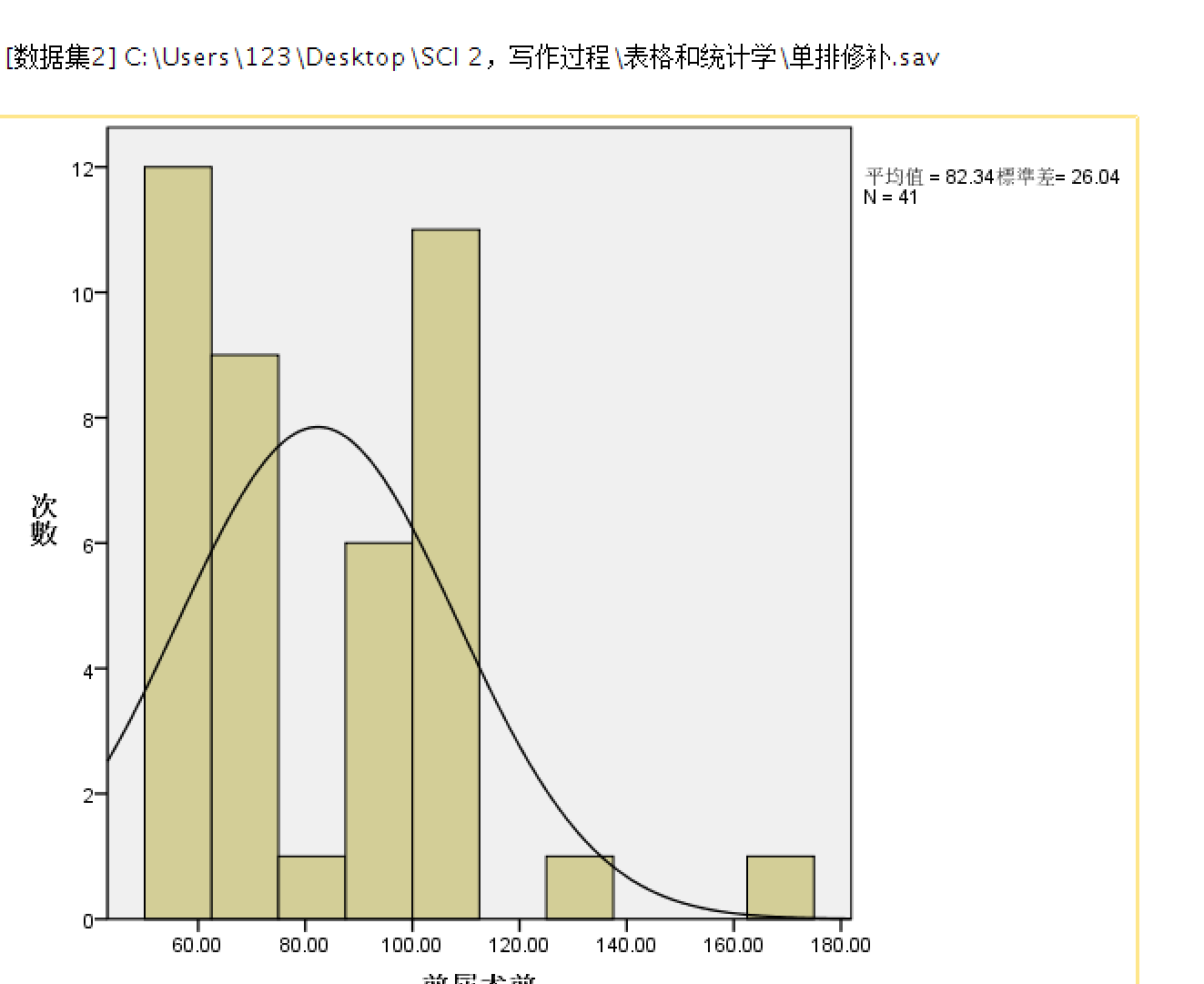

Supplement: Supplementary file 56 — Supplementary file56 (PNG 36 KB) [file 43465_2025_1645_MOESM56_ESM.png]

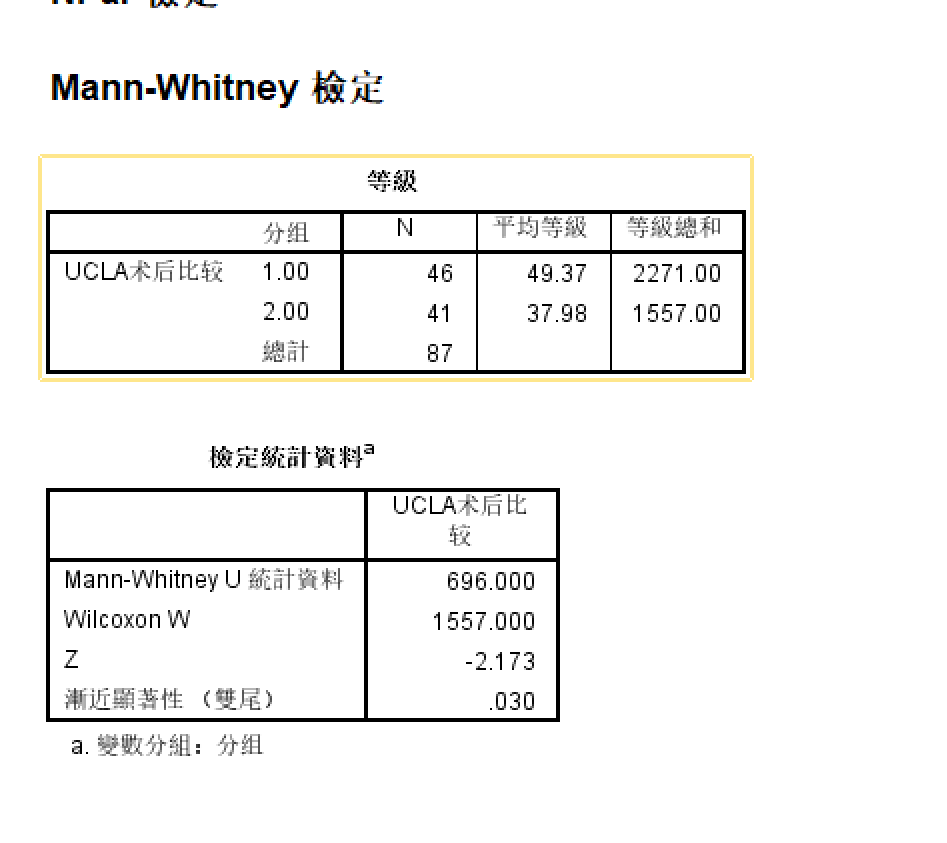

Supplement: Supplementary file 57 — Supplementary file57 (PNG 31 KB) [file 43465_2025_1645_MOESM57_ESM.png]

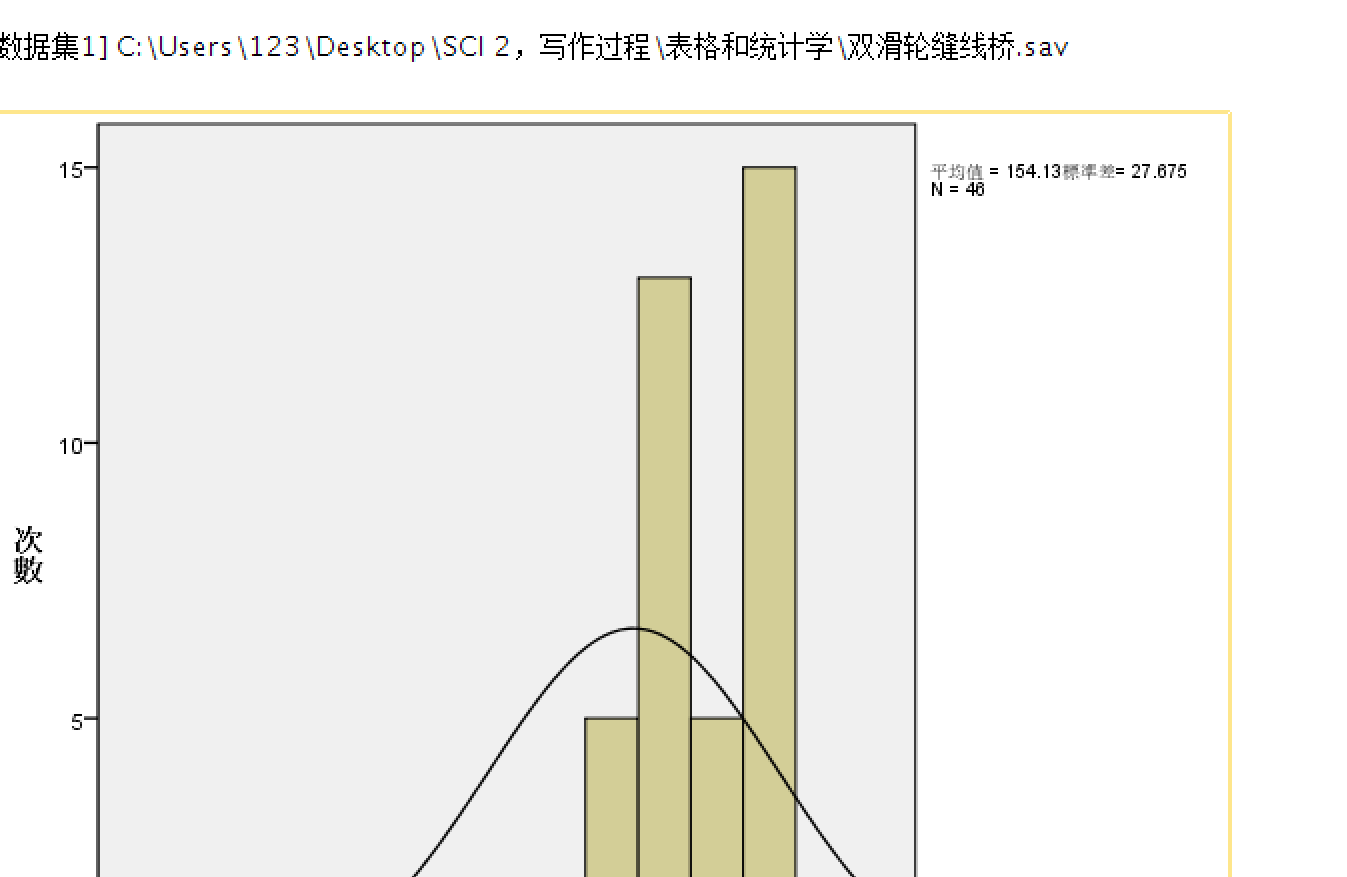

Supplement: Supplementary file 58 — Supplementary file58 (PNG 45 KB) [file 43465_2025_1645_MOESM58_ESM.png]

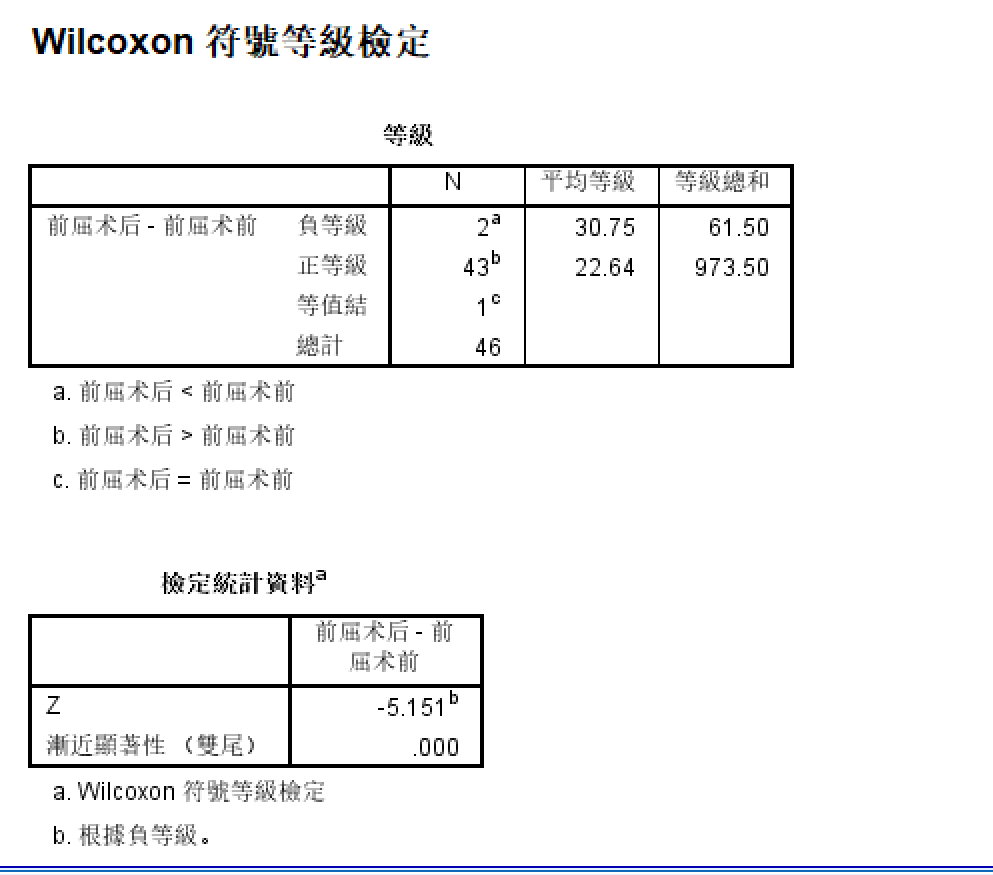

Supplement: Supplementary file 59 — Supplementary file59 (PNG 36 KB) [file 43465_2025_1645_MOESM59_ESM.png]

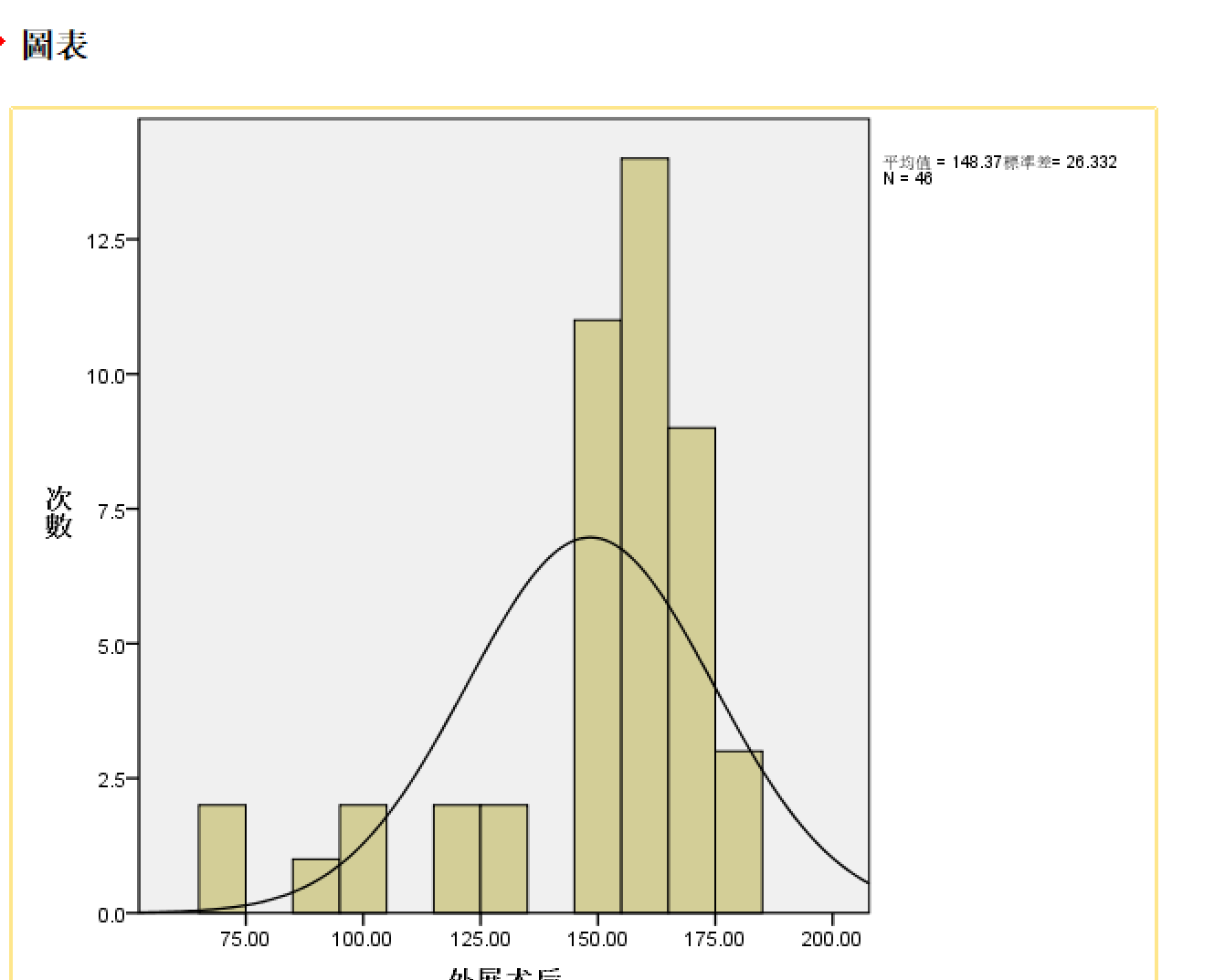

Supplement: Supplementary file 60 — Supplementary file60 (PNG 37 KB) [file 43465_2025_1645_MOESM60_ESM.png]

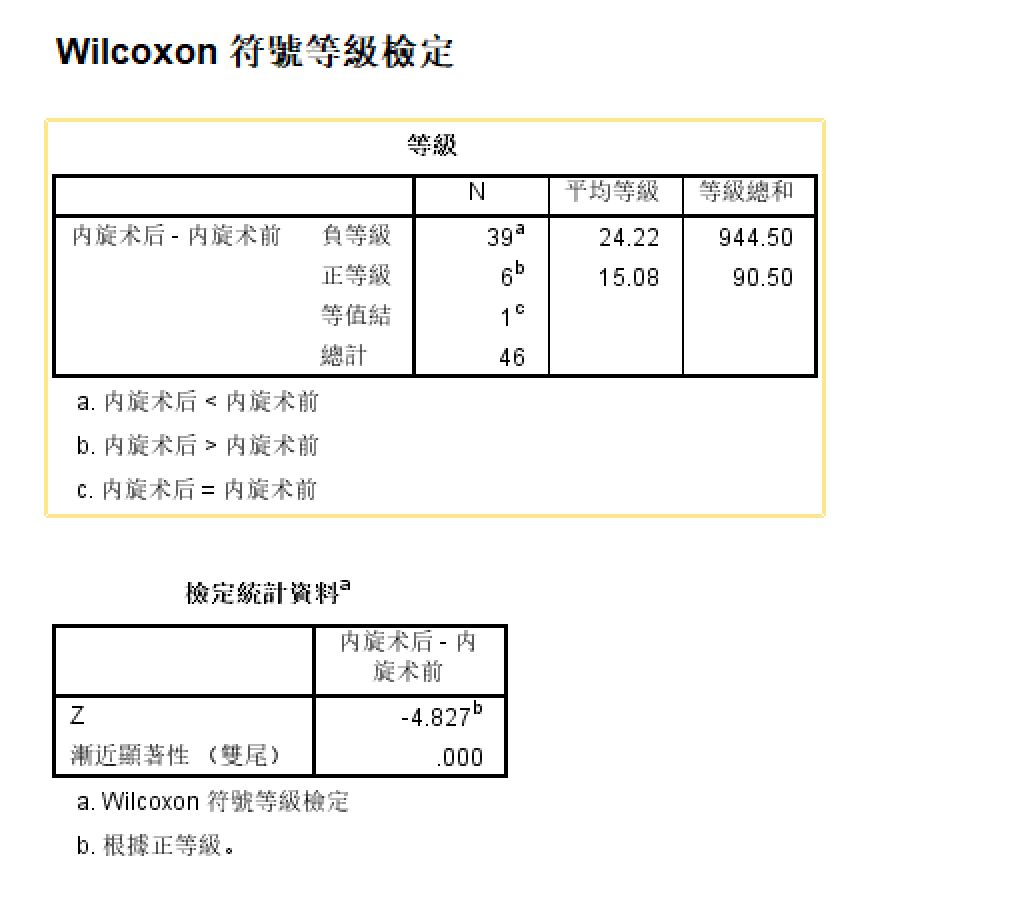

Supplement: Supplementary file 61 — Supplementary file61 (PNG 35 KB) [file 43465_2025_1645_MOESM61_ESM.png]

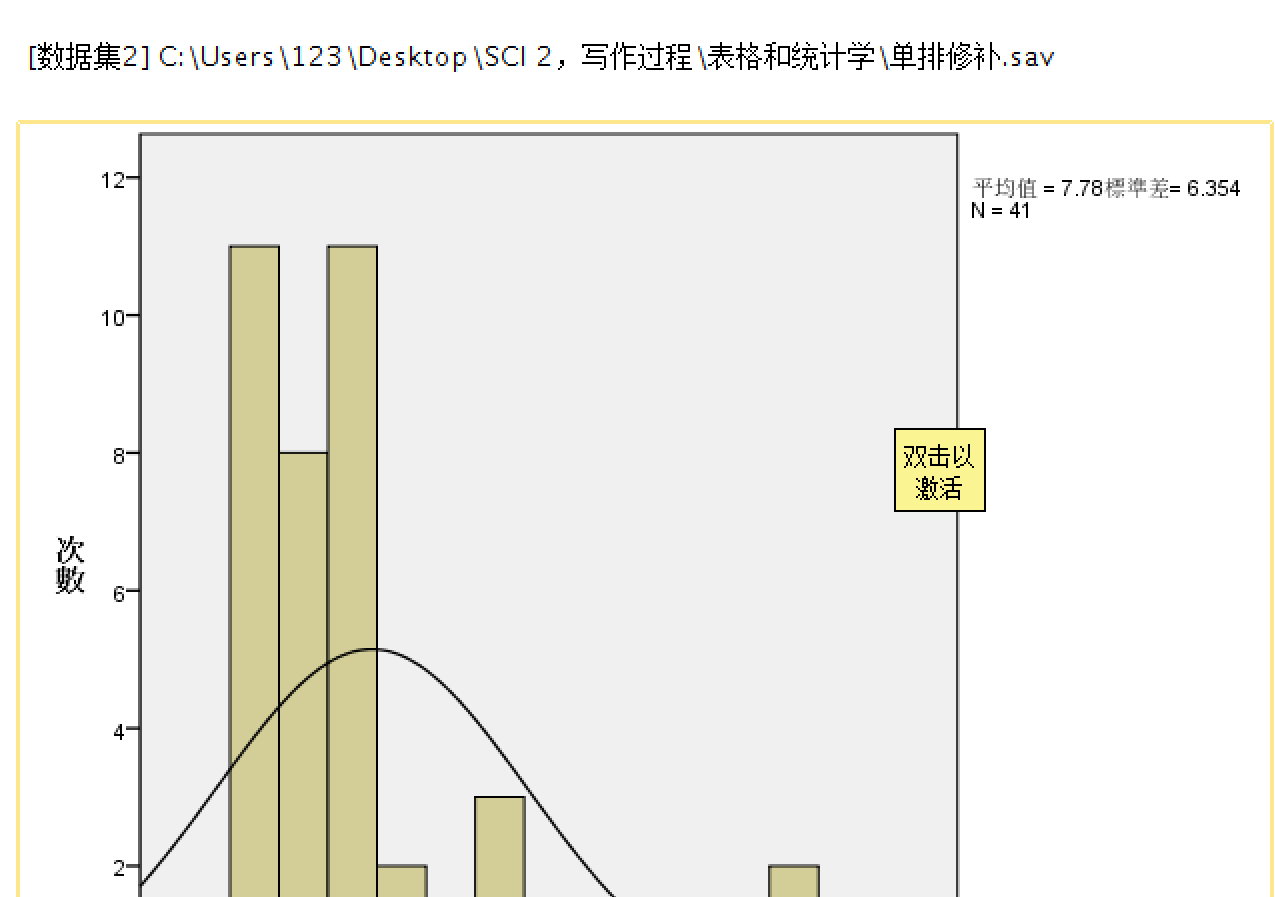

Supplement: Supplementary file 62 — Supplementary file62 (PNG 48 KB) [file 43465_2025_1645_MOESM62_ESM.png]

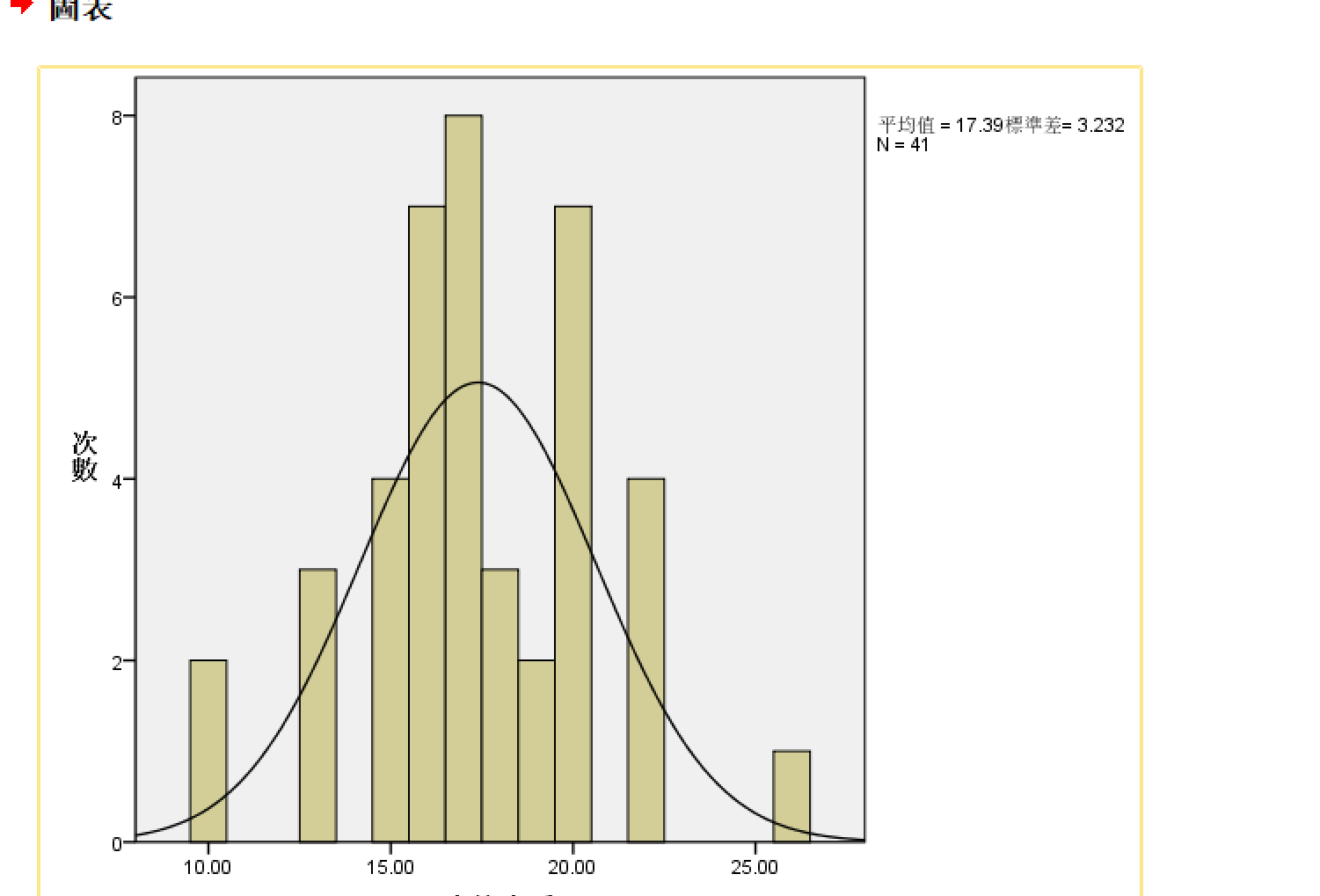

Supplement: Supplementary file 63 — Supplementary file63 (PNG 32 KB) [file 43465_2025_1645_MOESM63_ESM.png]

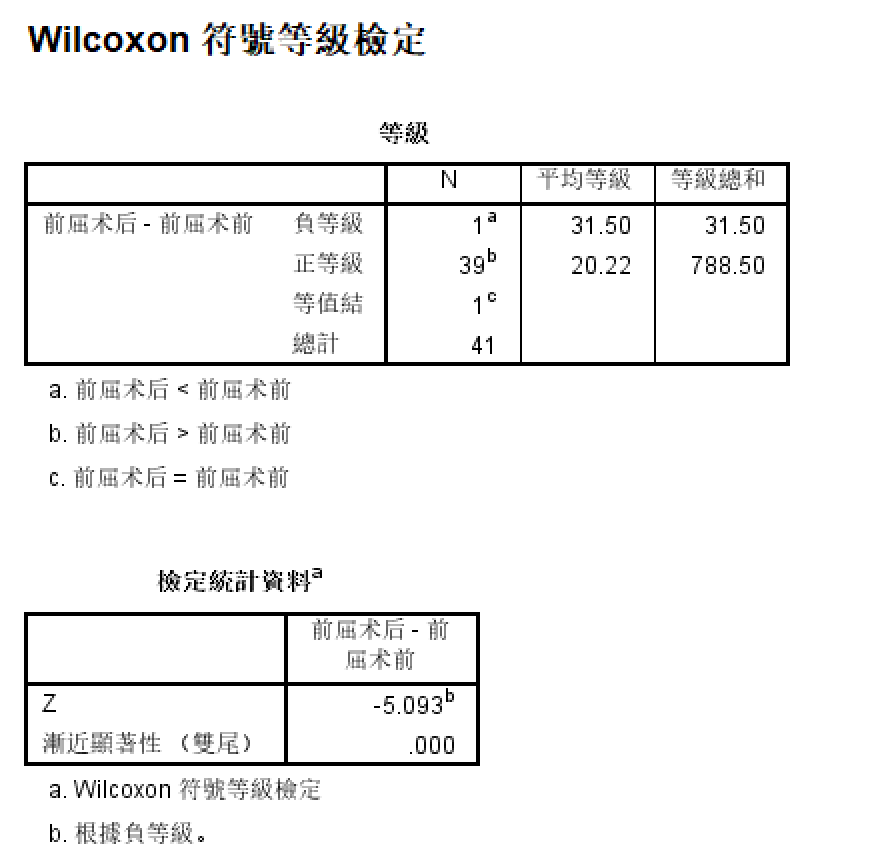

Supplement: Supplementary file 64 — Supplementary file64 (PNG 47 KB) [file 43465_2025_1645_MOESM64_ESM.png]

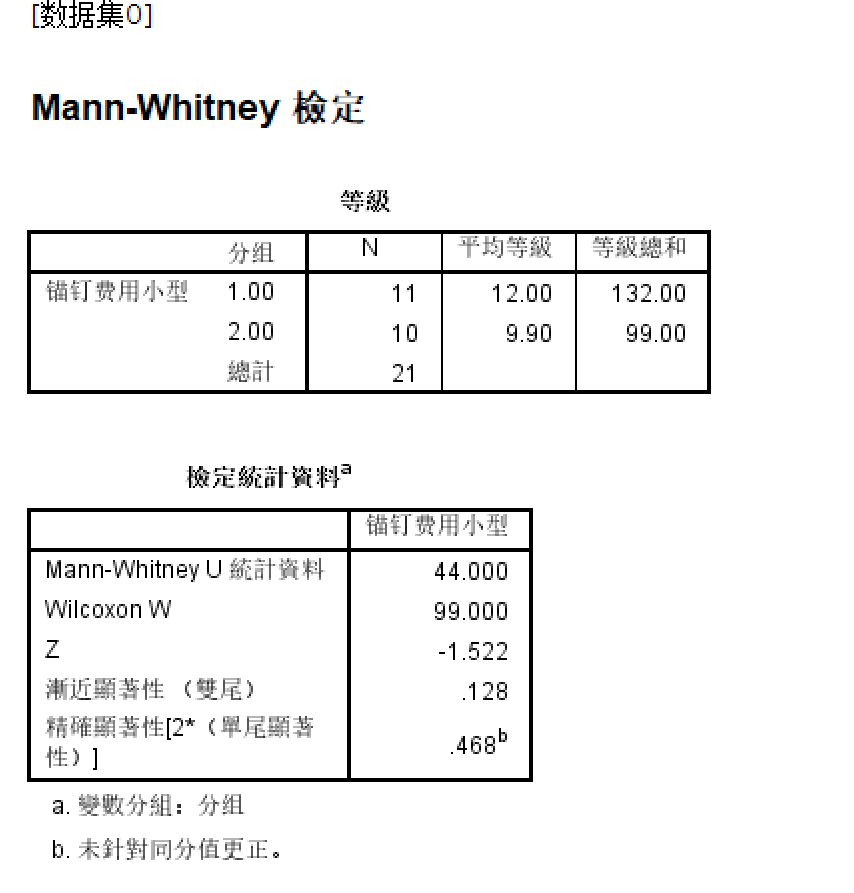

Supplement: Supplementary file 65 — Supplementary file65 (PNG 31 KB) [file 43465_2025_1645_MOESM65_ESM.png]

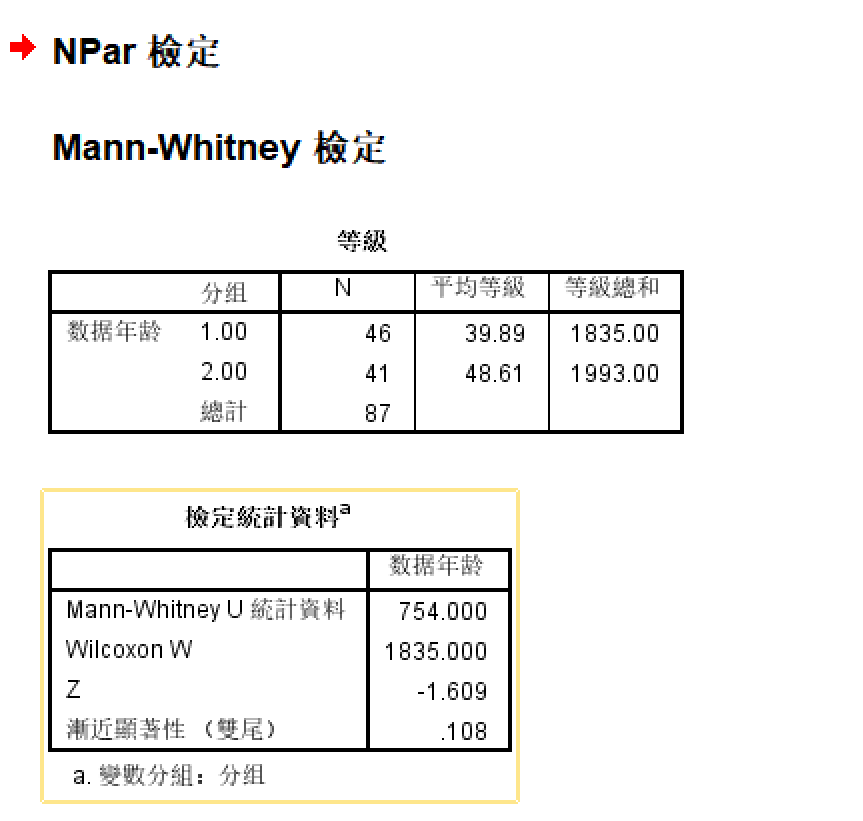

Supplement: Supplementary file 66 — Supplementary file66 (PNG 32 KB) [file 43465_2025_1645_MOESM66_ESM.png]

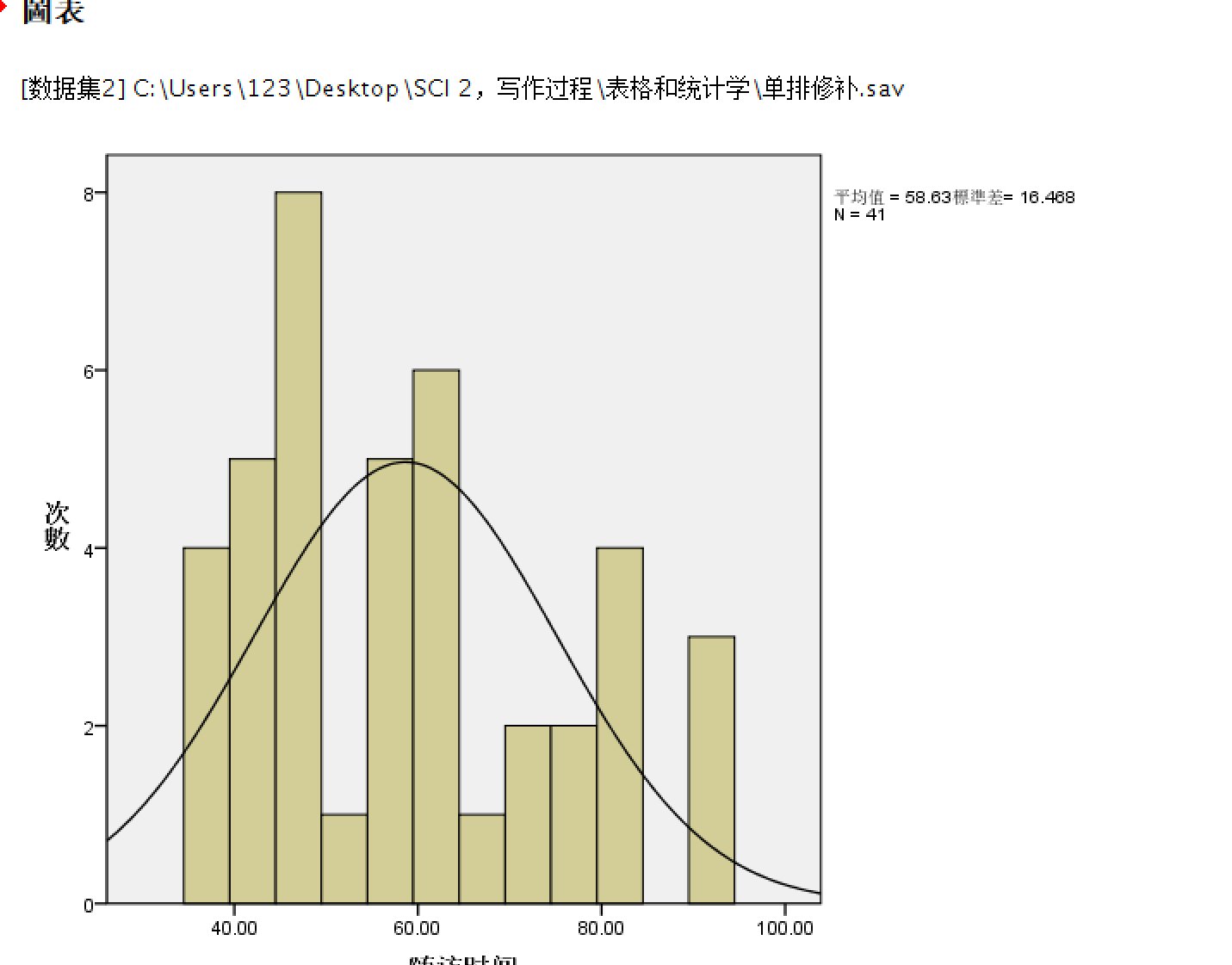

Supplement: Supplementary file 67 — Supplementary file67 (PNG 34 KB) [file 43465_2025_1645_MOESM67_ESM.png]

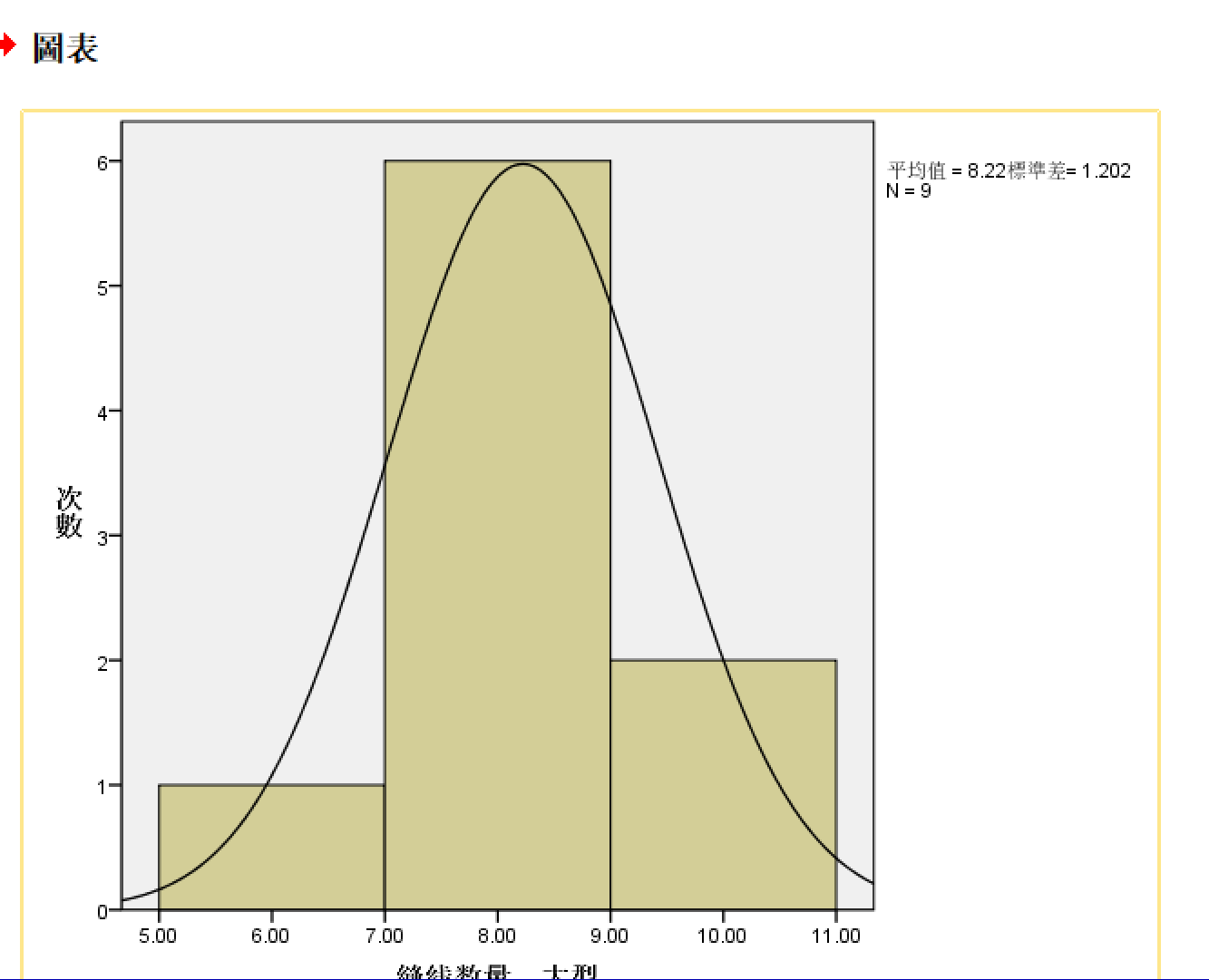

Supplement: Supplementary file 68 — Supplementary file68 (PNG 39 KB) [file 43465_2025_1645_MOESM68_ESM.png]

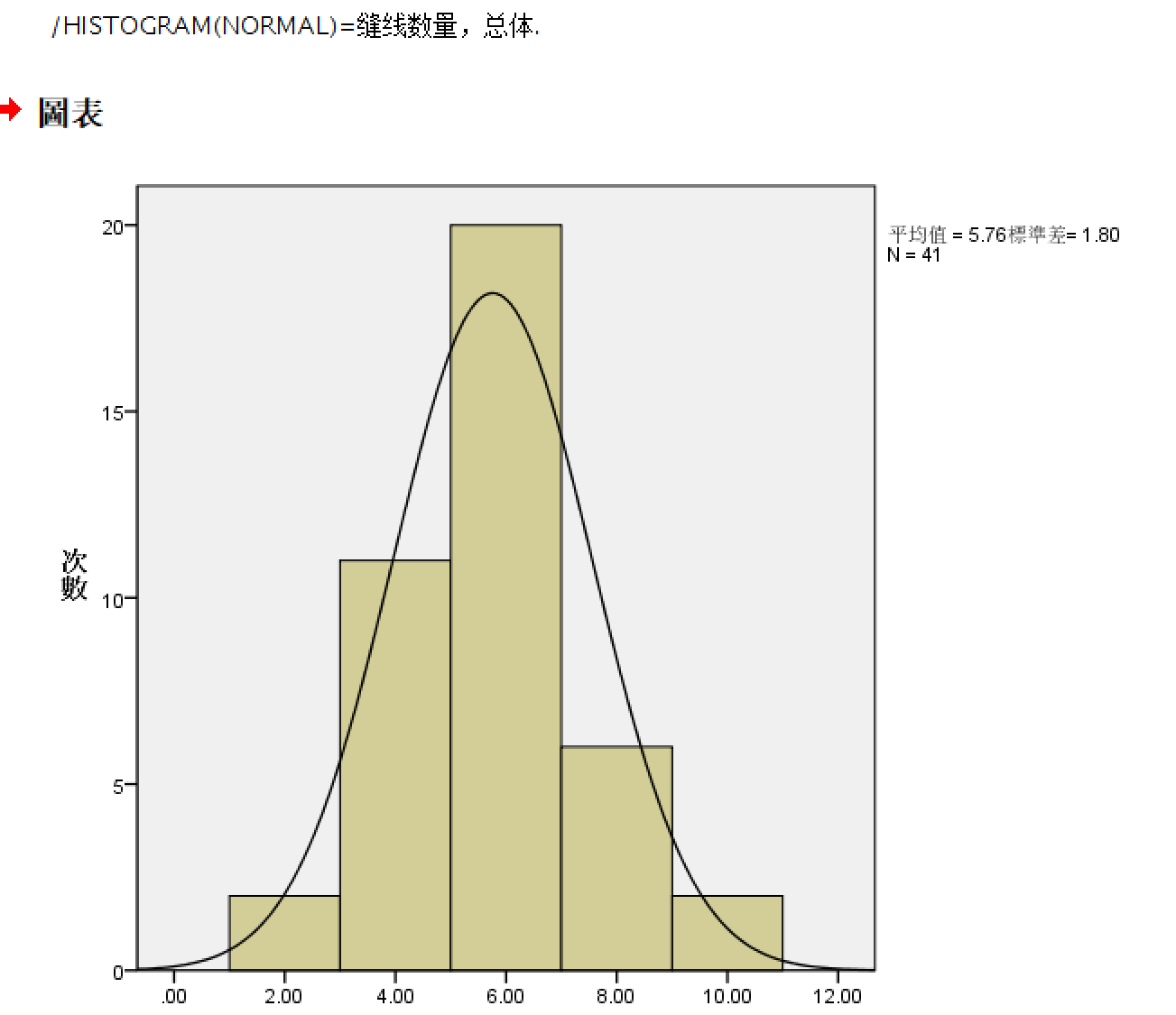

Supplement: Supplementary file 69 — Supplementary file69 (PNG 38 KB) [file 43465_2025_1645_MOESM69_ESM.png]

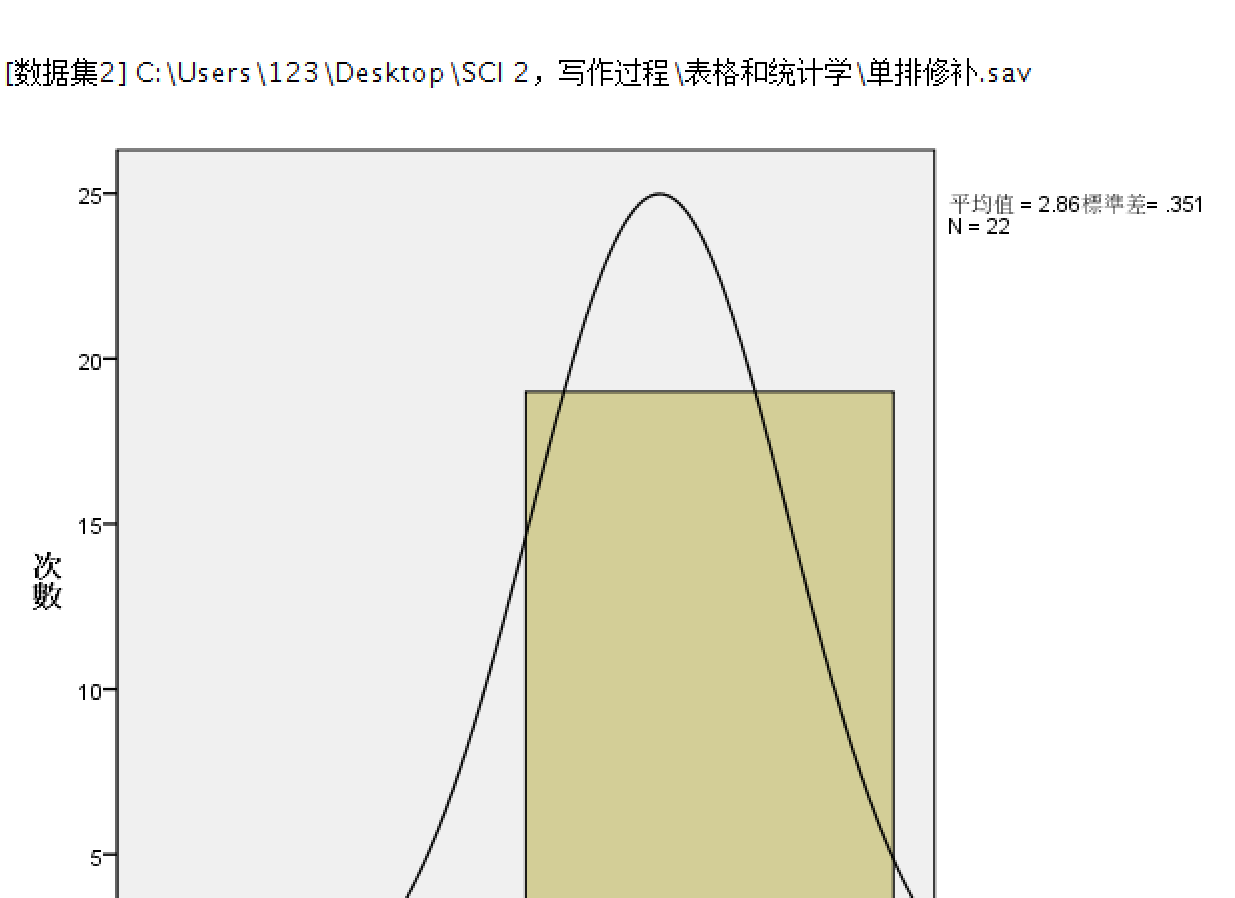

Supplement: Supplementary file 70 — Supplementary file70 (PNG 43 KB) [file 43465_2025_1645_MOESM70_ESM.png]

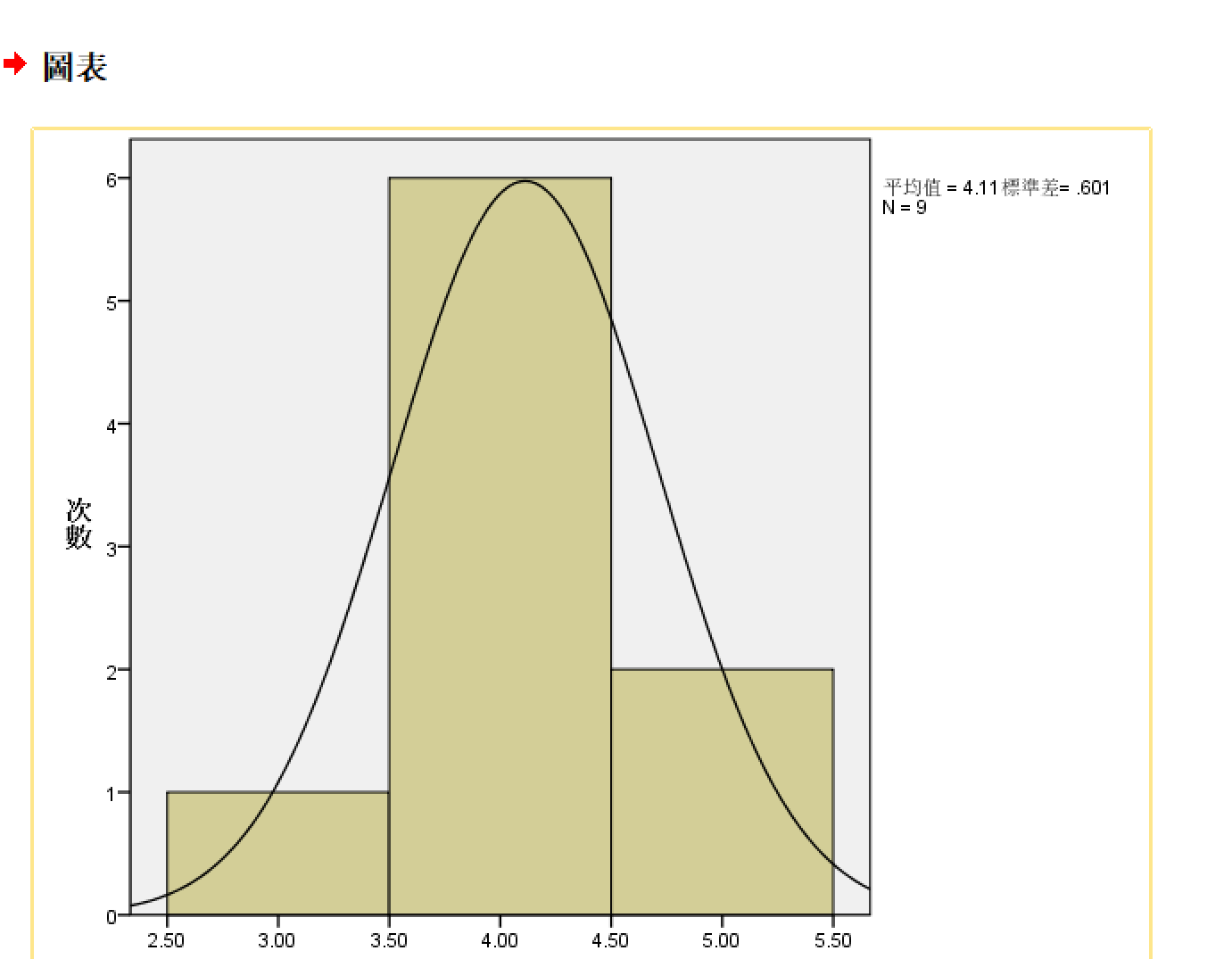

Supplement: Supplementary file 71 — Supplementary file71 (PNG 34 KB) [file 43465_2025_1645_MOESM71_ESM.png]

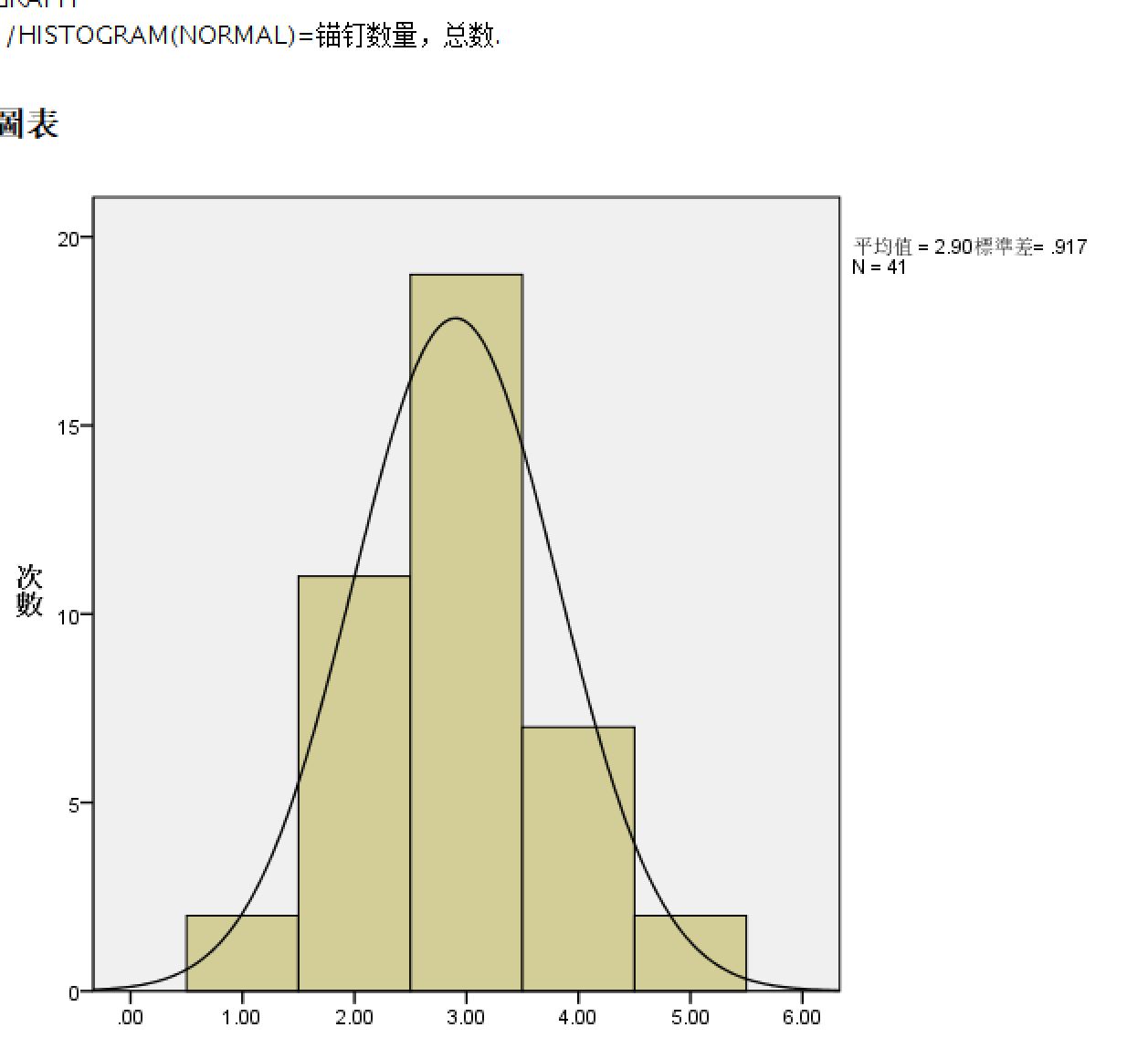

Supplement: Supplementary file 72 — Supplementary file72 (PNG 45 KB) [file 43465_2025_1645_MOESM72_ESM.png]

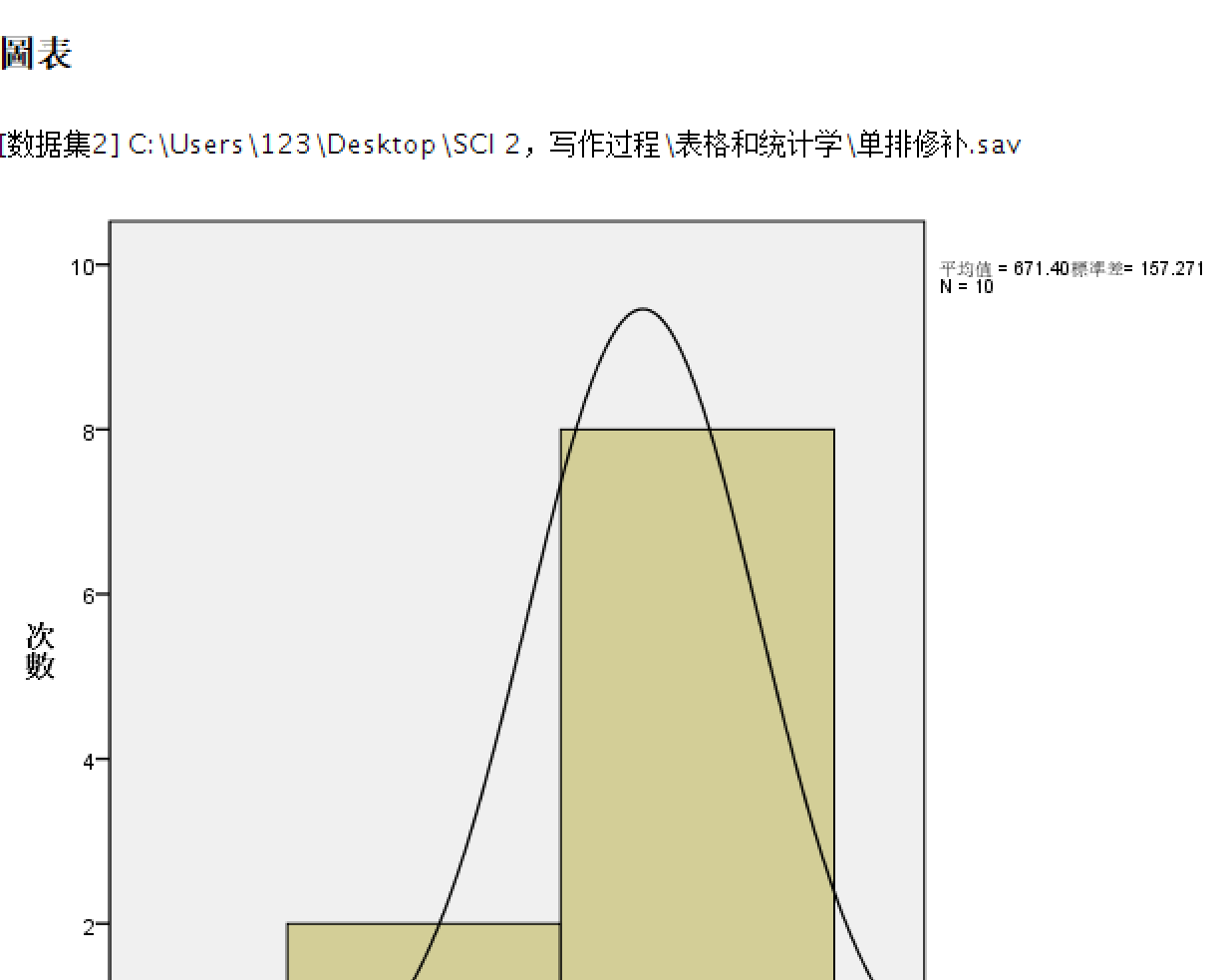

Supplement: Supplementary file 73 — Supplementary file73 (PNG 46 KB) [file 43465_2025_1645_MOESM73_ESM.png]

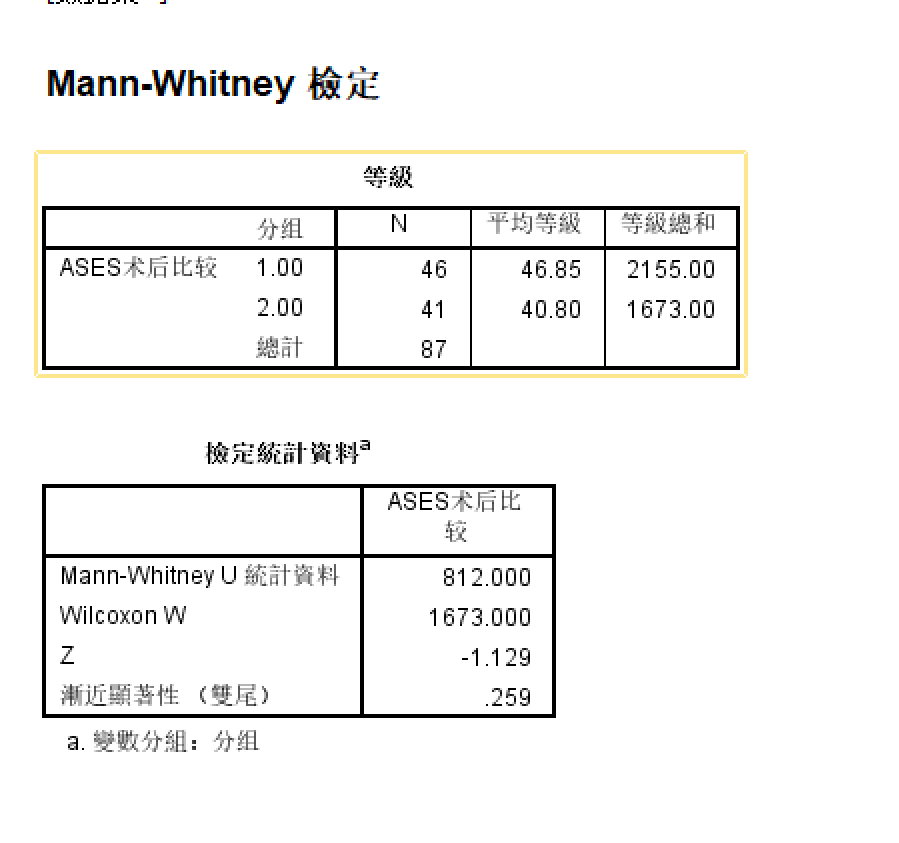

Supplement: Supplementary file 74 — Supplementary file74 (PNG 42 KB) [file 43465_2025_1645_MOESM74_ESM.png]

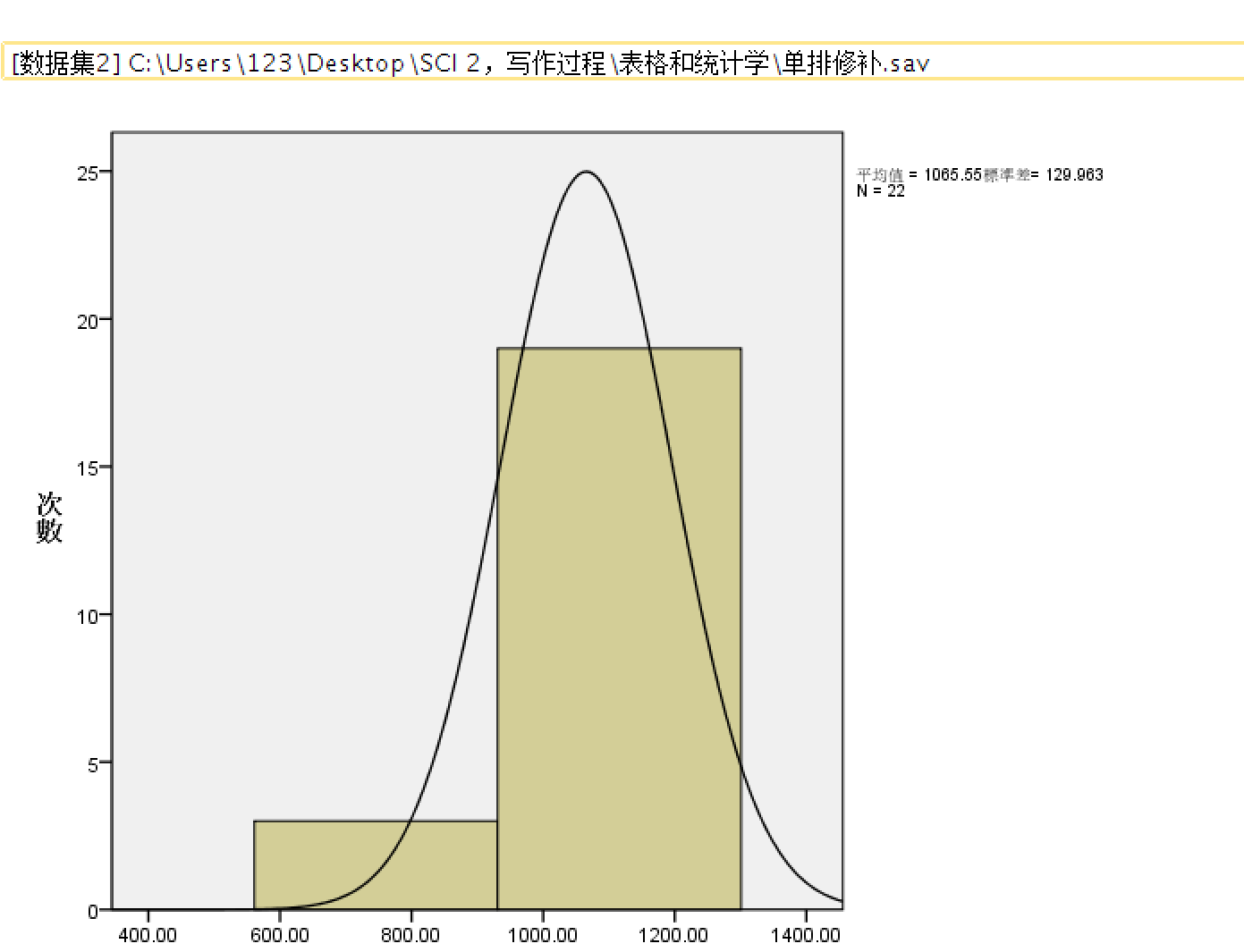

Supplement: Supplementary file 75 — Supplementary file75 (PNG 43 KB) [file 43465_2025_1645_MOESM75_ESM.png]

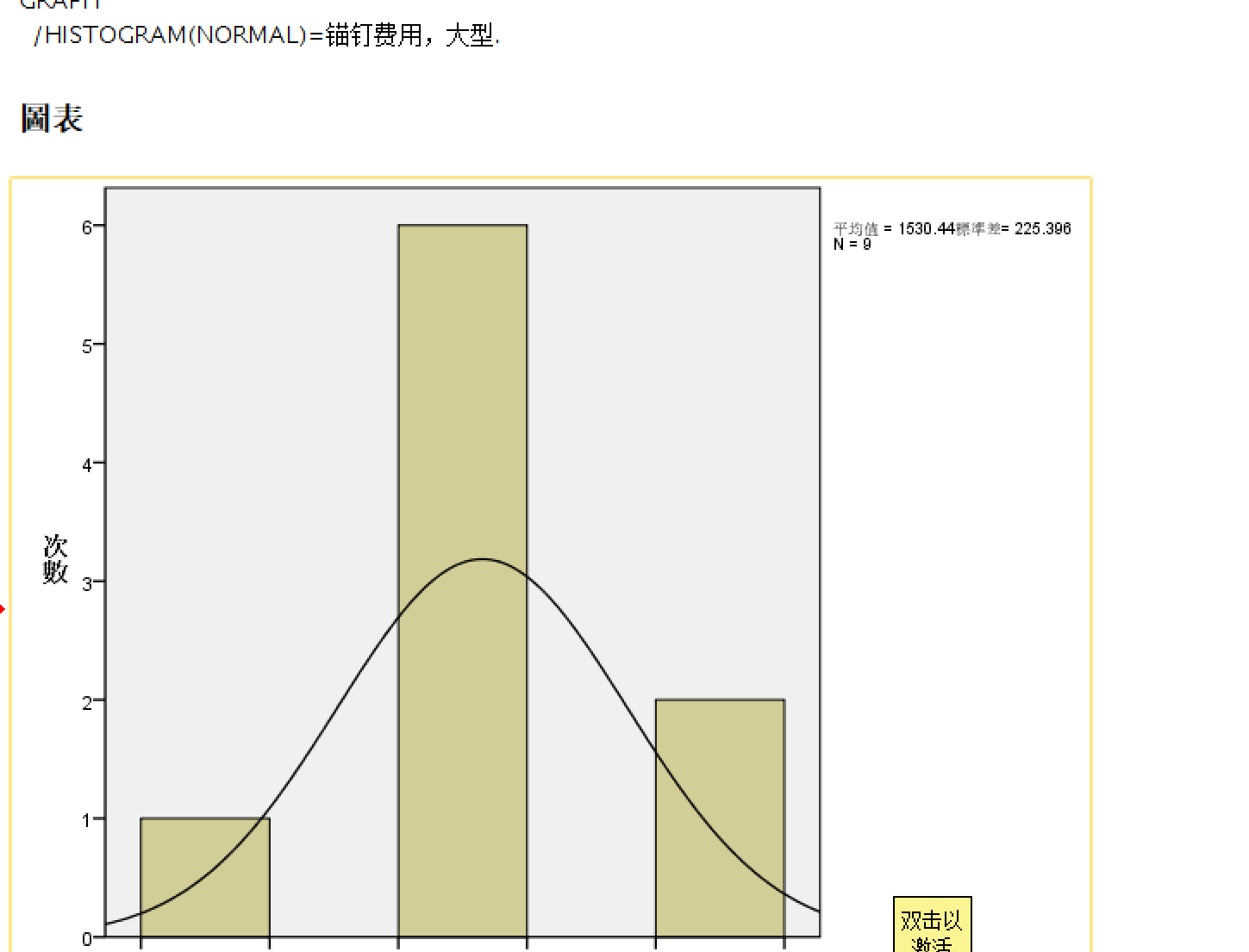

Supplement: Supplementary file 76 — Supplementary file76 (PNG 41 KB) [file 43465_2025_1645_MOESM76_ESM.png]

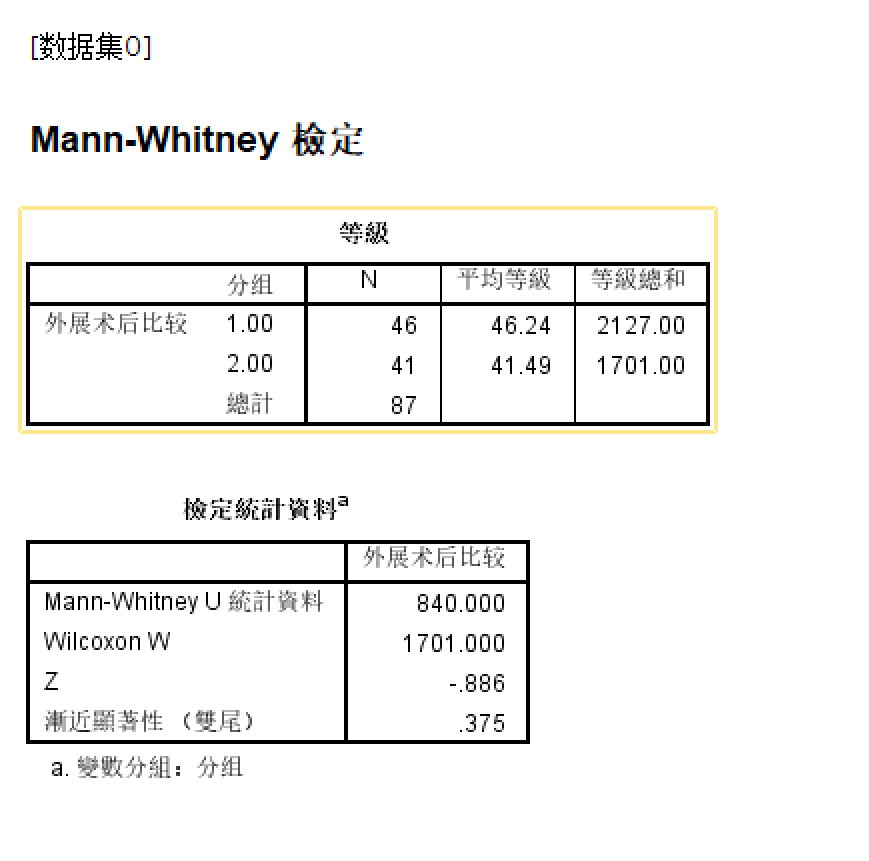

Supplement: Supplementary file 77 — Supplementary file77 (PNG 41 KB) [file 43465_2025_1645_MOESM77_ESM.png]

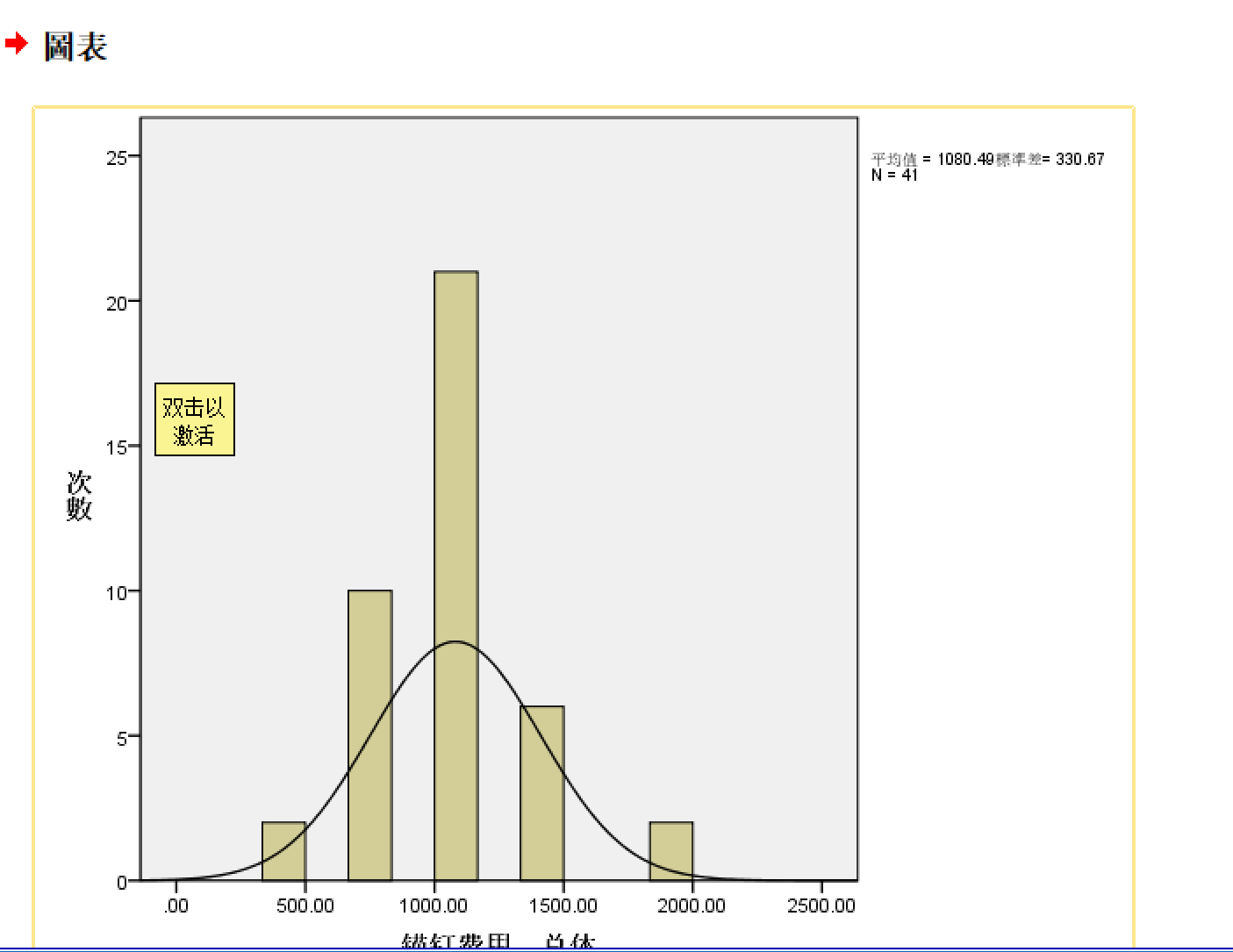

Supplement: Supplementary file 78 — Supplementary file78 (PNG 42 KB) [file 43465_2025_1645_MOESM78_ESM.png]

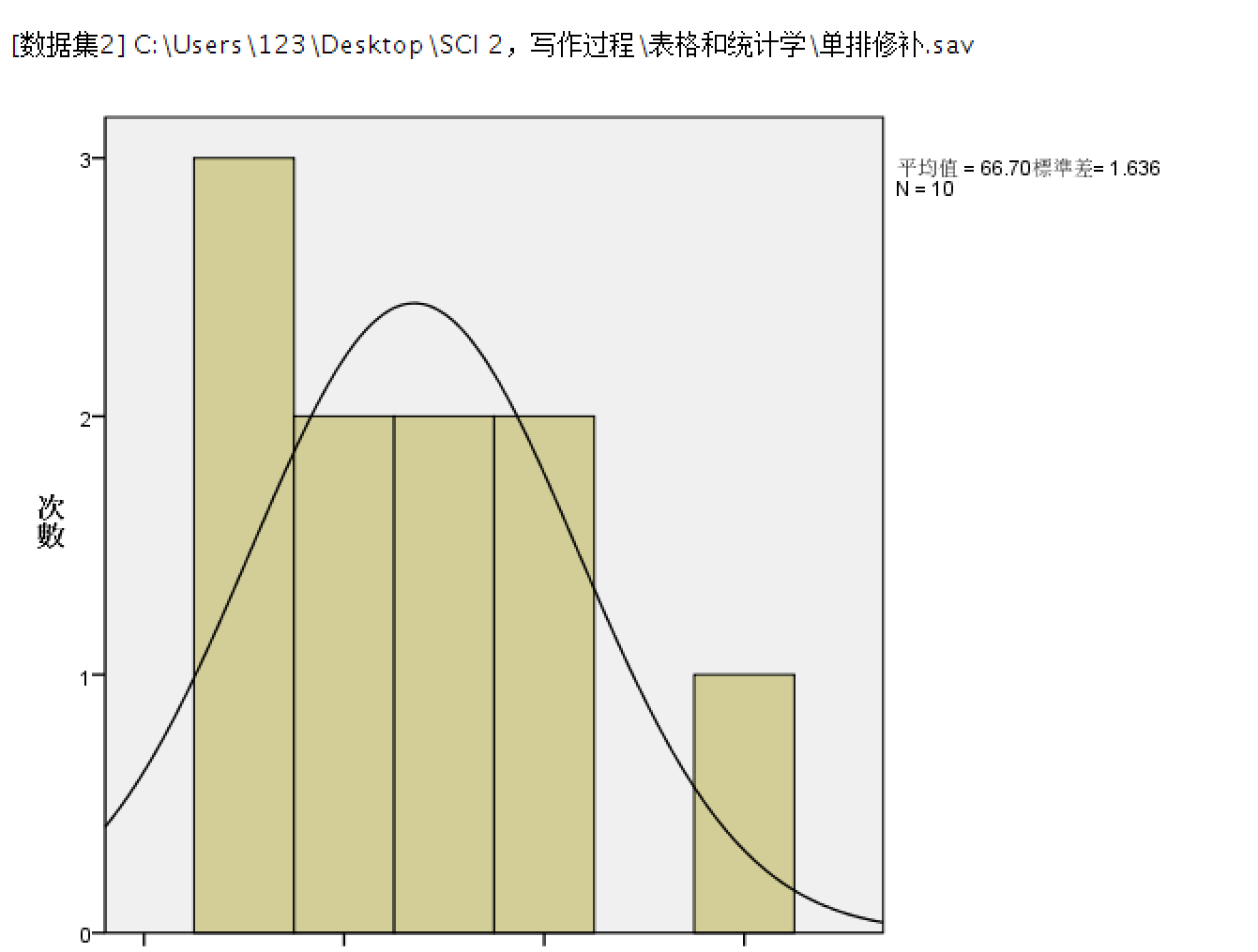

Supplement: Supplementary file 79 — Supplementary file79 (PNG 43 KB) [file 43465_2025_1645_MOESM79_ESM.png]

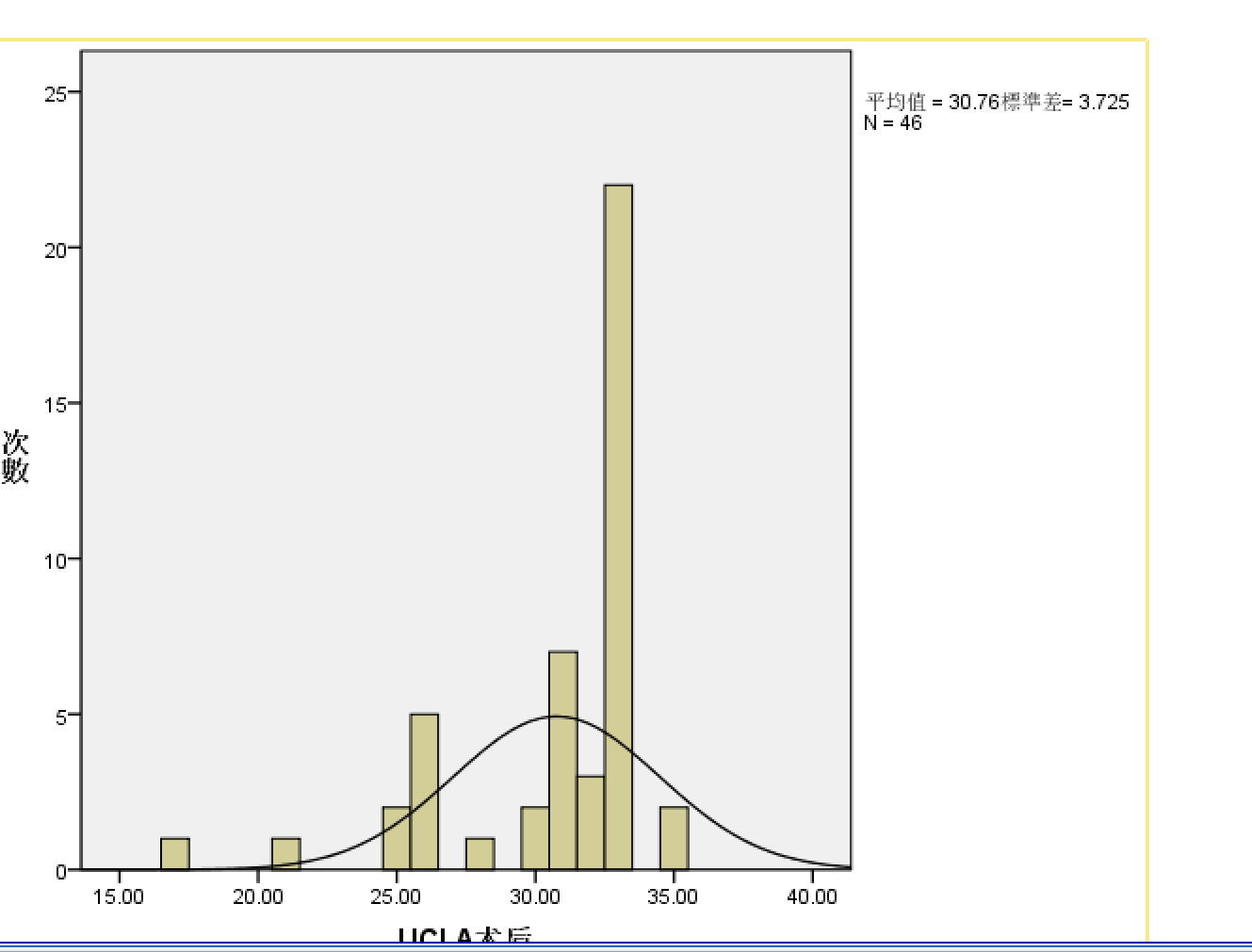

Supplement: Supplementary file 80 — Supplementary file80 (PNG 51 KB) [file 43465_2025_1645_MOESM80_ESM.png]

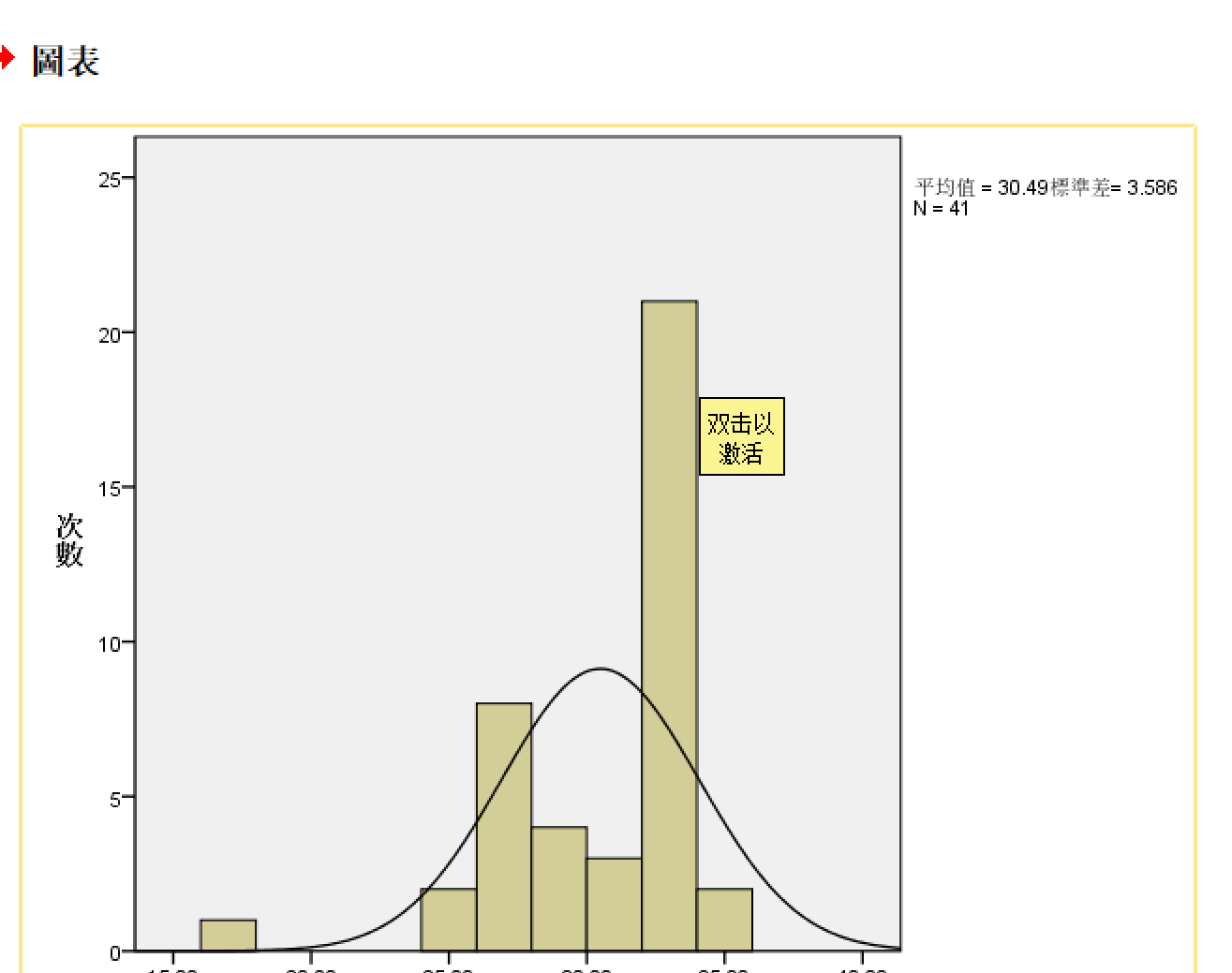

Supplement: Supplementary file 81 — Supplementary file81 (PNG 41 KB) [file 43465_2025_1645_MOESM81_ESM.png]

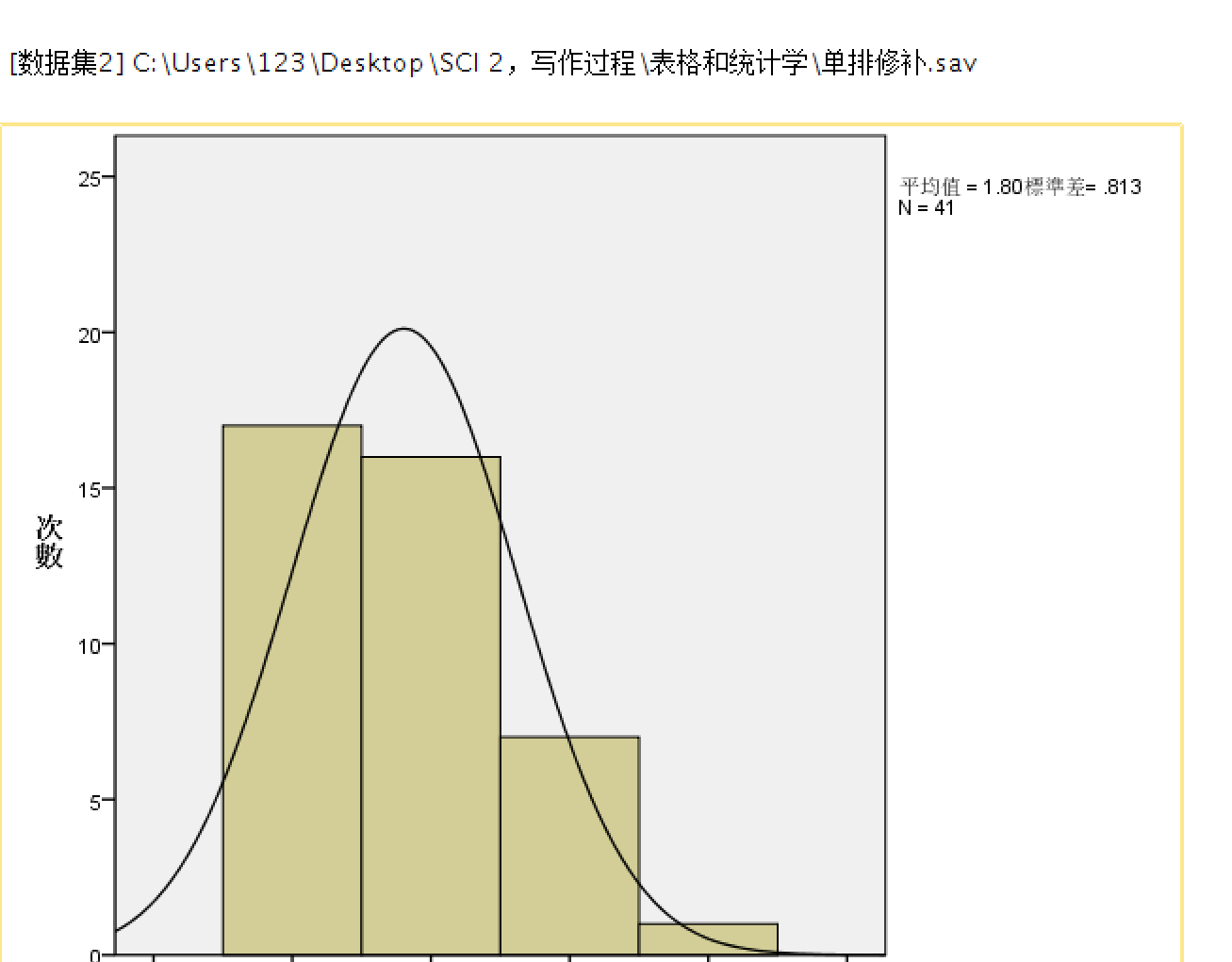

Supplement: Supplementary file 82 — Supplementary file82 (PNG 36 KB) [file 43465_2025_1645_MOESM82_ESM.png]

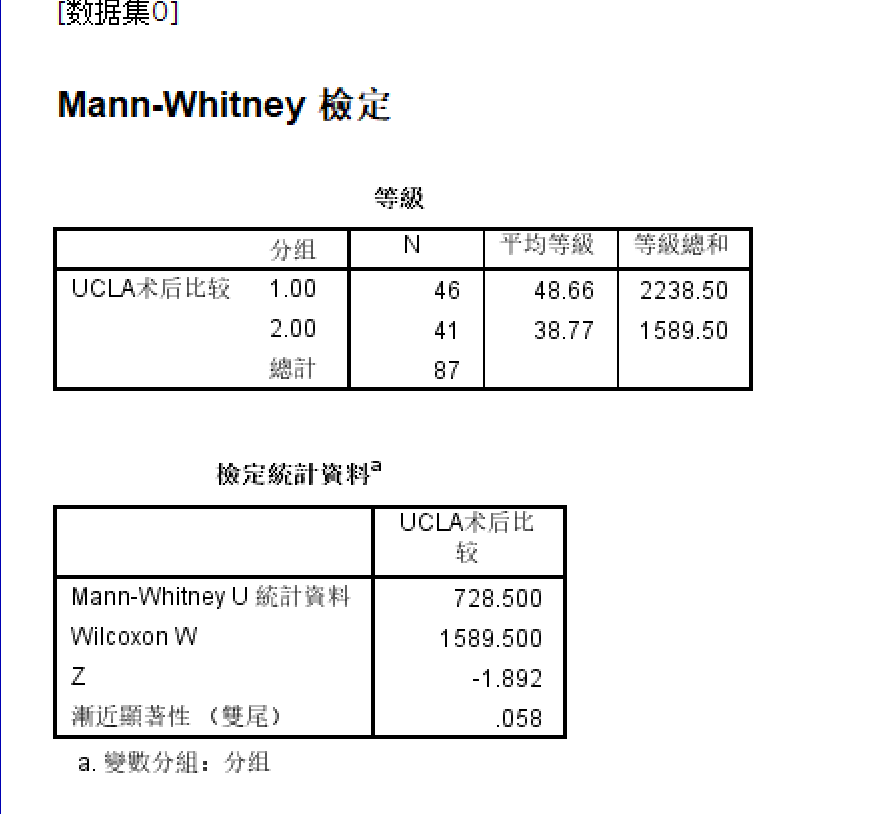

Supplement: Supplementary file 83 — Supplementary file83 (PNG 46 KB) [file 43465_2025_1645_MOESM83_ESM.png]

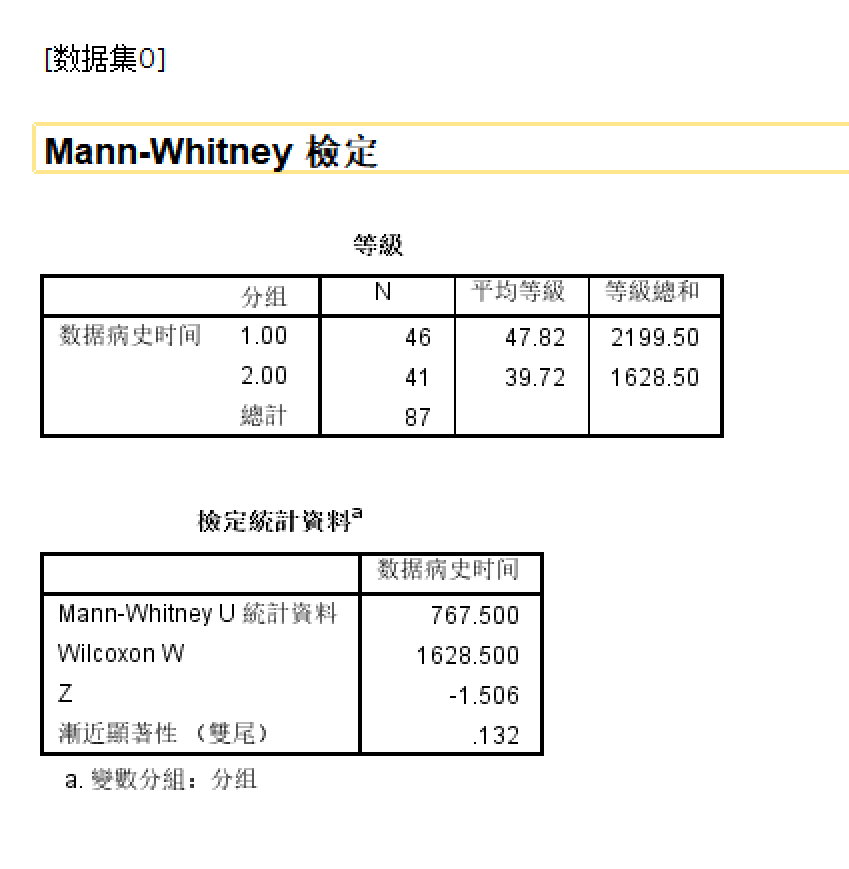

Supplement: Supplementary file 84 — Supplementary file84 (PNG 32 KB) [file 43465_2025_1645_MOESM84_ESM.png]

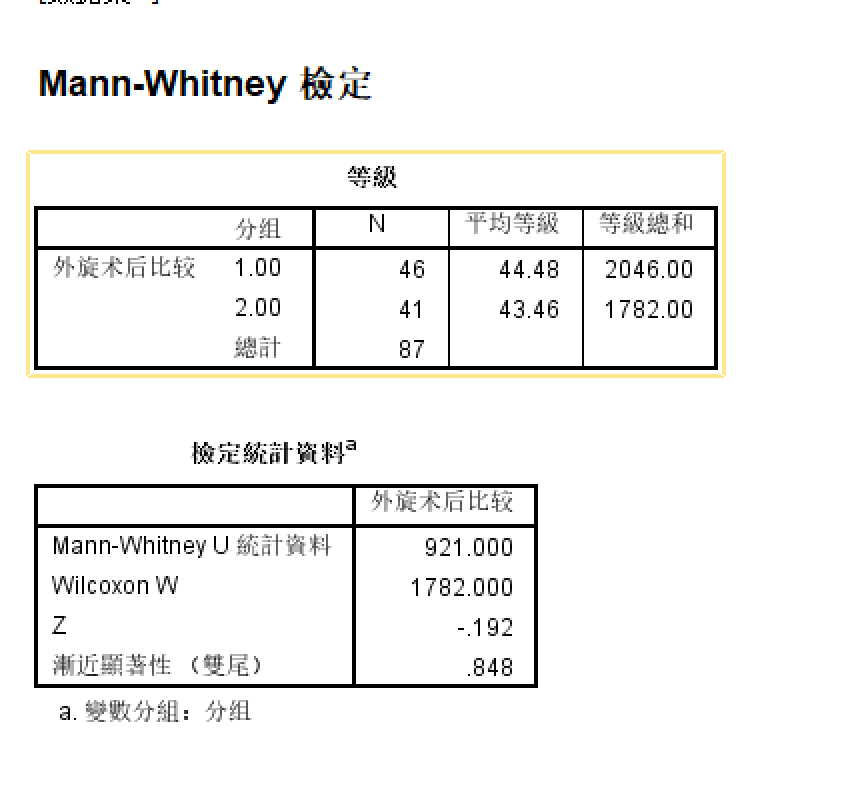

Supplement: Supplementary file 85 — Supplementary file85 (PNG 34 KB) [file 43465_2025_1645_MOESM85_ESM.png]

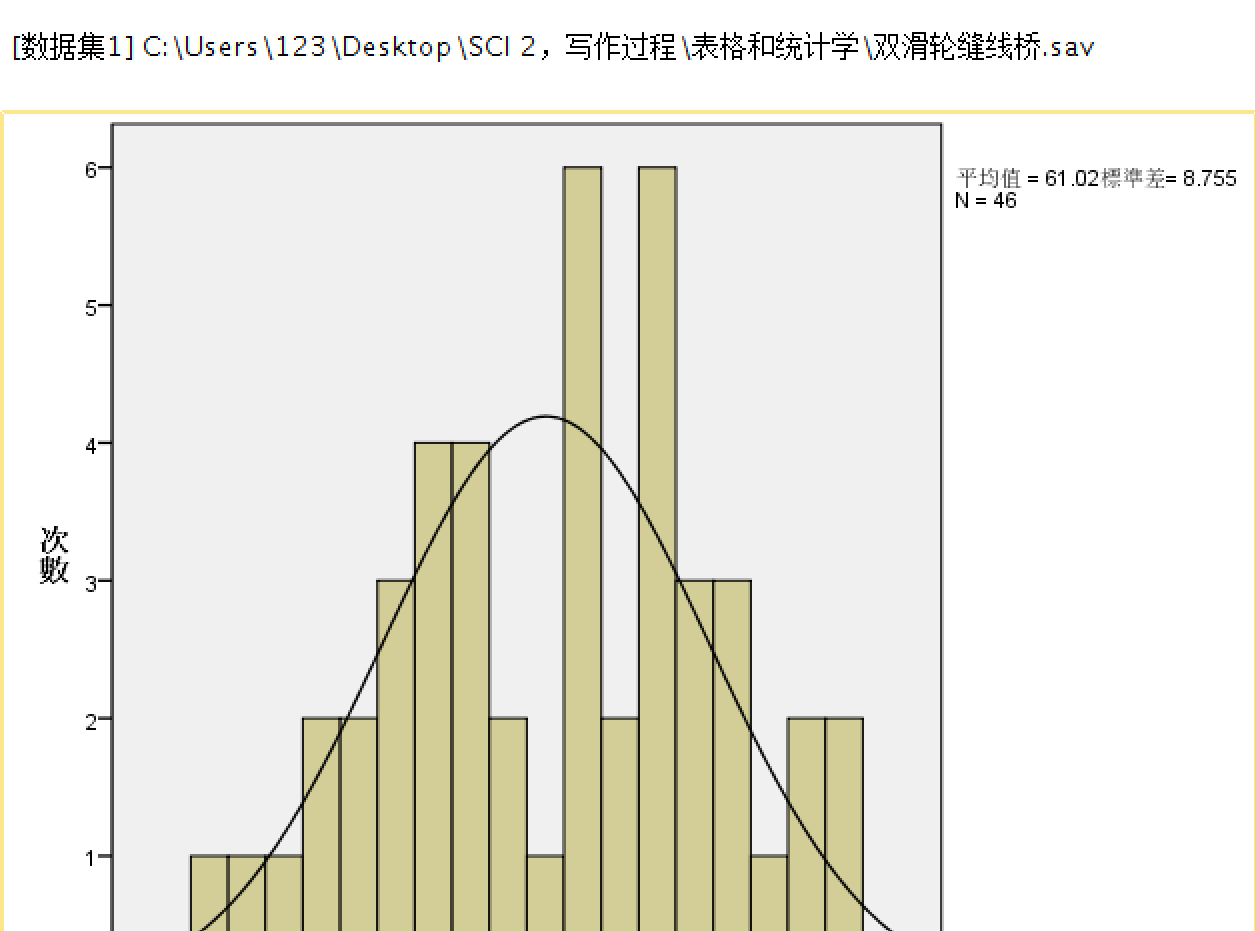

Supplement: Supplementary file 86 — Supplementary file86 (PNG 44 KB) [file 43465_2025_1645_MOESM86_ESM.png]

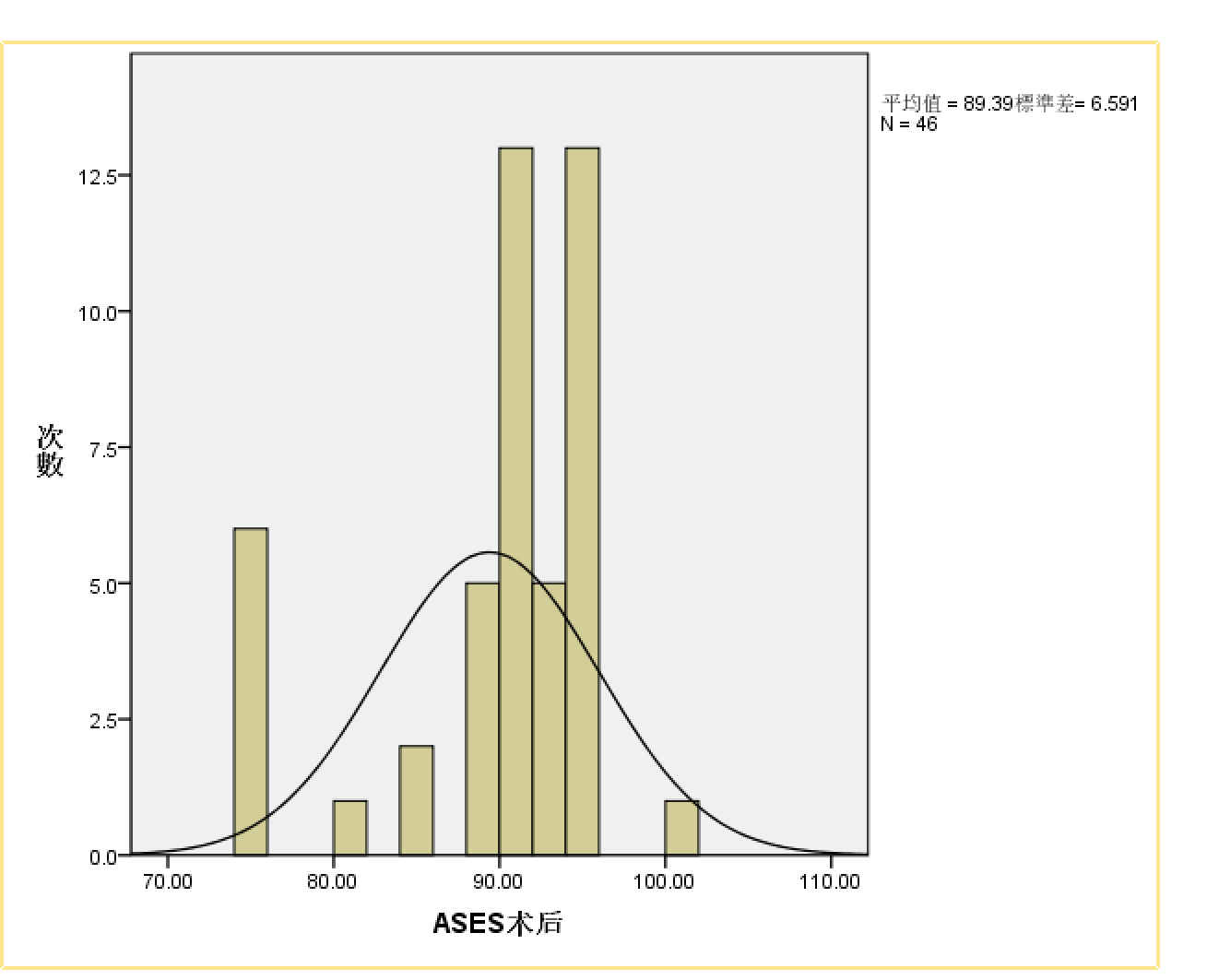

Supplement: Supplementary file 87 — Supplementary file87 (PNG 41 KB) [file 43465_2025_1645_MOESM87_ESM.png]
